# Supplementary material for: Differences in birth weight between immigrants’ and natives’ children in Europe and Australia: a LifeCycle comparative observational cohort study
Source: BMJ Open. 2023 Mar 23;13(3):e060932. doi: 10.1136/bmjopen-2022-060932 (PMC10040079; doi:10.1136/bmjopen-2022-060932)
Supplement: Supplementary data [file bmjopen-2022-060932supp001.pdf]

Appendix 1 Cohort sample distribution & children's birthweight (in grams) by migration status and mother's region of origin (cohorts with small samples)

	GECKO (NL)			INMA (SP)			NINFEA (IT)			Piccoli Piu (IT)		
	Freq.	%	Weight Mean	Freq.	%	Weight Mean	Freq.	%	Weight Mean	Freq.	%	Weight Mean
**Children's migration status**												
1st generation	0	0	---	0	0	---	16	0.4	3539	0	0	---
2nd generation	26	0.9	3414	125	5.5	3343	28	0.8	3390	123	3.7	3413
2.5 generation (mother)	60	2.1	3428	88	3.9	3321	110	3.0	3294	151	4.5	3376
2.5 generation (father)	64	2.3	3578	117	5.2	3164	95	2.6	3202	106	3.2	3388
natives	2445	86.0	3549	1791	78.9	3252	3348	91.1	3271	2881	85.8	3327
Missing	247	8.7	3484	20	0.9	3486	59	1.6	3240	95	2.8	3297
**Total**	**2842**	**100**	**3540**	**2141**	**100**	**3258**	**3675**	**100**	**3272**	**3356**	**100**	**3334**
**Mother's region of birth**												
Host country-born	2548	89.7	3548	1913	89.4	3247	3516	96.2	3244	3031	90.3	3329
Western EU/EEA	16	0.6	3503	29	1.35	3301	50	1.4	3430	41	1.2	3328
Eastern EU	7	0.2	3546	19	0.89	3186	32	0.9	3628	109	3.3	3426
Other Europe & Central Asia	11	0.4	3558	6	0.28	3127	12	0.3	2550	38	1.1	3421
East Asia & Pacific	18	0.6	3371	1	0.05	3210	1	0	2750	6	0.2	3372
South Asia	7	0.2	3388	0	0	---	1	0	3385	4	0.1	3099
Middle East & North Africa	9	0.3	3490	4	0.19	3415	3	0.1	2945	16	0.5	3427
Sub-Saharan Africa	6	0.2	3501	3	0.14	3122	2	0.1	3253	4	0.1	3239
Latin America & Caribbean	16	0.6	3161	151	7.05	3373	37	1	3225	54	1.6	3338
North America	1	0.0	4460	0	0	---	2	0.1	---	6	0.2	3748
Missing	203	7.1	3495	15	0.7	3481	0	0	3272	47	1.4	3288
**Total**	**2842**	**100**	**3540**	**2141**	**100**	**3258**	**3656**	**100**		**3356**	**100**	**3334**
***n* Missing cases in analyses**	***n***		**Mean**	***n***		**Mean**	***n***		**Mean**	***n***		**Mean**
Birthweight	50		---	21		---	69		---	19		---
Child & mother's controls	438		3486	118		3354	181		3315	50		3409
Migration status	39		3439	7		3294	51		3235	71		3277
Missing other variables	86		3458	97		3059	148		3313	175		3310
**Total excluded cases**	**613**		**3479**	**243**		**3222**	**362**		**3304**	**315**		**3319**

Appendix 2 OLS regression coefficients of mother's region of origin & child's migrant status on child's birthweight (Small cohorts - extracts)

	GECKO (NL)						INMA (SP)					
**DV1: Birthweight**	**M1**		**M2**		**M3**		**M1**		**M2**		**M3**	
	**Child controls**		**SES**		**Smoke**		**Child controls**		**SES**		**Smoke**	
**Child's migrant status**												
Natives (ref.)												
1st Generation	-		-		-		-		-		-	
2nd Generation	-139.76		-117.46		-156.54		162.68	***	169.51	***	143.41	***
2.5 Generation - mother	101.21		99.87		79.96		70.50		78.87		75.32	
2.5 generation - father	16.45		16.31		12.30		21.46		28.86		29.50	
**Constant**	**-5767.43**	***	**-5738.24**	***	**-5690.72**	***	**-4847.76**	***	**-4734.01**	***	**-4723.00**	***
**N**	**2229**		**2229**		**2229**		**1898**		**1898**		**1898**	
**R2**	0.4023		0.4057		0.4238		0.333		0.338		0.344	
**Mother's region of origin**												
Host country-born (ref.)												
Western EU/EEA	49.22		61.92		60.12		-31.75		-14.98		-22.09	
Eastern EU	176.27		171.70		147.02		175.44		182.01		168.67	
Other Europe & Central Asia	255.46		277.51		260.90		-122.58		-110.36		-106.48	
East Asia & Pacific	21.84		21.53		2.13		-		-		-	
South Asia	113.32		148.45		88.23		-		-		-	
Middle East & North Africa	1.50		34.60		-16.44		-		-		-	
Sub-Saharan Africa	58.96		83.10		39.75		-		-		-	
Latin America & Caribbean	-146.75		-161.48		-196.00		173.31	***	176.04	***	155.59	***
North America	-		-		-		-		-		-	
**Constant**	**-5757.77**	***	**-5737.15**	***	**-5671.80**	***	**-4892.39**	***	**-4773.55**	***	**-4763.82**	***
**N**	**2,254**		**2,254**		**2,254**		**1898**		**1898**		**1898**	
**R2**	0.4021		0.4059		0.4238		0.336		0.34		0.346	

**p < .05, **p < .01, ***p < .001.*

Piccolopiù used a binary variable for parity and : Region 1: Western Europe, Region 2: Easter Europe + Other Europe & Central Asia, Region 3: Latin America & Caribbean, Region 4: other regions.

INMA East Asia & Pacific includes Middle East & North Africa and Sub-Saharan Africa due to small number sizes.

M1: Basic child et mother's controls at birth

M2: M1 + socioeconomic variables

M3: M2 + mother's smoking during pregnancy

Appendix 2 Continued

	NINFEA (IT)			PICCOLOPIU (IT)		
**DV1: Birthweight**	**M1**<br>**Child controls**	**M2**<br>**SES**	**M3**<br>**Smoke**	**M1**<br>**Child controls**	**M2**<br>**SES**	**M3**<br>**Smoke**
**Child's migrant status**						
Natives (ref.)						
1st Generation	153.43	147.14	147.89	-	-	-
2nd Generation	118.20	109.99	108.60	118.49 ***	100.96 **	97.18 **
2.5 Generation - mother	41.05	40.23	39.97	56.51	54.19	53.27
2.5 generation - father	-70.12	-73.01	-74.15	20.68	15.51	14.58
**Constant**	**-3887.01** ***	**-3883.26** ***	**-3873.41** ***	**-4584.30** ***	**-4521.88** ***	**-4539.19** ***
**N**	**3216**	**3216**	**3216**	**3041**	**3041**	**3041**
**R2**	0.311	0.312	0.314	0.306	0.308	0.309
**Mother's region of origin**						
Host country-born (ref.)						
Western EU/EEA	-10.95	-4.73	-4.85	36.70	34.20	32.98
Eastern EU	124.89 *	123.63 *	126.67 *	116.58 **	103.41 **	102.47 **
Other Europe & Central Asia	96.77	100.00	97.25			
East Asia & Pacific	-62.01	-58.73	-12.27	65.62	57.29	51.86
South Asia	-6.17	-10.28	-15.99			
Middle East & North Africa	45.25	58.64	53.82			
Sub-Saharan Africa	-187.85	-191.41	-196.06			
Latin America & Caribbean	80.17	79.42	76.13	96.08	87.84	88.07
North America	125.89	134.27	128.67	-	-	-
**Constant**	**-4179.89** ***	**-4185.06** ***	**-4182.61** ***	**-4526.39** ***	**-4459.02** ***	**-4477.76** ***
**N**	**5872**	**5872**	**5872**	**3070**	**3070**	**3070**
**R2**	0.408	0.409	0.41	0.304	0.306	0.307

**p < .05, **p < .01, ***p < .001.*

Appendix 3 OLS regression coefficients of children's migration status on child's birthweight (DV2: excluded birthweight > 4,500g; large cohorts)

	ELFE (FR)			RAINE STUDY (AU)			BIB (UK)		
**DV2: Birthweight**	**M1 Child controls**	**M2 SES**	**M3 Smoke**	**M1 Child controls**	**M2 SES**	**M3 Smoke**	**M1 Child controls**	**M2 SES**	**M3 Smoke**
**Children's migration status**									
Natives (ref.)									
2nd generation	16.4	45.7 **	17.1	46.9 *	40.6	23.8	-80.6 ***	-81.3 ***	-115.9 ***
2.5 generation (mother)	40.4 **	46.2 **	35.8 *	44.8	41.2	36.4	-99.6 ***	-95.9 ***	-133.0 ***
2.5 generation (father)	4.5	17.3	12.3	41.5	39.6	32.0	-108.3 ***	-112.0 ***	-138.2 ***
**Child controls**									
Female	-141.2 ***	-141 ***	-142.1 ***	-113.1 ***	-113.0 ***	-123.3 ***	-131.9 ***	-130.3 ***	-131.5 ***
Plural birth[a]	-372.8 ***	-374.7 ***	-378.4 ***	0	0	0	-310.6 ***	-303.6 ***	-308.5 ***
Mother's parity (birth order)	45.7 ***	50.7 ***	49.8 ***	51.4 ***	51.9 ***	64.7 ***	49.5 ***	56.9 ***	54.2 ***
Gestational age	23.1 ***	22.9 ***	22.9 ***	19.3 ***	19.3 ***	20.1 ***	24.9 ***	25.0 ***	24.9 ***
**Mother controls**									
Height	8.3 ***	7.7 ***	7.9 ***	10.9 ***	10.6 ***	10.8 ***	10.9 ***	10.6 ***	10.8 ***
Pre-pregnancy weight	4.3 ***	4.7 ***	4.5 ***	4.9 ***	5.0 ***	4.9 ***	4.8 ***	4.8 ***	4.8 ***
**SES**									
**Education[b]**									
High (ref.)									
Medium		-15.26	-7.13		12.5	13.9		2.50	4.55
Low		-38.16 *	-19.78		-11.3	7.9		-15.80	-12.71
**Household income quintiles[c]**									
1st quintiles (ref.)									
2nd quintile		46.1 ***	39.1 ***					2.8	-7.8
3th quintile		45.5 ***	29 *		-6.2	3.8		-35.8	-21.1
4th quintile		52.9 ***	33.3 *		-19.2	-3.9		-42.6 *	-31.6
5th quintile		57.3 ***	33.9 *		-23.9	6.2		-70.3 **	-34.0
**Mother smoked (pregnancy)**			-114.6 ***			-140.6 ***			-130.9 ***
**Constant**	**-4729** ***	**-4666** ***	**-4645** ***	**-4058** ***	**-4004** ***	**-4030** ***	**-5699** ***	**-5638** ***	**-5594** ***
N	**12373**	**12373**	**12373**	2242	2242	2242	4090	4090	4090
R^2	0.36	0.36	0.37	0.38	0.38	0.40	0.49	0.49	0.50

**p < .05, **p < .01, ***p < .001*

[a] The ABCD & RAINE STUDY cohorts only included single births in their sample.

[b] Level of education based on the highest on-going or completed education when the child was 0 year old (between >-1 year and <1 year). If more than one education level is reported within the defined time frame, we used highest recorded education level.

Classification according to International Standard Classification of Education 97/2011 (ISCED-97/2011)

High: Short cycle tertiary, Bachelor, Masters, Doctoral or equivalent (ISCED-2011: 5-8, ISCED-97: 5-6)

Medium: Upper secondary, Post-secondary non-tertiary (ISCED-2011: 3-4, ISCED-97: 3-4)

Low: No education; early childhood; pre-primary; primary; lower secondary or second stage of basic education (ISCED-2011: 0-2, ISCED-97: 0-2)

[c] Eusilc income quintiles for all cohorts except for RAINE (AU) and ABCD (NL) that measured household income in quartiles.

M1b: Basic child et mother's controls at birth

M2b: M1b + socioeconomic variables

M3b: M2b + mother's smoking during pregnancy

Appendix 3 Continued

DV2: Birthweight	ABCD (NL) M1 Child controls		ABCD (NL) M2 SES		ABCD (NL) M3 Smoke		GEN R (NL) M1 Child controls		GEN R (NL) M2 SES		GEN R (NL) M3 Smoke	
**Children's migration status**												
Natives (ref.)												
2nd generation	-84.7	***	-58.9	**	-71.3	***	-69.35	***	-33.1		-48.1	*
2.5 generation (mother)	19.8		27.5		20.1		36.18		48.2	*	42.6	*
2.5 generation (father)	-73.2	***	-61.1	**	-57.8	**	-61.37	***	-41.1	*	-38.5	*
**Child controls**												
Female	-103.2	***	-104.7	***	-104.9	***	-91.9	***	-92.4	***	-93.4	***
Plural birth[a]	0		0		0		-405.8	***	-409.5	***	-409.9	***
Mother's parity (birth order)	72.8	***	74.7	***	73.8	***	98.8	***	102.2	***	99.9	***
Gestational age	24.4	***	24.4	***	24.3	***	24.9	***	24.8	***	24.7	***
**Mother controls**												
Height	8.8	***	8.0	***	8.0	***	9.3	***	8.4	***	8.5	***
Pre-pregnancy weight	3.8	***	4.1	***	4.1	***	3.9	***	4.1	***	4.1	***
**SES**												
**Education**[b]												
High (ref.)												
Medium			-54.6	***	-43.8	**			-11.0		-8.7	
Low			-59.6	**	-38.1				-19.9		-12.9	
**Household income quintiles**[c]												
1st quintiles (ref.)												
2nd quintile			5.0		7.9				27.4		21.9	
3th quintile			-6.9		2.0				60.3	**	48.5	*
4th quintile			-24.1		-12.9				85.2	**	66.5	*
5th quintile			-		-				79.2	*	60.2	
**Mother smoked (pregnancy)**					-119.0	***					-71.2	***
**Constant**	**-5090**	***	**-4941**	***	**-4925**	***	**-5462**	***	**-5338**	***	**-5304**	***
N	3,918		3,918		3,918		4751		4751		4751	
**R^2**	0.40		0.40		0.41		0.46		0.46		0.47	

**p < .05, **p < .01, ***p < .001*

Appendix 4 Logistic regression odds ratios of children's migration status on child's birthweight (DV3: low birthweight; large cohorts)

DV3: Low birthweight	ELFE (FR)			RAINE STUDY (AU)			BIB (UK)		
	M1 Child controls	M2 SES	M3 Smoke	M1 Child controls	M2 SES	M3 Smoke	M1 Child controls	M2 SES	M3 Smoke
**Children's migration status**									
Natives (ref.)									
2nd generation	-0.38	-0.61	-0.35	-0.68 *	-0.60	-0.50	0.39	0.01	0.01
2.5 generation (mother)	-0.26	-0.35	-0.20	-0.06	0.01	0.06	0.23	0.00	0.01
2.5 generation (father)	-0.11	-0.18	-0.14	-0.17	-0.11	-0.05	0.85 ***	0.03 **	0.03 ***
**Child controls**									
Female	0.63 ***	0.63 ***	0.65 ***	0.21	0.22	0.20	-0.44 **	0.01 *	0.02 **
Plural birth[a]	1.59 ***	1.60 ***	1.66 ***	0.00	0.00	0.00	1.45 ***	0.27 ***	0.27 ***
Mother's parity (birth order)	-0.14 *	-0.20 **	-0.21 **	-0.32 **	-0.31 **	-0.32 **	-0.14 .	-0.02 ***	-0.01 ***
Gestational age	-0.15 ***	-0.15 ***	-0.15 ***	-0.12 ***	-0.12 ***	-0.12 ***	-0.15 ***	-0.01 ***	-0.01 ***
**Mother controls**									
Height	-0.03 **	-0.02 *	-0.02 *	-0.06 **	-0.06 **	-0.06 **	-0.06 ***	0.00 ***	0.00 ***
Pre-pregnancy weight	-0.02 ***	-0.02 ***	-0.02 ***	0.00	0.00	0.00	-0.02 **	0.00 *	0.00 *
**SES**									
**Education[b]**									
High (ref.)									
Medium		0.16	0.11		0.09	0.07		-0.01	-0.01
Low		0.50 *	0.38		0.36	0.25		0.00	0.00
**Household income quintiles[c]**									
1st quintiles (ref.)									
2nd quintile		-0.25	-0.20					-0.01	-0.01
3th quintile		-0.24	-0.10		0.02	-0.07		0.01	0.01
4th quintile		-0.31	-0.11		-0.31	-0.43		0.01	0.01
5th quintile		-0.53 *	-0.32		-0.03	-0.26		0.01	0.00
**Mother smoked (pregnancy)**			0.82 ***			0.72 **			0.01
**Constant**	**41.97** ***	**41.23** ***	**41.62** ***	**37.64** ***	**37.37** ***	**37.77** ***	**49.40** ***	**3.53** ***	**3.53** ***
N	12494	12494	12494	2283	2283	2283	4132	4132	4132
**ll**	-1294	-1283.8	-1265.3	-276.92	-269.25	-263.76			

**p* < .05, ***p* < .01, ****p* < .001

[a] The ABCD & RAINE STUDY cohorts only included single births in their sample.

[b] Level of education based on the highest on-going or completed education when the child was 0 year old (between >-1 year and <1 year). If more than one education level is reported within the defined time frame, we used highest recorded education level.

Classification according to International Standard Classification of Education 97/2011 (ISCED-97/2011)

High: Short cycle tertiary, Bachelor, Masters, Doctoral or equivalent (ISCED-2011: 5-8, ISCED-97: 5-6)

Medium: Upper secondary, Post-secondary non-tertiary (ISCED-2011: 3-4, ISCED-97: 3-4)

Low: No education; early childhood; pre-primary; primary; lower secondary or second stage of basic education (ISCED-2011: 0-2, ISCED-97: 0-2)

[c] Eusilc income quintiles for all cohorts except for RAINE (AU) and ABCD (NL) that measured household income in quartiles.

M1b: Basic child et mother's controls at birth

M2b: M1b + socioeconomic variables

M3b: M2b + mother's smoking during pregnancy

Appendix 4 Continued

DV3: Low birthweight	ABCD (NL)			GEN R (NL)		
	M1 Child controls	M2 SES	M3 Smoke	M1 Child controls	M2 SES	M3 Smoke
**Children's migration status**						
Natives (ref.)						
2nd generation	0.67 *	0.42	0.61	0.37	0.37	0.44
2.5 generation (mother)	-0.35	-0.39	-0.30	-0.18	-0.14	-0.16
2.5 generation (father)	0.43	0.25	0.28	0.58 *	0.56 *	0.53 *
**Child controls**						
Female	0.63 **	0.63 **	0.68 **	0.39 *	0.38 *	0.38 *
Plural birth[a]	0.00	0.00	0.00	2.05 ***	2.03 ***	2.03 ***
Mother's parity (birth order)	-0.55 ***	-0.57 ***	-0.55 **	-0.37 **	-0.39 **	-0.37 *
Gestational age	-0.16 ***	-0.16 ***	-0.16 ***	-0.16 ***	-0.16 ***	-0.16 ***
**Mother controls**						
Height	-0.04 *	-0.03	-0.04	-0.05 ***	-0.04 **	-0.05 **
Pre-pregnancy weight	-0.01	-0.01	-0.01	-0.02 *	-0.02 *	-0.02 *
**SES**						
**Education**[b]						
High (ref.)						
Medium		0.32	0.13		-0.26	-0.26
Low		0.18	-0.12		-1.16 *	-1.19 *
**Household income quintiles**[c]						
1st quintiles (ref.)						
2nd quintile		0.12	0.10		-0.07	-0.05
3th quintile		0.68 *	0.60		-0.33	-0.27
4th quintile		0.48	0.41		-1.07 *	-0.96 *
5th quintile					-0.34	-0.23
**Mother smoked (pregnancy)**			1.28 ***			0.31
**Constant**	**45.15** ***	**44.16** ***	**45.66** ***	**48.74** ***	**48.80** ***	**48.87** ***
N	4,032	4,032	4,032	4877	4877	4877
**ll**	-325.45	-321.52	-313.42	-480.1	-473.67	-472.55

**p < .05, **p < .01, ***p < .001*
