# Supplementary material for: Differences in birth weight between immigrants’ and natives’ children in Europe and Australia: a LifeCycle comparative observational cohort study
Source: BMJ Open. 2023 Mar 23;13(3):e060932. doi: 10.1136/bmjopen-2022-060932 (PMC10040079; doi:10.1136/bmjopen-2022-060932)
Supplement: Author's manuscript [file bmjopen-2022-060932.draft_revisions.pdf]

BMJ Open is committed to open peer review. As part of this commitment we make the peer review history of every article we publish publicly available.

When an article is published we post the peer reviewers' comments and the authors' responses online. We also post the versions of the paper that were used during peer review. These are the versions that the peer review comments apply to.

The versions of the paper that follow are the versions that were submitted during the peer review process. They are not the versions of record or the final published versions. They should not be cited or distributed as the published version of this manuscript.

BMJ Open is an open access journal and the full, final, typeset and author-corrected version of record of the manuscript is available on our site with no access controls, subscription charges or pay-per-view fees (http://bmjopen.bmj.com).

If you have any questions on BMJ Open's open peer review process please email info.bmjopen@bmj.com

BMJ Open

Children of migrants' birthweight in Europe and Australia: A LifeCycle comparative study

Journal:	*BMJ Open*
Manuscript ID	bmjopen-2022-060932
Article Type:	Original research
Date Submitted by the Author:	17-Jan-2022

Complete List of Authors:	Florian, Sandra; INSERM,<br>Ichou, Mathieu; INED<br>Panico, Lidia; INED<br>Pinel-Jacquemin, Stéphanie; INED<br>Vrijkotte, Tanja G. M.; Amsterdam Public Health Research Institute, Department of Public Occupational Health<br>Harskamp-van Ginkel, Margreet; Amsterdam Public Health Research Institute, Department of Public Occupational Health<br>Huang, Rae-Chi; University of Western Australia<br>Carson, Jennie; University of Western Australia, Telethon Kids Institute<br>Rodriguez, Loreto; Ministry of Health of the Basque Government, Sub Directorate for Public Health and Addictions of Gipuzkoa; Biodonostia Health Research Institute, Group of Environmental Epidemiology and Child Development<br>Subiza-Pérez, Mikel; Biodonostia Health Research Institute, Group of Environmental Epidemiology and Child Development; Instituto de Salud Carlos III, Spanish Consortium for Research on Epidemiology and Public Health (CIBERESP)<br>Vrijheid, Martine; CIBERESP, Instituto de Salud Carlos III; ISGlobal<br>Fernández-Barrés, Silvia; CIBERESP, Instituto de Salud Carlos II; ISGlobal<br>Yang, Tiffany; Bradford Institute for Health Research, Born in Bradford<br>Corpeleijn, Eva; University of Groningen, Department of Epidemiology, GECKO Drenthe Cohort, University Medical Center Groningen<br>Cardol, Marloes; University of Groningen, Department of Epidemiology, GECKO Drenthe Cohort, University Medical Center Groningen<br>Isaevska, Elena; Universita degli Studi di Torino Dipartimento di Scienze Mediche<br>Moccia, Chiara; University of Turin, Department of Medical Sciences<br>Kooijman, Marjolein; Erasmus Medical Center, The Generation R Study Group, University Medical Center; Erasmus Medical Centre Department of General Pediatrics, University Medical Center<br>Voerman, Ellis; Erasmus Medical Center, The Generation R Study Group, University Medical Center; Erasmus Medical Centre Department of General Pediatrics, University Medical Center<br>Jaddoe, Vincent; Erasmus Medical Center, The Generation R Study Group, University Medical Center; Erasmus Medical Centre Department of General Pediatrics, University Medical Center<br>Welten, Marieke; Erasmus Medical Center, The Generation R Study Group, Erasmus Medical Center; Erasmus Medical Center, Department of Pediatrics

Spada, Elena; Meyer Children's University Hospital, Unit of Epidemiology
REBAGLIATO, MARISA; Universitat Jaume I, Predepartamental Unit of Medicine; CIBERESP
Beneito, Andrea; FISABIO, Joint Research Unit in Epidemiology, Environment and Health
Ronfani, Luca; Istituto di Ricovero e Cura a Carattere Scientifico materno infantile Burlo Garofolo, Clinical Epidemiology and Public Health Research Unit
Charles, Marie-Aline; Inserm and INED joint research group; Inserm (U1018), CESP

Keywords: EPIDEMIOLOGY, Health policy < HEALTH SERVICES ADMINISTRATION & MANAGEMENT, Community child health < PAEDIATRICS, PUBLIC HEALTH, STATISTICS & RESEARCH METHODS

SCHOLARONE™
Manuscripts

I, the Submitting Author has the right to grant and does grant on behalf of all authors of the Work (as defined in the below author licence), an exclusive licence and/or a non-exclusive licence for contributions from authors who are: i) UK Crown employees; ii) where BMJ has agreed a CC-BY licence shall apply, and/or iii) in accordance with the terms applicable for US Federal Government officers or employees acting as part of their official duties; on a worldwide, perpetual, irrevocable, royalty-free basis to BMJ Publishing Group Ltd ("BMJ") its licensees and where the relevant Journal is co-owned by BMJ to the co-owners of the Journal, to publish the Work in this journal and any other BMJ products and to exploit all rights, as set out in our licence.

The Submitting Author accepts and understands that any supply made under these terms is made by BMJ to the Submitting Author unless you are acting as an employee on behalf of your employer or a postgraduate student of an affiliated institution which is paying any applicable article publishing charge ("APC") for Open Access articles. Where the Submitting Author wishes to make the Work available on an Open Access basis (and intends to pay the relevant APC), the terms of reuse of such Open Access shall be governed by a Creative Commons licence – details of these licences and which Creative Commons licence will apply to this Work are set out in our licence referred to above.

Other than as permitted in any relevant BMJ Author's Self Archiving Policies, I confirm this Work has not been accepted for publication elsewhere, is not being considered for publication elsewhere and does not duplicate material already published. I confirm all authors consent to publication of this Work and authorise the granting of this licence.

For peer review only

Children of migrants' birthweight in Europe and Australia: A LifeCycle comparative study

Authors

Sandra Florian[1], Mathieu Ichou[2], Lidia Panico[2], Stéphanie Pinel-Jacquemin[2], Tanja G.M. Vrijkotte[3], W.M. Harskamp-van Ginkel[3], Rae-Chi Huang[4], Jennie Carson[4,] Loreto Santa Marina[5,6,7], Mikel Subiza-Pérez[6,7,8], Martine Vrijheid[7,9,10], Sílvia Fernández-Barrés[7,9,10,] Tiffany C. Yang[11], Eva Corpeleijn[12], Marloes Cardol[12], Elena Isaevska[13], Chiara Moccia[13], Marjolein N. Kooijman[14,15], Ellis Voerman[14,15], Vincent W.V. Jaddoe[14,15], Marieke Welten[14,15], Elena Spada[16], Marisa Rebagliato[7,17,18], Andrea Beneito[18], Luca Ronfani[19], Marie-Aline Charles[20,21]

Author affiliations

[1]Institut National de la Santé et de la Recherche Médicale (INED), 5 cours des Humanités – 93322 Aubervilliers Cedex, France, sandra.florian@inserm.fr, tel. : +33 766417640.
[2]Institut National d'Etudes Démographiques (INED), Aubervilliers Cedex, France
[3]Amsterdam UMC, University of Amsterdam, Department of Public Occupational Health, Amsterdam Public Health research institute, Meibergdreef 9, Amsterdam, Netherlands.
[4]Telethon Kids Institute, University of Western Australia
[5] Ministry of Health of the Basque Government, Sub Directorate for Public Health and Addictions of Gipuzkoa, Avenida Navarra 4, 20013 San Sebastián, Spain
[6] Biodonostia Health Research Institute, Group of Environmental Epidemiology and Child Development, Doctor Begiristain, s/n, 20014 Donostia-San Sebastian, Spain
[7]Spanish Consortium for Research on Epidemiology and Public Health (CIBERESP), Instituto de Salud Carlos III, c/ Monforte de Lemos 3-5, Madrid 28029, Spain
[8] Department of Department of Clinical and Health Psychology and Research Methods, University of the Basque Country UPV/EHU, Avenida Tolosa 70, 20018 Donostia-San Sebastián, Spain
[9] ISGlobal, Barcelona, Spain
[10] Universitat Pompeu Fabra (UPF), Barcelona, Spain
[11] Bradford Institute for Health Research, Bradford Teaching Hospitals NHS Foundation Trust, Bradford, UK
[12] Department of Epidemiology, GECKO Drenthe Cohort University of Groningen, University Medical Center Groningen, Groningen, The Netherlands
[13] Department of Medical Sciences, University of Turin, Turin, Italy
[14] The Generation R Study Group, Erasmus Medical Center, University Medical Center, PO Box 2040, 3000 CA, Rotterdam, The Netherlands.
[15] Department of Pediatrics, Erasmus Medical Center, University Medical Center, PO Box 2040, 3000 CA, Rotterdam, The Netherlands
[16] Unit of Epidemiology, Meyer Children's University Hospital, Florence, Italy
[17] Predepartamental Unit of Medicine, Universitat Jaume I, Castellón de la Plana, Spain
[18] Joint Research Unit in Epidemiology, Environment and Health, FISABIO- Universitat Jaume I-University of Valencia, Spain
[19] Clinical Epidemiology and Public Health Research Unit, Institute for Maternal and Child Health-IRCCS Burlo Garofolo, Trieste, Italy
[20] Ined, Inserm, EFS, joint unit Elfe, Aubervilliers France
[21] Université de Paris, CRESS, INSERM, INRAE, Paris, France

Key words: birth weight, child health, health inequalities, cohort studies, life course epidemiology.
Word count: 3002

For peer review only

ABSTRACT

Objective: Research on adults has identified an immigrant health advantage, known as the "immigrant heath paradox", by which migrants exhibit better health outcomes than natives. Is this health advantage transferred from parents to children in the form of higher birthweight relative to children of natives?

Setting: Western Europe and Australia.

Participants: We use data from nine birth cohorts participating in the LifeCycle Project, including five studies with large samples of immigrants' children: ELFE-France (N=18,329), the Raine study-Australia (N=2,868), BiB-UK (N=13,858), ABCD study-Netherlands (N=8,257), and the Generation R study-Netherlands (N=9,901). We include male and female babies born to immigrant and native parents.

Primary and secondary outcome measures: The primary outcome is birthweight measured in grams. Different specifications were tested: Birthweight as a continuous variable including all births (DV1), the same variable but excluding babies born with over 4500 grams (DV2), low birthweight as a 0-1 binary variable (1 = birthweight below 2500 grams) (DV3). Results using these three measures were similar, only results using DV1 are presented. Parental migration status is measured in four categories: both parents natives, both born abroad, only mother born abroad, and only father born abroad.

Results: Two patterns in children's birthweight by parental migration status emerged: higher birthweight among children of immigrants in France (+12g, p<.10) and Australia (+40g, p<.10) and lower birthweight among children of immigrants in the UK (-82g, p<.05) and the Netherlands (-80g and -73g, p<.001) compared to natives' children. Smoking during pregnancy emerged as a mechanism explaining some of the birthweight gaps between children of immigrants and natives.

Conclusion: The immigrant health advantage is not universally transferred to children in the form of higher birthweight in all host countries. Further research should investigate whether this cross-national variation is due to differences in immigrant communities, social and health care contexts across host countries.

Strengths and Limitations

- This study suggests that the immigrant health advantage is not always transferred to immigrants' children.
- Children's birthweight follows diverging patterns, higher birthweight for children of immigrants in southern European cohorts (France, Italy and Spain), and Australia; and lower birthweight for children of immigrants in central and northern European countries, including in the UK and the Netherlands.
- Immigrant mothers' lower smoking during pregnancy emerged as an important protective factor for their children birthweight.
- Our findings highlight the importance of considering the host country context for understanding inequality in health outcomes at birth.

For peer review only - http://bmjopen.bmj.com/site/about/guidelines.xhtml
For peer review only

INTRODUCTION

Birthweight is an important health indicator that has been associated with lifelong development, morbidity and mortality.[1–3] Studies have found a U-shape relationship between birthweight and the risk of developing health problems, including cardiometabolic complications, with both low and high birthweight associated with a higher risk.[4]

Research on adults has identified an immigrant health advantage, known as the "immigrant heath paradox",[4–6] by which migrants appear to have better health outcomes, including lower morbidity and mortality as well as better mental health and fewer health risk behaviors, than their native counterparts, despite being on average more socioeconomically disadvantaged.[7] Studies using American data suggest that this health advantage can be transferred from parents to children, at least during the early years, finding healthier birthweight (i.e. > 2,500g and < 4,500g) and lower probability of low birthweight among children of Hispanic immigrants in the USA[4,8,9] compared to native peers.

Yet, little research has investigated patterns in children's birthweight by parental migration status through a cross-country comparative lens, particularly in Europe.[10] Investigating inequality in children's birthweight according to parents' migration status is important given its potential to inform social and health policies. Differences in national origin and host country contexts (social policies, health systems, economic opportunities, etc.) are likely to shape inequalities in children's birth outcomes as well as immigrant parents' abilities to invest in their children's health and transfer them health "capital".[2] Thus, we expect that children' birthweight by parental migrant status will vary across host countries.

Parental socioeconomic status is also an important determinant of children's health outcomes,[7,11,12] and, hence, a potential confounder of the relationship between migration status and

For peer review only - http://bmjopen.bmj.com/site/about/guidelines.xhtml

birthweight. Children of less educated parents and with low family incomes are more likely to exhibit lower birthweight.[6,13,14] Although immigrants in developed countries tend to be socioeconomically disadvantaged on average, the level of this disadvantage differs across host countries. This study takes into consideration the differences in parental socioeconomic background when investigating differences in birth outcomes.

Parental health behaviors also constitute an important possible mechanism that may contribute to health inequality at birth.[15] The deleterious effects of mother's smoking during pregnancy for children's health at birth are well established in the literature.[16–19] Research indicates that immigrant mothers appear to have fewer health risk behaviors, such as less smoking and lower alcohol consumption.[18] We explore the role of maternal health behavior (smoking during pregnancy) as a potential mechanism shaping inequalities in birthweight across migrant groups.

This study focuses on the differences in birthweight between children of natives and children of immigrants using recent data from eight European countries and Australia to investigate three questions. First, do children of immigrants exhibit a higher or lower birthweight relative to children of natives? Second, how do these associations vary across host countries? Third, can maternal smoking during pregnancy explain any of these differences?

METHODS

Data

Data come from the Horizon 2020-funded LifeCycle Project-EU Child Cohort Network,[20] which comprises nineteen pregnancy and childhood cohorts in Europe and Australia investigating early life health and its determinants, starting from pregnancy. LifeCycle cohorts collected data on demographic and socio-economic characteristics, including maternal health, marital status, living

For peer review only - http://bmjopen.bmj.com/site/about/guidelines.xhtml

arrangements, parental education, family income, and parental health behaviors, amongst other variables.[20]

A total of nine cohorts collected data on migration status and were able to harmonize variables on socioeconomic and migrant status associated with health at birth. The results presented in the main text are based on five cohorts with a sufficiently large sample size (> 1,700 children of immigrants; see Table 1) to conduct multivariate analyses. These cohorts include: the Etude Longitudinale Française depuis l'Enfance ELFE (France), a nationally representative cohort study following 18,329 births in France to mothers aged 18 and over in 2011;[21] the Raine study (Australia), a prospective study of 2,868 babies collecting data starting in 1989 from pregnancy onwards;[22] the Born in Bradford BiB study (UK) that includes data from 13,858 children born during 2007–2011 in Bradford;[23] the Amsterdam Born Children and their Development ABCD study (Netherlands) including data from 8,257 pregnancies in Amsterdam between 2003 and 2004;[24] and the Generation R study (Netherlands) which enrolled 9,153 mothers with a delivery date from April 2002 until January 2006, with a total of 9,747 live born children.[25] Table 1 presents the sample distribution of parental migration status and maternal region of birth for these five cohorts.

The other participating cohorts with smaller sample sizes (< 1,700 children of immigrants) included the NINFEA study (Italy),[26] the Piccolipiù study (Italy),[27] the INfancia y Medio Ambiente (Environment and Childhood) INMA study (Spain)[28] and the GECKO Drenthe study (Netherlands).[29] Because of smaller sample sizes, it is more difficult to draw conclusions from these cohorts, nevertheless, Appendix 1 presents the sample distribution of parental migration status and maternal region of birth for participating cohorts with a small number of children of immigrants. Model results for these smaller-sample cohorts are presented in Appendix 2.

For peer review only - http://bmjopen.bmj.com/site/about/guidelines.xhtml

Table 1. Cohort sample distribution of children's migration status and mother's region of origin (large cohorts)

	Raine (AU)		ELFE (FR)		BiB (UK)		ABCD (NL)		Gen R (NL)	
	Freq.	%	Freq.	%	Freq.	%	Freq.	%	Freq.	%
**Children's migration status**										
2nd Generation	797	27.8	1241	6.8	2564	18.5	2232	27	1887	19.4
2.5 Gen. (immigrant mother)	419	14.6	831	4.5	1621	11.7	771	9.3	767	7.9
2.5 Gen. (immigrant father)	485	16.9	1030	5.6	1869	13.5	960	11.6	1040	10.7
Natives	1167	40.7	12107	66.1	5361	38.7	4021	48.7	4588	47.1
Missing	0	0	3120	17	2443	17.6	273	3.3	1465	15.0
**Total**	**2868**	**100**	**18329**	**100**	**13858**	**100**	**8257**	**100**	**9747**	**100.0**
**Mother's region of birth**										
Host country-born	1652	57.6	13589	74.1	7263	52.4	5081	61.5	5929	60.8
Western EU/EEA	643	22.4	278	1.5	95	0.7	443	5.4	436	4.5
Eastern EU	34	1.2	101	0.6	256	1.9	76	0.9	56	0.6
Other Europe & Central Asia	0	0	140	0.8	28	0.2	406	4.9	587	6.0
East Asia & Pacific	149	5.2	129	0.7	124	0.9	161	2	200	2.1
South Asia	211	7.4	23	0.1	3347	24.1	152	1.8	31	0.3
Middle East & North Africa	15	0.5	858	4.7	83	0.6	746	9	533	5.5
Sub-Saharan Africa	64	2.2	604	3.3	238	1.7	375	4.5	363	3.7
Latin America & Caribbean	16	0.6	125	0.7	14	0.1	690	8.4	884	9.1
North America	12	0.4	18	0.1	15	0.1	73	0.9	39	0.4
Missing	72	2.5	2464	13.4	2395	17.3	54	0.7	689	7.1
**Total**	**2868**	**100**	**18329**	**100**	**13858**	**100**	**8257**	**100**	**9747**	**100.0**

Measures

The dependent variable is the child's birthweight, a continuous variable measured in grams. Different specifications of our dependent variable are tested (see Data analysis section below). Children's migration status is measured in four categories: second generation children, with both parents born abroad; 2.5 generation children with a mother born abroad, and native-born father; 2.5 generation children with a father born abroad and a native-born mother; and natives (reference), which included children with both parents born in the host country. Mothers' region of birth was measured in ten categories: host country (reference for each cohort study), Western Europe, Eastern Europe, Other Europe and Central Asia, East Asia and Pacific, South Asia, Middle East and North Africa, Sub-Saharan Africa, Latin America and the Caribbean, and North America.

For peer review only - http://bmjopen.bmj.com/site/about/guidelines.xhtml

Child characteristics at birth that were used as control variables included: the child's sex; a binary indicator for multiple birth (single birth, multiple birth); birth order measured with a continuous variables indicating the mother's parity; and the child's gestational age (in days).

Basic controls for mother's characteristics included maternal height (cm) and pre-pregnancy weight (kg), measured as continuous variables.

We consider two indicators of socioeconomic background: mother's education and household income at birth. Mother's level of education was measured in three categories: high (reference), medium, and low education. Household income quintile was measured using a cohort-specific log of the equivalized total disposable household monthly income in 2011, predicted using pan-European Union Statistics on Income and Living Conditions (EUSILC) data.[30] The Australian Raine Study and the Dutch ABCD cohort used a household income quartile measure (the fourth quartile being the richest), collected at age 1 and 5 respectively.

Finally, we included a binary indicator of mother's smoking behavior during pregnancy (0 = no smoking during pregnancy, 1 = any smoking during the pregnancy). Each of the participating cohorts conducted the variable harmonization for all the variables, following strict step-by-step protocols (see https://pubmed.ncbi.nlm.nih.gov/33884544/). Quality control checks were conducted to ensure the correct harmonization of variables across cohorts.

Data analysis

After descriptive statistics analyses, we selected our analytical sample following a complete-case analysis approach. We used ordinary least square (OLS) and logistic regressions to model different specifications of birthweight. First, we modelled birthweight as a continuous variable, and include all births (DV1). Second, we excluded babies born with a birthweight higher than 4500 grams (DV2) given their higher risk of negative health outcomes. Finally, we modelled low birthweight

For peer review only - http://bmjopen.bmj.com/site/about/guidelines.xhtml

as a 0-1 binary variable where 1 indicates birthweight below 2500 grams (DV3). As the results for these three models were very similar, only DV1 is presented in the results section. Models using the other specifications are available as supplementary material (Appendix 3 & 4).

Analyses were conducted separately for each cohort. Each cohort conducted two sets of models for all three birthweight specifications. The first set (Table 2) predicted birthweight regressed on children's migration status, while the second set (Table 3) regressed birthweight on maternal region of birth. Each set of models included three nested models. Model 1 (M1) controlled for basic characteristics at birth, including the child's sex, birth order, gestational age, and the mother's height and pre-pregnancy weight. Model 2 (M2) additionally controls for family socioeconomic characteristics, including the mothers' education and family income. Model 3 (M3) further adjusted for mothers' smoking during pregnancy.

RESULTS

Birthweight differences by parental migration status

We observed two distinct patterns regarding the disparities of children's weight at birth by parental migration status in the five largest cohorts (Table 2 & Figures 1 and 2). The "small sample" cohorts exhibited similar patterns, but differences were not statistically significant, possibly due to small samples of children of immigrants (Appendix 2).

In the first pattern, depicted by two cohorts, ELFE (France) and Raine (Australia), children of immigrants exhibited on average higher weight at birth relative to children of natives, as shown in Figure 1. For ELFE, Model M1 (gray bars) shows a slight tendency for children of immigrants to be born with higher weight than children with both native parents, only among 2.5 generation (mother, +48g, $p<.001$). For the ELFE cohort, adjusting for socioeconomic characteristics in

For peer review only - http://bmjopen.bmj.com/site/about/guidelines.xhtml

Table 2: OLS regression coefficients of children's migration status on child's birthweight (large cohorts)

DV1: Birthweight	ELFE (FR) M1 Child controls		M2 SES		M3 Smoked		RAINE STUDY (AU) M1 Child controls		M2 SES		M3 Smoked	
**Children's migration status**												
Natives (ref.)												
2nd generation	11.9		42.5	*	13.0		39.8	+	34.4		16.7	
2.5 generation (mother)	47.6	**	53.9	***	43.2	**	41.4		38.0		32.6	
2.5 generation (father)	3.2		16.7		11.6		48.1	+	46.7	+	38.2	
**Child controls**												
Female	-149.3	***	-149.2	***	-150.2	***	-130.2	***	-129.8	***	-123.3	***
Plural birth[a]	-372.1	***	-374.2	***	-378.0	***	0.0		0.0		0.0	
Mother's parity (birth order)	49.9	***	54.9	***	54.0	***	64.8	***	65.3	***	64.7	***
Gestational age	23.7	***	23.6	***	23.5	***	20.0	***	20.0	***	20.1	***
**Mother controls**												
Height	9.0	***	8.5	***	8.7	***	11.0	***	10.7	***	11.0	***
Pre-pregnancy weight	4.7	***	5.0	***	4.9	***	5.6	***	5.7	***	5.5	***
**SES**												
**Education[b]**												
High (ref.)												
Medium			-11.0		-2.7				16.4		18.5	
Low			-34.8	*	-15.9				-1.0		19.5	
**Household income quintiles[c]**												
1st quintiles (ref.)												
2nd quintile			52.3	***	44.9	***			-6.2		4.9	
3th quintile			50.2	***	32.9	*			-31.4		-13.7	
4th quintile			57.3	***	36.9	*			-30.6		2.3	
5th quintile			60.8	***	36.4	*			-		-	
**Mother smoked (pregnancy)**					-118.6	***					-151.6	***
**Constant**	**-5049.5**	***	**-4996.3**	***	**-4972.1**	***	**-4281.4**	***	**-4236.2**	***	**-4261.9**	***
N	12494		12494		12494		2283		2283		2283	
R^2	0.358		0.361		0.369		0.379		0.38		0.393	

*†p < .10,*p < .05, **p < .01, ***p < .001*

[a]The ABCD & RAINE STUDY cohorts only included single births in their sample.

[b]Level of education based on the highest on-going or completed education when the child was 0 year old (between >-1 year and <1 year). If more th one education level is reported within the defined time frame, we used highest recorded education level.

Classification according to International Standard Classification of Education 97/2011 (ISCED-97/2011)

High: Short cycle tertiary, Bachelor, Masters, Doctoral or equivalent (ISCED-2011: 5-8, ISCED-97: 5-6)

Medium: Upper secondary, Post-secondary non-tertiary (ISCED-2011: 3-4, ISCED-97: 3-4)

Low: No education; early childhood; pre-primary; primary; lower secondary or second stage of basic education. (ISCED-2011: 0-2, ISCED-97: 0-2)

[c]Eusilc income quintiles for all cohorts except for RAINE (AU) and ABCD (NL) that measured household income in quartiles.

For peer review only - http://bmjopen.bmj.com/site/about/guidelines.xhtml

M1: Basic child et mother's controls at birth

M2: M1 + socioeconomic variables
M3: M2 + mother’s smoking during pregnancy

BIB (UK)			ABCD (NL)			GEN R (NL)		
**M1**	**M2**	**M3**	**M1**	**M2**	**M3**	**M1**	**M2**	**M3**
**Child controls**	**SES**	**Smoked**	**Child controls**	**SES**	**Smoked**	**Child controls**	**SES**	**Smoked**
-81.5 ***	-83.4 ***	-118.8 ***	-79.9 ***	-52.6 *	-66.5 **	-73.4 ***	-33.6 +	-50.2 *
-97.8 ***	-94.7 ***	-132.8 ***	15.0	23.6	15.1	26.7	42.5 +	36.4
-117.0 ***	-120.8 ***	-147.8 ***	-78.0 ***	-65.3 **	-61.3 **	-75.3 ***	-53.9 **	-51.1 **
-145.4 ***	-143.5 ***	-144.6 ***	-121.7 ***	-123.2 ***	-123.4 ***	-106.8 ***	-107.2 ***	-108.2 ***
-311.2 ***	-305.0 ***	-309.9 ***	0.0	0.0	0.0	-404.8 ***	-408.5 ***	-409.1 ***
54.8 ***	62.7 ***	59.8 ***	82.2 ***	84.4 ***	83.3 ***	109.3 ***	111.9 ***	109.3 ***
25.3 ***	25.3 ***	25.2 ***	25.7 ***	25.6 ***	25.6 ***	26.1 ***	26.0 ***	25.9 ***
11.3 ***	10.9 ***	11.1 ***	10.4 ***	9.5 ***	9.5 ***	10.1 ***	9.2 ***	9.3 ***
5.6 ***	5.6 ***	5.6 ***	4.6 ***	5.0 ***	4.9 ***	4.5 ***	4.8 ***	4.8 ***
	-0.7	1.7		-54.6 ***	-43.8 **		-10.0	-7.3
	-24.9	-21.9		-59.6 **	-38.1 +		-30.7	-23.0
	1.7	-9.7		3.2	6.3		21.4	15.2
	-38.0 .	-22.9		-3.4	6.5		56.1 *	42.9 +
	-38.8 .	-27.7		-30.0	-17.6		77.1 *	56.4 +
	-68.6 **	-31.0		-	-		84.2 *	63.1 +
		-135.7 ***			-135.4 ***			-79.5 ***
**-5889.6** ***	**-5810.7** ***	**-5766.4** ***	**-5729.2** ***	**-5570.0** ***	**-5547.3** ***	**-5951.2** ***	**-5823.6** ***	**-5780.1** ***
4132	4132	4132	4,032	4,032	4,032	4877	4877	4877
0.489	0.492	0.498	0.4	0.403	0.408	0.46	0.46	0.46

For peer review only - http://bmjopen.bmj.com/site/about/guidelines.xhtml

Model M2 (white bars), children of immigrants' weight *increased* compared to natives and was significant for the 2nd generation (+43g, $p<.05$) and 2.5 generation mother (+54g, $p<.001$). By contrast, for the Raine Study, controlling for socioeconomic factors somewhat *decreased* children of immigrants' birthweight relative to children of natives, most of the differences became non-statistically significant. Model M3, which additionally controlled for mothers' smoking during pregnancy (black bars), showed a reduction in the magnitude of the coefficients for children of immigrants for both cohorts (still significant and positive for ELFE's 2.5 generation (mother), +43g, $p<.01$).

[Insert Fig. 1 about here]

The opposite pattern is observed in Figure 2, where children of immigrants exhibit lower birthweights compared to children of natives. This pattern characterizes the BiB (Bradford, UK), the ABCD (Amsterdam, Netherlands), and the Generation R (Rotterdam, Netherlands) cohorts. Exceptions to this pattern are 2.5 generation children with an immigrant mother in the two Dutch cohorts, where no significant differences from natives' birthweight could be detected.

For the Dutch cohorts (ABCD and Generation R), the differences in birthweight for children with two immigrant parents and for those with one immigrant father are somewhat reduced after controlling for socioeconomic characteristics (Model M2), suggesting that the lower birthweight might be due to immigrants' disadvantaged socioeconomic position within the host country. By contrast socioeconomic factors do not seem to play an important role in explaining birthweight differences for the BiB (UK) cohort[31].

[Insert Fig. 2 about here]

For peer review only - http://bmjopen.bmj.com/site/about/guidelines.xhtml

As in the first group, mothers' smoking during pregnancy had a negative effect on children's birthweight. Despite mothers' lower prevalence of smoking during pregnancy, children of immigrants' still exhibit lower birthweight than children of natives after controlling for this variable. As shown in Figure 2, for BiB birthweight gaps tend to increase in magnitude after controlling for smoking during pregnancy (Model M3, 2nd generation -119g, p<.001, 2.5 gen-mother -133gr, p<.001 and 2.5 gen-father -148gr, p<.001), indicating that if immigrant mothers smoked as much as native mothers, children of immigrants' birthweight would be even lower. For ABCD and GEN R, smoking has a smaller effect, but it slightly increased the gap for the second generation (-66g, p<.01 for ABCD, and -50g, p<.05 for GEN R).

In sum, we find higher birthweight for children of immigrants in our southern European studies, such as France and the smaller cohorts representing Italy and Spain, as well as Australia; and lower birthweight for children of immigrants in central and northern European countries, such as the UK and the Netherlands. These patterns could result from differences in the immigrants' regions of origin across host countries. We therefore analyze birthweight differences by maternal region of origin in the next section.

Birthweight differences by maternal region of birth

Results from the models by mothers' region of origin are presented in Table 3. First, we observe heterogeneity in birthweight by immigrant mothers' regions of birth. For ELFE (France), the pattern of birthweight "advantage" and the changes across models are mostly driven by children of Middle East and North African immigrants, the largest immigrant group in France, as well as by children of East Asia & Pacific immigrants. However, children of Sub-Saharan Africans, the second largest immigrant group in France, as well as of Latin Americans & Caribbean and North Americans, do not appear to have statistically significantly different birthweights relative to

For peer review only - http://bmjopen.bmj.com/site/about/guidelines.xhtml

children of natives. Nonetheless, immigrant mothers exhibit lower smoking rates in most cohorts; thus, controlling for mother's smoking during pregnancy (Table 3) explained part of the weight "advantage" for two groups (East Asia & Pacific: +132g, $p<.01$; Middle East & North Africa: +61g, $p<.01$).

In the Raine study, children of Western Europeans, the largest immigrant group, showed slightly higher birthweight than natives, but differences were not statistically significant. Children of North American mothers also reported heavier weights at birth (M1: +374g, $p<.01$; M2: +373g, $p<.01$, M3: + 355g, $p<.01$), but these estimates are based on small sample sizes. Children with a South Asian and Sub-Saharan's background tended to have lower and less distinct birthweight pattern than children of natives, but the difference was again not statistically significant.

For BiB, the observed pattern is driven by children of South Asian immigrants, the largest immigrant group in Bradford, who weighed less at birth than children of natives (M1: -74g, $p<.001$, M2: -69g, $p<.001$, M3: -94g, $p<.001$). By contrast, children of European and East Asia and Pacific as well as North American immigrants had a higher non-significant birthweight than children of natives. The weight disadvantage among children of South Asian immigrants was partly explained by socioeconomic status. Adjusting for mother's smoking during pregnancy exacerbated their "disadvantage".

For ABCD, children of women from South Asia and Latin America & Caribbean exhibited significantly lower birthweight, even after controls. Children of East Asian mothers had a significantly higher birthweight after controlling for socioeconomic variables and mother's smoking.

For peer review only - http://bmjopen.bmj.com/site/about/guidelines.xhtml

Table 3. OLS regression coefficients of mother's region of origin on child's birthweight (large cohorts)

DV: Birthweight	ELFE (FR) M1 Child controls	ELFE (FR) M2 SES	ELFE (FR) M3 Smoked	RAINE STUDY (AU) M1 Child controls	RAINE STUDY (AU) M2 SES	RAINE STUDY (AU) M3 Smoked
**Mother's region of origin**						
Host country-born (ref.)						
Western EU/EEA	24.7	21.0	20.8	37.2	30.3	25.3
Eastern EU	97.9 +	94.6 +	85.2 +	38.1	37.5	29.9
Other Europe & Central Asia	5.7	13.8	1.0			
East Asia & Pacific	145.3 **	142.3 **	131.8 **	13.8	10.1	-9.0
South Asia	211.9	211.1	244.7	-51.2	-54.2	-87.4
Middle East & North Africa	60.4 **	86.6 ***	61.2 **	40.2	40.8	36.5
Sub-Saharan Africa	-38.5 +	-9.8	-40.1 +	-35.8	-46.9	-67.9
Latin America & Caribbean	-7.1	8.7	-14.5	165.7	165.7	126.6
North America	-67.6	-76.6	-56.7	374.4 **	373.1 **	354.9 **
**Child controls**						
Female	-149.5 ***	-149.2 ***	-150.3 ***	-128.2 ***	-128.3 ***	-122.4 ***
Plural birth[a]	-372.7 ***	-374.2 ***	-378.6 ***	0.0	0.0	0.0
Mother's parity (birth order)	50.3 ***	55.3 ***	54.2 ***	64.7 ***	65.1 ***	64.0 ***
Gestational age	23.7 ***	23.6 ***	23.5 ***	20.1 ***	20.2 ***	20.2 ***
**Mother controls**						
Height	9.1 ***	8.5 ***	8.8 ***	10.6 ***	10.3 ***	10.6 ***
Pre-pregnancy weight	4.7 ***	5.0 ***	4.9 ***	5.7 ***	5.8 ***	5.6 ***
**SES**						
**Education[b]**						
High (ref)						
Medium		-11.8	-3.2		12.6	13.7
Low		-34.4 +	-15.1		-6.5	13.7
**Household income quintiles[c]**						
1st quintile (ref.)						
2nd quintile		51.2 ***	43.8 ***		-4.8	7.6
3th quintile		49.6 ***	32.2 *		-35.3	-15.1
4th quintile		55.1 ***	34.4 *		-37.1	2.3
5th quintile		58.4 ***	33.9 *			
**Mother smoked (pregnancy)**			-119.8 ***			-157.5 ***
**Constant**	**-5042.5** ***	**-4992.3** ***	**-4965.3** ***	**-4264.0** ***	**-4212.1** ***	**-4220.3** ***
N	**12419**	**12419**	**12419**	**2310**	**2310**	**2310**
**R^2**	0.358	0.36	0.369	0.38	0.38	0.4

*†p < .10,*p < .05, **p < .01, ***p < .001.*

[a]The ABCD & RAINE STUDY cohorts only included single births in their sample.

[b]Level of education based on the highest on-going or completed education when the child was born (between >-1 year and <1 year). If more than one educational level is reported within the defined time frame, we used highest recorded educational level.

Classification according to International Standard Classification of Education 97/2011 (ISCED-97/2011)
High: Short cycle tertiary, Bachelor, Masters, Doctoral or equivalent (ISCED-2011: 5-8, ISCED-97: 5-6)
Medium: Upper secondary, Post-secondary non-tertiary (ISCED-2011: 3-4, ISCED-97: 3-4)
Low: No education; early childhood; pre-primary; primary; lower secondary or second stage of basic education. (ISCED-2011: 0-2, ISCED-97: 0-2)
[c]*Eusilc income quintiles for all cohorts except for RAINE (AU) and ABCD (NL) that measured household income in quartiles.*
M1: Basic child et mother's controls at birth
M2: M1 + socioeconomic variables
M3: M2 + mother's smoking during pregnancy

For peer review only

For peer review only - http://bmjopen.bmj.com/site/about/guidelines.xhtml

BIB (UK)						ABCD (NL)						GEN R (NL)					
M1 Child controls		M2 SES		M3 Smoked		M1 Child controls		M2 SES		M3 Smoked		M1 Child controls		M2 SES		M3 Smoked	
45.1		42.9		30.3		8.1		10.8		1.1		32.3		40.5		34.3	
44.1		34.6		29.5		-99.2		-83.2		-90.0		68.3		99.1		78.7	
-10.2		6.5		-13.3		29.0		67.7	+	68.4	+	11.5		58.5	*	52.2	+
115.3	.	96.1		80.2		121.5	*	134.7	*	122.9	*	124.0	**	143.3	**	127.5	**
-74.1	***	-69.4	***	-94.4	***	-174.7	**	-144.3	*	-170.9	**	11.4		35.8		9.4	
-35.3		-45.0		-60.5		-6.1		25.6		1.6		20.5		63.3	+	38.8	
-53.8		-58.1		-82.1		-54.5		-19.9		-41.0		-115.8	**	-68.5	+	-86.9	*
						-89.6	**	-63.0	*	-73.3	*	-71.5	**	-37.1		-49.8	*
108.7		86.4		70.2		-148.2	+	-142.8	+	-150.7	+	-62.5		-64.6		-74.8	
-143.1	***	-141.4	***	-141.9	***	-121.6	***	-123.2	***	-123.8	***	-107.8	***	-108.2	***	-109.1	***
-319.7	***	-313.2	***	-318.7	***	0.0		0.0		0.0		-404.5	***	-409.0	***	-409.4	***
52.8	***	59.9	***	57.0	***	80.5	***	83.6	***	82.4	***	107.1	***	111.0	***	108.7	***
25.4	***	25.5	***	25.3	***	25.7	***	25.5	***	25.5	***	26.1	***	25.9	***	25.9	***
11.9	***	11.7	***	11.9	***	10.9	***	9.7	***	9.7	***	11.4	***	10.2	***	10.2	***
5.6	***	5.6	***	5.6	***	4.7	***	5.3	***	5.2	***	4.6	***	5.0	***	4.9	***
		0.1		2.2				-66.0	***	-54.4	***			-1.9		-0.1	
		-17.4		-13.6				-78.1	***	-55.9	*			-49.7		-43.6	
		2.4		-6.1				1.0		4.4				23.9		18.0	
		-44.4	.	-33.5				-10.3		0.1				57.2	**	44.8	*
		-43.7	*	-35.7				-37.3		-23.0				93.6	**	73.5	*
		-63.5	**	-32.2										100.9	**	79.6	*
				-108.9	***					-139.9	***					-77.3	***
**-6049.7**	***	**-5989.8**	***	**-5984.4**	***	**-5822.8**	***	**-5594.3**	***	**-5568.1**	***	**-6189.1**	***	**-6004.2**	***	**-5962.2**	***
**4141**		**4141**		**4141**		**4,074**		**4,074**		**4,074**		**5123**		**5123**		**5123**	
0.485		0.488		0.492		0.398		0.402		0.408		0.456		0.461		0.465	

For peer review only - http://bmjopen.bmj.com/site/about/guidelines.xhtml

For Generation R (Rotterdam, the Netherlands), both patterns also coexist: children of women born in Sub-Saharan Africa and Latin America & Caribbean exhibited significantly lower birthweight, even after controls. Children of East Asian and of Other European & Central Asian mothers had, on the contrary, significantly higher birthweights.

DISCUSSION

Research in developed countries has identified an immigrant health advantage among adults, yet evidence on whether immigrants can transfer this health advantage to their children is mixed, and remains particularly scarce in the European context. This study investigates whether children of immigrants exhibit a health advantage on weight at birth, comparing eight European countries and Australia.

Prior studies on the role of immigration on birthweight have focused on Latin-American immigrants in the US, providing mixed evidence, for example finding lighter birthweight among Mexican and Cuban babies and heavier birthweight among Puerto Rican babies relative to US-born white women.[8] Our research extends this area of research to European countries and Australia. A first aim of this study was to evaluate whether children of immigrants exhibit a higher or lower birthweight relative to children of natives, using recent harmonized panel data of rich host countries. Two general patterns emerged: we found higher birthweight for immigrants' children in southern European and Australian studies, and lower birthweights in northern European studies.

Our second aim explored in more detail birthweight variation by mother's country of origin, controlling for the mother's height and pre-pregnancy weight. Previous studies indicate that geographic origins could be associated with children of immigrants' birthweight. For example,

For peer review only - http://bmjopen.bmj.com/site/about/guidelines.xhtml

previous work has highlighted higher birthweights for infants born in Spain to mothers of African origins and for mothers of Latin-American countries,[32] while lower mean birthweights were described for infants born in China to Asian Indian mothers.[33] One of the most interesting findings in our study is that children of South Asian mothers showed consistently lower birthweights, in both BiB (where south Asian immigrants represent the largest immigrant group), ABCD and to a lesser extent in the RAINE study. South Asia bears half of global low birthweight burden and children of South Asian parents were consistently shown to have lower birthweight than natives in UK[34], Australia and Netherlands. Another group stands out includes babies of East Asian and Pacific mothers, who were significantly heavier at birth in three cohorts, France and the two Dutch cohorts. In France, the largest immigrant group, immigrants from the Middle East and North Africa, showed the strongest advantage in birthweight, mirroring similar results previously found in Belgium.[35] Birthweights of children from Latin-American and Caribbean mothers were significantly lower in the Dutch cohorts, in contrast to findings in previous work in Spain.[32]

Our third aim was to evaluate the impact of mother's smoking during her pregnancy. Our results seem to go in the same direction as previous studies,[16,18] finding that mother's smoking during pregnancy has a negative effect on birthweight, as tobacco being a vasoconstrictor reduces placental circulation. Immigrant mother's lower prevalence of smoking during pregnancy in all study cohorts had a protective effect, which tended to favor their children's health at birth across countries.

The strength of our work lies in the use of the recent and harmonized cross-country EU Child Cohort Network data.[20] We are however not able to empirically measure other important contextual factors that might drive some of these patterns, such as access to health services, in both

For peer review only - http://bmjopen.bmj.com/site/about/guidelines.xhtml

origin and host countries, access to employment, exposure to discrimination, and to other health risk factors.

CONCLUSION

The patterns of birthweight of children of immigrants relative to natives differ across host countries. While some of this cross-country variation may be due to the diverse composition of immigrant communities across Europe and Australia, it is likely that these differences are also partly driven by variations in social and health policies in the host countries. Improving access to healthcare, especially during pregnancy, and more inclusive social policies are needed to reduce the inequalities in birthweight, especially for some immigrant groups, while encouraging positive parental health behaviors, such as lower smoking. Our results confirm the protective effects of not smoking during pregnancy for child's birthweight, highlighting the importance of supporting immigrants' healthier practices.

What is already known on this subject?
Research has identified a health advantage among immigrants relative to natives of the host country. Yet, there is a lack of evidence on whether this advantage is transferred to immigrants' children and through which mechanisms.
What this study adds?
Our findings highlight the importance of the host country context and suggest that the immigrant health advantage is not always transferred to their children. We found that children's birthweight by migrant status follow diverging patterns, with higher birthweight for children of immigrants in our southern European cohorts (France, Italy and Spain), and Australia; and lower weight at birth for children of immigrants in central and northern European studies, including in the UK and the Netherlands. Immigrant mothers' lower smoking during pregnancy was found to be an important protective factor for their children birthweight.

Research Ethics Approval

This study analyses secondary data. Each cohort study collected its own data previously and was either approved by an Ethics Committee(s) or Institutional Board(s) or exempted by them. For more information on data from each of the participating cohorts, please refer to:

For peer review only - http://bmjopen.bmj.com/site/about/guidelines.xhtml

Jaddoe VW V., Felix JF, Andersen AMN, et al. The LifeCycle Project-EU Child Cohort Network: a federated analysis infrastructure and harmonized data of more than 250,000 children and parents. Eur J Epidemiol. 2020;35(7):709-724. doi:10.1007/s10654-020-00662-z

Contributorship statement

Lidia Panico and Mathieu Ichou conceived the idea for the study. Sandra Florian and Stéphanie Pinel-Jacquemin conducted and coordinated the analyses. At least one co-author from each cohort study conducted the analysis and contributed to the interpretation of the findings. Sandra Florian drafted the manuscript with the feedback of Lidia Panico and Mathieu Ichou. All authors revised the manuscript. All authors approved the final version of the paper.

Competing interests

The authors declare no competing interests with any commercial entity or financial or non-financial associations relating to the work in the submitted manuscript.

Acknowledgments

The Elfe survey is a joint project between the French Institute for Demographic Studies (INED) and the National Institute of Health and Medical Research (INSERM), in partnership with the French blood transfusion service (Etablissement français du sang, EFS), Santé publique France, the National Institute for Statistics and Economic Studies (INSEE), the Direction générale de la santé (DGS, part of the Ministry of Health and Social Affairs), the Direction générale de la prévention des risques (DGPR, Ministry for the Environment), the Direction de la recherche, des études, de l'évaluation et des statistiques (DREES, Ministry of Health and Social Affairs), the Département des études, de la prospective et des statistiques (DEPS, Ministry of Culture), and the Caisse nationale des allocations familiales (CNAF), with the support of the Ministry of Higher Education and Research and the Institut national de la jeunesse et de l'éducation populaire (INJEP).

The ELFE cohort also acknowledges the support from F. Zariouh, M.A. Bellance, M. Cornet, and B. de Lauzon-Guillain in harmonizing the Elfe cohort data for the Life cycle project.

The authors would like to acknowledge the Raine Study participants and their families for their ongoing participation in the study and the Raine Study team for study co-ordination and data collection. We also thank the NHMRC for their long term contribution to funding the study over the last 30 years. The core management of the Raine Study is funded by The University of Western Australia, Curtin University, Telethon Kids Institute, Women and Infants Research Foundation, Edith Cowan University, Murdoch University, The University of Notre Dame Australia and the Raine Medical Research Foundation.

Born in Bradford is only possible because of the enthusiasm and commitment of the Children and Parents in BiB. We are grateful to all the participants, practitioners and researchers who have made Born in Bradford happen.

For peer review only - http://bmjopen.bmj.com/site/about/guidelines.xhtml

The general design of the Generation R Study is made possible by financial support from the Erasmus MC, University Medical Center, Rotterdam, Erasmus University Rotterdam, Netherlands Organization for Health Research and Development (ZonMw), Netherlands Organisation for Scientific Research (NWO), Ministry of Health, Welfare and Sport and Ministry of Youth and Families. This project received funding from the European Union's Horizon 2020 research and innovation programme (LIFECYCLE, grant agreement No 733206, 2016; EUCAN-Connect grant agreement No 824989; ATHLETE, grant agreement No 874583). VJ received funding from a Consolidator Grant from the European Research Council (ERC-2014-CoG-648916).

We also acknowledge The University of Western Australia, Curtin University, Women and Infants Research Foundation, Telethon Kids Institute, Edith Cowan University, Murdoch University, The University of Notre Dame Australia, and The Raine Medical Research Foundation for providing funding for the core management of the Raine Study.

INMA-Gipuzkoa is grateful to the participants of the study for their kind collaboration. The recruitment of participants and the development of the study was funded by grants from Instituto de Salud Carlos III (PI06/0867 and FIS-PI09/00090 incl. FEDER funds), Department of Health of the Basque Government (2005111093), Provincial Government of Gipuzkoa (DFG06/002), and annual agreements with the municipalities of the study area (Zumarraga, Urretxu , Legazpi, Azkoitia y Azpeitia y Beasain).

We are grateful to all the participating children, parents, practitioners and researchers who took part in the INMA Sabadell study. We acknowledge support from the Spanish Ministry of Science and Innovation through the "Centro de Excelencia Severo Ochoa 2019-2023" Program (CEX2018-000806-S), and support from the Generalitat de Catalunya through the CERCA Program.

The authors acknowledge the Piccolipiù Working Group and the families involved in the study. The Childhood Obesity Project (CHOP) thanks the participating families and all project partners for their enthusiastic support of the project. Furthermore, thanks to the European Childhood Obesity Trial Study Group, who designed and conducted the study, entered the data, and participated in the data analysis.

We are grateful to the families who took part in the GECKO Drenthe study, the midwives, gynecologists, nurses and GPs for their help for recruitment and measurement of participants, and the whole team from the GECKO Drenthe study.

Funding

The LifeCycle project received funding from the European Union's Horizon 2020 research and innovation programme (Grant Agreement No. 733206 LifeCycle).

The ELFE study received a government grant managed by the National Research Agency under the "Investissements d'avenir" programme (ANR-11-EQPX-0038).

Core funding for Born in Bradford (BiB) has been funded by the Wellcome Trust (WT101597MA), a joint grant from the UK Medical Research Council (MRC) and UK Economic and Social Science Research Council (ESRC) (MR/N024397/1); the British Heart Foundation

For peer review only - http://bmjopen.bmj.com/site/about/guidelines.xhtml

(CS/16/4/32482); and the National Institute for Health Research ARC Yorkshire and Humber (NIHR200166). The views expressed in this publication are those of the author(s) and not necessarily those of the National Institute for Health Research or the Department of Health and Social Care or Wellcome Trust.

The GECKO Drenthe birth cohort was funded by an unrestricted grant of Hutchison Whampoa Ld, Hong Kong and supported by the University of Groningen, Well Baby Clinic Foundation Icare, Noordlease, Paediatric Association Of The Netherlands, Youth Health Care Drenthe and European Union's Horizon 2020 research and innovation programme (Grant Agreement No. 733206 LifeCycle).

The Raine Study has been funded by program and project grants from the Australian National Health and Medical Research Council, the Commonwealth Scientific and Industrial Research Organisation, Healthway and the Lions Eye Institute in Western Australia. The Raine Study Gen2-17 year follow-up was funded by the NHMRC Program Grant (Stanley et al, ID 353514). The Raine Study participation in LIFECYCLE was funded by a grant from the National Health and Medical Research Council, Australia (GNT1142858). The University of Western Australia (UWA), Curtin University, the Raine Medical Research Foundation, the Telethon Kids Institute, the Women's and Infant's Research Foundation (KEMH), Murdoch University, The University of Notre Dame Australia and Edith Cowan University provide funding for the Core Management of the Raine Study.

INMA-Sabadell data collections were supported by grants from the Instituto de Salud Carlos III (Red INMA G03/176), CIBERESP, and the Generalitat de Catalunya-CIRIT (1999SGR 00241).
The NINFEA cohort was partially funded by the Compagnia San Paolo and by the Piedmont Region.

The Piccolipiù project was financially supported by the Italian National Center for Disease Prevention and Control (CCM grants years 2010 and 2014) and by the Italian Ministry of Health (art 12 and 12 bis D.lgs 502/92).

Childhood Obesity Project (CHOP): The study reported has been carried out with partial financial support from the Commission of the European Community; specific research, technological development, and demonstration program "Quality of Life and Management of Living Resources", within the European Union's Seventh Framework Programme (FP7/2007-2013); project EarlyNutrition under grant agreement no. 289346; the European Union H2020 project LIFECYCLE under grant no. 733206; the European Research Council Advanced Grant META-GROWTH (ERC-2012-AdG: no.322605) and partial financial support from the Polish Ministry of Science and Higher Education (2571/7.PR/2012/2).

Data sharing statement

This study analyses secondary data. Each cohort study collected its own data previously and was either approved by an Ethics Committee(s) or Institutional Board(s) or exempted by them. For more information on data from each of the participating cohorts, please refer to:

For peer review only - http://bmjopen.bmj.com/site/about/guidelines.xhtml

Jaddoe VW V., Felix JF, Andersen AMN, et al. The LifeCycle Project-EU Child Cohort Network: a federated analysis infrastructure and harmonized data of more than 250,000 children and parents. Eur J Epidemiol. 2020;35(7):709-724. doi:10.1007/s10654-020-00662-z

Patient and Public Involvement

No patient involved.

For peer review only

For peer review only - http://bmjopen.bmj.com/site/about/guidelines.xhtml

REFERENCES

1. Irwin LG, Siddiqi A, Hertzman C. *Early Child Development : A Powerful Equalizer. Final Report.*; 2007.
2. Kim D, Saada A. The social determinants of infant mortality and birth outcomes in western developed nations: A cross-country systematic review. *Int J Environ Res Public Health*. 2013;10(6):2296-2335. doi:10.3390/ijerph10062296
3. Gluckman PD, Hanson MA, Cooper C, Thornburg KL. Effect of In Utero and Early-Life Conditions on Adult Health and Disease. *N Engl J Med*. 2008;359(1):61-73. doi:10.1056/nejmra0708473
4. Sun D, Wang T, Heianza Y, et al. Birthweight and cardiometabolic risk patterns in multiracial children. *Int J Obes*. 2018;42(1):20-27. doi:10.1038/ijo.2017.196
5. Wallace M. Adult mortality among the descendants of immigrants in England and Wales: does a migrant mortality advantage persist beyond the first generation? *J Ethn Migr Stud*. 2016;42(9):1558-1577. doi:10.1080/1369183X.2015.1131973
6. Ichou M, Wallace M. The Healthy Immigrant Effect: The role of educational selectivity in the good health of migrants. *Demogr Res*. 2019;40:61-94. doi:10.4054/DEMRES.2019.40.4
7. Mendoza FS. Health disparities and children in immigrant families: A research agenda. *Pediatrics*. 2009;124(Suppl. 3):S187-S195. doi:10.1542/peds.2009-1100F
8. Giuntella O. The Hispanic health paradox: New evidence from longitudinal data on second and third-generation birth outcomes. *SSM - Popul Heal*. 2016;2:84-89. doi:10.1016/j.ssmph.2016.02.013
9. Wingate MS, Alexander GR. The healthy migrant theory: Variations in pregnancy outcomes among US-born migrants. *Soc Sci Med*. 2006;62(2):491-498. doi:10.1016/j.socscimed.2005.06.015
10. Sørbye IK, Vangen S, Juarez SP, et al. Birthweight of babies born to migrant mothers - What role do integration policies play? *SSM - Popul Heal*. 2019;9:100503. doi:10.1016/j.ssmph.2019.100503
11. Almond D, Currie J. Killing me softly: The fetal origins hypothesis. *J Econ Perspect*. 2011;25(3):153-172. doi:10.1257/jep.25.3.153
12. Castañeda H, Holmes SM, Madrigal DS, Young M-ED, Beyeler N, Quesada J. Immigration as a Social Determinant of Health. *Annu Rev Public Health*. 2015;36(1):375-392. doi:10.1146/annurev-publhealth-032013-182419
13. Panico L, Tô M, Thevenon O. What is the link between mothers' level of education and low birthweight? *Popul Soc*. 2015;523(1):1-4.
14. Auger N, Luo ZC, Platt RW, Daniel M. Do mother's education and foreign born status interact to influence birth outcomes? Clarifying the epidemiological paradox and the healthy migrant effect. *J Epidemiol Community Health*. 2008;62(5):402-409. doi:10.1136/jech.2007.064535
15. Wanner P, Khlat M, Bouchardy C. Health habits and behaviour of Southern European and Nort African immigrants in France. *Rev Epidemiol Sante Publique*. 1995;43(6):548-559. http://www.ncbi.nlm.nih.gov/pubmed/8552853
16. El-Khoury Lesueur F, Sutter-Dallay AL, Panico L, et al. The perinatal health of immigrant women in France: a nationally representative study. *Int J Public Health*. 2018;63(9):1027-1036. doi:10.1007/s00038-018-1146-y
17. Palloni A, Morenoff JD. Interpreting the paradoxical in the hispanic paradox. Demographic and epidemiologic approaches. *Ann N Y Acad Sci*. 2001;954:140-174. doi:10.1111/j.1749-

For peer review only - http://bmjopen.bmj.com/site/about/guidelines.xhtml

6632.2001.tb02751.x
18. Melchior M, Chollet A, Glangeaud-Freudenthal N, et al. Tobacco and alcohol use in pregnancy in France: The role of migrant status. The nationally representative ELFE study. *Addict Behav*. 2015;51:65-71. doi:10.1016/j.addbeh.2015.07.015
19. Harley K, Eskenazi B. Time in the United States, social support and health behaviors during pregnancy among women of Mexican descent. *Soc Sci Med*. 2006;62(12):3048-3061. doi:10.1016/j.socscimed.2005.11.036
20. Jaddoe VW V., Felix JF, Andersen AMN, et al. The LifeCycle Project-EU Child Cohort Network: a federated analysis infrastructure and harmonized data of more than 250,000 children and parents. *Eur J Epidemiol*. 2020;35(7):709-724. doi:10.1007/s10654-020-00662-z
21. Charles MA, Thierry X, Lanoe JL, et al. Cohort Profile: The French National cohort of children ELFE: birth to 5 years. *Int J Epidemiol*. Published online November 20, 2019. doi:10.1093/ije/dyz227
22. Newnham JP, Evans SF, Michael CA, Stanley FJ, Landau LI. Effects of frequent ultrasound during pregnancy: a randomised controlled trial. *Lancet*. 1993;342(8876):887-891. doi:10.1016/0140-6736(93)91944-H
23. Wright J, Small N, Raynor P, et al. Cohort Profile: The Born in Bradford multi-ethnic family cohort study. *Int J Epidemiol*. 2013;42(4):978-991. doi:10.1093/ije/dys112
24. van Eijsden M, Vrijkotte TGM, Gemke RJBJ, van der Wal MF. Cohort profile: the Amsterdam Born Children and their Development (ABCD) study. *Int J Epidemiol*. 2011;40(5):1176-1186. doi:10.1093/ije/dyq128
25. Kooijman MN, Kruithof CJ, van Duijn CM, et al. The Generation R Study: design and cohort update 2017. *Eur J Epidemiol*. 2016;31(12):1243-1264. doi:10.1007/s10654-016-0224-9
26. Richiardi L, Baussano I, Vizzini L, Douwes J, Pearce N, Merletti F. Feasibility of recruiting a birth cohort through the Internet: The experience of the NINFEA cohort. *Eur J Epidemiol*. 2007;22(12):831-837. doi:10.1007/s10654-007-9194-2
27. Farchi S, Forastiere F, Vecchi Brumatti L, et al. Piccolipiù, a multicenter birth cohort in Italy: protocol of the study. *BMC Pediatr*. 2014;14(1):36. doi:10.1186/1471-2431-14-36
28. Guxens M, Ballester F, Espada M, et al. Cohort Profile: the INMA--INfancia y Medio Ambiente--(Environment and Childhood) Project. *Int J Epidemiol*. 2012;41(4):930-940. doi:10.1093/ije/dyr054
29. L'Abee C, Sauer PJ, Damen M, Rake J-P, Cats H, Stolk RP. Cohort Profile: The GECKO Drenthe study, overweight programming during early childhood. *Int J Epidemiol*. 2008;37(3):486-489. doi:10.1093/ije/dym218
30. Pizzi C, Richiardi M, Charles M-A, et al. Measuring Child Socio-Economic Position in Birth Cohort Research: The Development of a Novel Standardized Household Income Indicator. *Int J Environ Res Public Health*. 2020;17(5):1700. doi:10.3390/ijerph17051700
31. Uphoff EP, Pickett KE, Wright J. Social gradients in health for Pakistani and White British women and infants in two UK birth cohorts. *Ethn Heal*. 2016;21(5):452-467. doi:10.1080/13557858.2015.1091442
32. Stanek M, Requena M, Del Rey A, García-Gómez J. Beyond the healthy immigrant paradox: decomposing differences in birthweight among immigrants in Spain. *Global Health*. 2020;16(1):87. doi:10.1186/s12992-020-00612-0
33. Hayes DK, Lukacs SL, Schoendorf KC. Heterogeneity within Asian subgroups: A comparison of birthweight between infants of US and non-us born Asian Indian and Chinese

For peer review only - http://bmjopen.bmj.com/site/about/guidelines.xhtml

mothers. *Matern Child Health J*. 2008;12(5):549-556. doi:10.1007/s10995-007-0270-8

34. Margetts BM, Yusof SM, Al Dallal Z, Jackson AA. Persistence of lower birth weight in second generation South Asian babies born in the United Kingdom. *J Epidemiol Community Health*. 2002;56(9):684-687. doi:10.1136/jech.56.9.684
35. Sow M, Schoenborn C, De Spiegelaere M, Racape J. Influence of time since naturalisation on socioeconomic status and low birth weight among immigrants in Belgium. A population-based study. *PLoS One*. 2019;14(8):e0220856. doi:10.1371/journal.pone.0220856

Figure Legend

Fig.1. OLS coefficients for cohorts in which children of immigrants exhibit higher birthweight (in gr.)

Fig. 2 OLS regression coefficients for cohorts in which children of immigrants exhibit lower birthweight (in gr.)

For peer review only

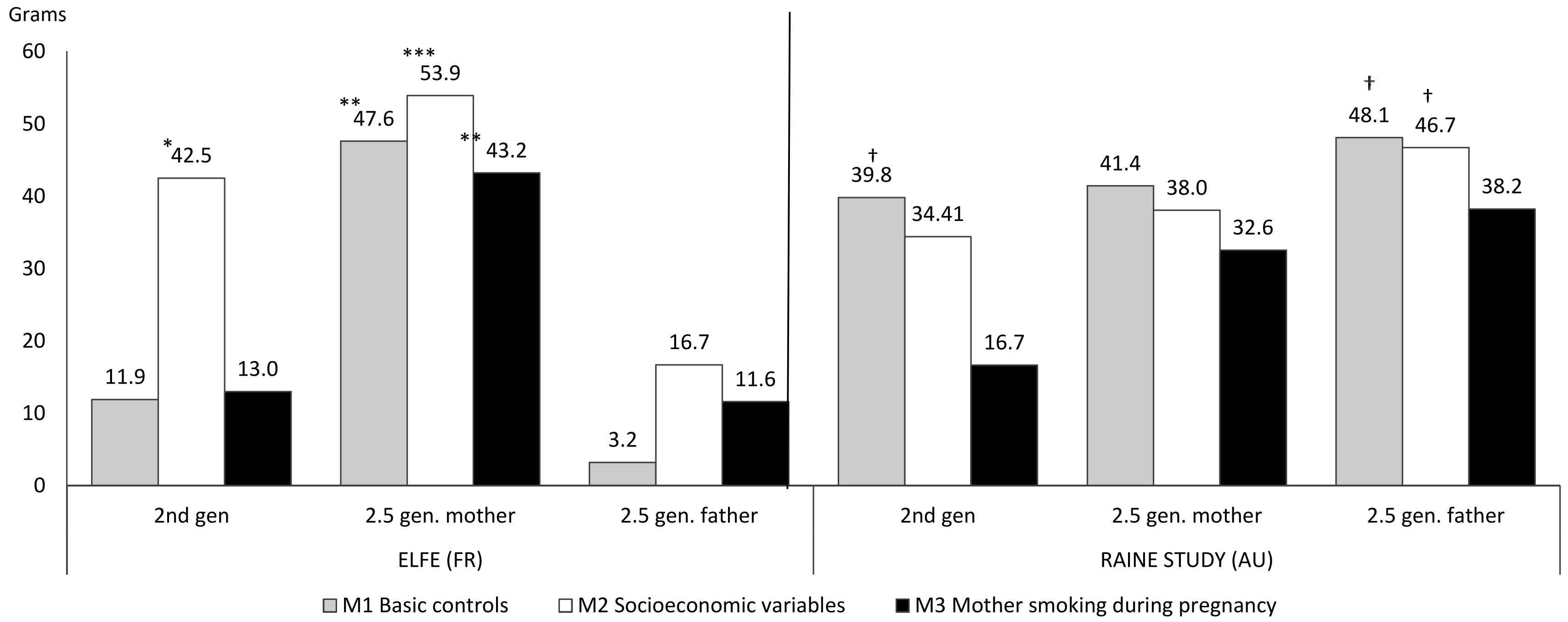

†p < .10,*p < .05, **p <.01, ***p <.001

Fig. 1. OLS coefficients for cohorts in which children of immigrants exhibit higher birthweight (in gr.)

For peer review only - http://bmjopen.bmj.com/site/about/guidelines.xhtml

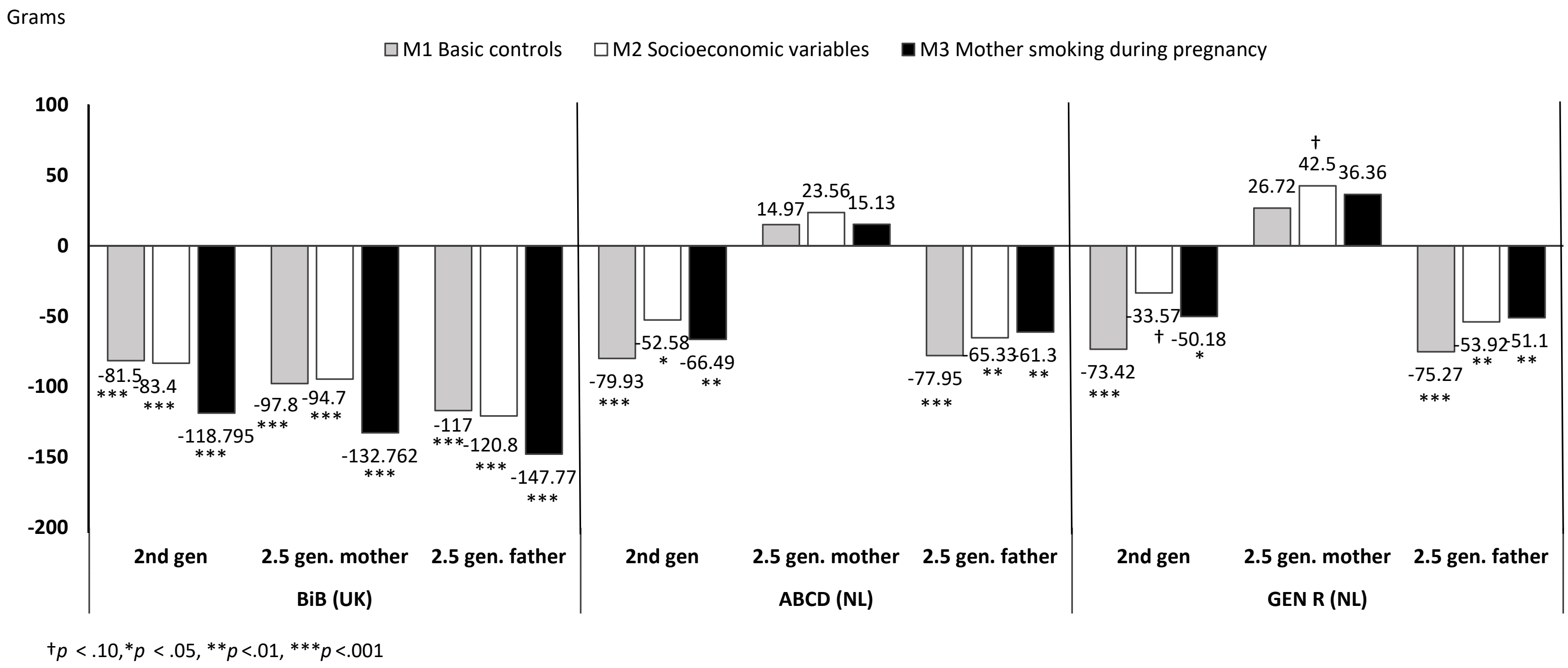

Fig. 2 OLS regression coefficients for cohorts in which children of immigrants exhibit lower birthweight (in gr.)

Table 1. Cohort sample distribution of children's migration status and mother's region of origin (large cohorts)

	Raine (AU)		ELFE (FR)		BiB (UK)		ABCD (NL)		Gen R (NL)	
	**Freq.**	**%**	**Freq.**	**%**	**Freq.**	**%**	**Freq.**	**%**	**Freq.**	**%**
**Children's migration status**										
2nd Generation	797	27.8	1241	6.8	2564	18.5	2232	27	1887	19.4
2.5 Gen. (immigrant mother)	419	14.6	831	4.5	1621	11.7	771	9.3	767	7.9
2.5 Gen. (immigrant father)	485	16.9	1030	5.6	1869	13.5	960	11.6	1040	10.7
Natives	1167	40.7	12107	66.1	5361	38.7	4021	48.7	4588	47.1
Missing	0	0	3120	17	2443	17.6	273	3.3	1465	15.0
**Total**	**2868**	**100**	**18329**	**100**	**13858**	**100**	**8257**	**100**	**9747**	**100.0**
**Mother's region of birth**										
Host country-born	1652	57.6	13589	74.1	7263	52.4	5081	61.5	5929	60.8
Western EU/EEA	643	22.4	278	1.5	95	0.7	443	5.4	436	4.5
Eastern EU	34	1.2	101	0.6	256	1.9	76	0.9	56	0.6
Other Europe & Central Asia	0	0	140	0.8	28	0.2	406	4.9	587	6.0
East Asia & Pacific	149	5.2	129	0.7	124	0.9	161	2	200	2.1
South Asia	211	7.4	23	0.1	3347	24.1	152	1.8	31	0.3
Middle East & North Africa	15	0.5	858	4.7	83	0.6	746	9	533	5.5
Sub-Saharan Africa	64	2.2	604	3.3	238	1.7	375	4.5	363	3.7
Latin America & Caribbean	16	0.6	125	0.7	14	0.1	690	8.4	884	9.1
North America	12	0.4	18	0.1	15	0.1	73	0.9	39	0.4
Missing	72	2.5	2464	13.4	2395	17.3	54	0.7	689	7.1
**Total**	**2868**	**100**	**18329**	**100**	**13858**	**100**	**8257**	**100**	**9747**	**100.0**

Table 2: OLS regression coefficients of children's migration status on child's birthweight (large cohorts)

	ELFE (FR)			RAINE STUDY (AU)			BIB (UK)			ABCD (NL)	
	M1	M2	M3	M1	M2	M3	M1	M2	M3	M1	M2
**DV1: Birthweight**	**Child controls**	**SES**	**Smoked**	**Child controls**	**SES**	**Smoked**	**Child controls**	**SES**	**Smoked**	**Child controls**	**SES**
**Children's migration status**											
Natives (ref.)											
2nd generation	11.9	42.5 *	13.0	39.8 +	34.4	16.7	-81.5 ***	-83.4 ***	-118.8 ***	-79.9 ***	-52.6 *
2.5 generation (mother)	47.6 **	53.9 ***	43.2 **	41.4	38.0	32.6	-97.8 ***	-94.7 ***	-132.8 ***	15.0	23.6
2.5 generation (father)	3.2	16.7	11.6	48.1 +	46.7 +	38.2	-117.0 ***	-120.8 ***	-147.8 ***	-78.0 ***	-65.3 **
**Child controls**											
Female	-149.3 ***	-149.2 ***	-150.2 ***	-130.2 ***	-129.8 ***	-123.3 ***	-145.4 ***	-143.5 ***	-144.6 ***	-121.7 ***	-123.2 ***
Plural birth[a]	-372.1 ***	-374.2 ***	-378.0 ***	0.0	0.0	0.0	-311.2 ***	-305.0 ***	-309.9 ***	0.0	0.0
Mother's parity (birth order)	49.9 ***	54.9 ***	54.0 ***	64.8 ***	65.3 ***	64.7 ***	54.8 ***	62.7 ***	59.8 ***	82.2 ***	84.4 ***
Gestational age	23.7 ***	23.6 ***	23.5 ***	20.0 ***	20.0 ***	20.1 ***	25.3 ***	25.3 ***	25.2 ***	25.7 ***	25.6 ***
**Mother controls**											
Height	9.0 ***	8.5 ***	8.7 ***	11.0 ***	10.7 ***	11.0 ***	11.3 ***	10.9 ***	11.1 ***	10.4 ***	9.5 ***
Pre-pregnancy weight	4.7 ***	5.0 ***	4.9 ***	5.6 ***	5.7 ***	5.5 ***	5.6 ***	5.6 ***	5.6 ***	4.6 ***	5.0 ***
**SES**											
**Education[b]**											
High (ref.)											
Medium		-11.0	-2.7		16.4	18.5		-0.7	1.7		-54.6 ***
Low		-34.8 *	-15.9		-1.0	19.5		-24.9	-21.9		-59.6 **
**Household income quintiles[c]**											
1st quintiles (ref.)											
2nd quintile		52.3 ***	44.9 ***		-6.2	4.9		1.7	-9.7		3.2
3th quintile		50.2 ***	32.9 *		-31.4	-13.7		-38.0 .	-22.9		-3.4
4th quintile		57.3 ***	36.9 *		-30.6	2.3		-38.8 .	-27.7		-30.0
5th quintile		60.8 ***	36.4 *		-	-		-68.6 **	-31.0		-
**Mother smoked (pregnancy)**			-118.6 ***			-151.6 ***			-135.7 ***		
**Constant**	**-5049.5** ***	**-4996.3** ***	**-4972.1** ***	**-4281.4** ***	**-4236.2** ***	**-4261.9** ***	**-5889.6** ***	**-5810.7** ***	**-5766.4** ***	**-5729.2** ***	**-5570.0** ***
N	12494	12494	12494	2283	2283	2283	4132	4132	4132	4,032	4,032
**R^2**	0.358	0.361	0.369	0.379	0.38	0.393	0.489	0.492	0.498	0.4	0.403

*†p < .10,*p < .05, **p < .01, ***p < .001*

[a]The ABCD & RAINE STUDY cohorts only included single births in their sample.

[b]Level of education based on the highest on-going or completed education when the child was 0 year old (between >-1 year and <1 year). If more than one education level is reported within the defined time frame, we used highest recorded ε

Classification according to International Standard Classification of Education 97/2011 (ISCED-97/2011)

High: Short cycle tertiary, Bachelor, Masters, Doctoral or equivalent (ISCED-2011: 5-8, ISCED-97: 5-6)

Medium: Upper secondary, Post-secondary non-tertiary (ISCED-2011: 3-4, ISCED-97: 3-4)

Low: No education; early childhood; pre-primary; primary; lower secondary or second stage of basic education. (ISCED-2011: 0-2, ISCED-97: 0-2)

[c]Eusilc income quintiles for all cohorts except for RAINE (AU) and ABCD (NL) that measured household income in quartiles.

M1: Basic child et mother's controls at birth

M2: M1 + socioeconomic variables

M3: M2 + mother's smoking during pregnancy

	GEN R (NL)		
**M3 Smoked**	**M1 Child**	**M2 SES**	**M3 Smoked**
-66.5 **	-73.4 ***	-33.6 +	-50.2 *
15.1	26.7	42.5 +	36.4
-61.3 **	-75.3 ***	-53.9 **	-51.1 **
-123.4 ***	-106.8 ***	-107.2 ***	-108.2 ***
0.0	-404.8 ***	-408.5 ***	-409.1 ***
83.3 ***	109.3 ***	111.9 ***	109.3 ***
25.6 ***	26.1 ***	26.0 ***	25.9 ***
9.5 ***	10.1 ***	9.2 ***	9.3 ***
4.9 ***	4.5 ***	4.8 ***	4.8 ***
-43.8 **		-10.0	-7.3
-38.1 +		-30.7	-23.0
6.3		21.4	15.2
6.5		56.1 *	42.9 +
-17.6		77.1 *	56.4 +
-		84.2 *	63.1 +
-135.4 ***			-79.5 ***
**-5547.3** ***	**-5951.2** ***	**-5823.6** ***	**-5780.1** ***
4,032	4877	4877	4877
0.408	0.46	0.46	0.46

education level.

For peer review only

Table 3. OLS regression coefficients of mother's region of origin on child's birthweight (large cohorts)

DV: Birthweight	ELFE (FR)			RAINE STUDY (AU)			BIB (UK)			ABCD (NL)			GEN R (NL)		
	M1 Child controls	M2 SES	M3 Smoked	M1 Child controls	M2 SES	M3 Smoked	M1 Child controls	M2 SES	M3 Smoked	M1 Child controls	M2 SES	M3 Smoked	M1 Child controls	M2 SES	M3 Smoked
**Mother's region of origin**															
Host country-born (ref.)															
Western EU/EEA	24.7	21.0	20.8	37.2	30.3	25.3	45.1	42.9	30.3	8.1	10.8	1.1	32.3	40.5	34.3
Eastern EU	97.9 +	94.6 +	85.2 +	38.1	37.5	29.9	44.1	34.6	29.5	-99.2	-83.2	-90.0	68.3	99.1	78.7
Other Europe & Central Asia	5.7	13.8	1.0				-10.2	6.5	-13.3	29.0	67.7 +	68.4 +	11.5	58.5 *	52.2 +
East Asia & Pacific	145.3 **	142.3 **	131.8 **	13.8	10.1	-9.0	115.3 .	96.1	80.2	121.5 *	134.7 *	122.9 *	124.0 **	143.3 **	127.5 **
South Asia	211.9	211.1	244.7	-51.2	-54.2	-87.4	-74.1 ***	-69.4 ***	-94.4 ***	-174.7 **	-144.3 *	-170.9 **	11.4	35.8	9.4
Middle East & North Africa	60.4 **	86.6 ***	61.2 **	40.2	40.8	36.5	-35.3	-45.0	-60.5	-6.1	25.6	1.6	20.5	63.3 +	38.8
Sub-Saharan Africa	-38.5 +	-9.8	-40.1 +	-35.8	-46.9	-67.9	-53.8	-58.1	-82.1	-54.5	-19.9	-41.0	-115.8 **	-68.5 +	-86.9 *
Latin America & Caribbean	-7.1	8.7	-14.5	165.7	165.7	126.6				-89.6 **	-63.0 *	-73.3 *	-71.5 **	-37.1	-49.8 *
North America	-67.6	-76.6	-56.7	374.4 **	373.1 **	354.9 **	108.7	86.4	70.2	-148.2 +	-142.8 +	-150.7 +	-62.5	-64.6	-74.8
**Child controls**															
Female	-149.5 ***	-149.2 ***	-150.3 ***	-128.2 ***	-128.3 ***	-122.4 ***	-143.1 ***	-141.4 ***	-141.9 ***	-121.6 ***	-123.2 ***	-123.8 ***	-107.8 ***	-108.2 ***	-109.1 ***
Plural birth[a]	-372.7 ***	-374.2 ***	-378.6 ***	0.0	0.0	0.0	-319.7 ***	-313.2 ***	-318.7 ***	0.0	0.0	0.0	-404.5 ***	-409.0 ***	-409.4 ***
Mother's parity (birth order)	50.3 ***	55.3 ***	54.2 ***	64.7 ***	65.1 ***	64.0 ***	52.8 ***	59.9 ***	57.0 ***	80.5 ***	83.6 ***	82.4 ***	107.1 ***	111.0 ***	108.7 ***
Gestational age	23.7 ***	23.6 ***	23.5 ***	20.1 ***	20.2 ***	20.2 ***	25.4 ***	25.5 ***	25.3 ***	25.7 ***	25.5 ***	25.5 ***	26.1 ***	25.9 ***	25.9 ***
**Mother controls**															
Height	9.1 ***	8.5 ***	8.8 ***	10.6 ***	10.3 ***	10.6 ***	11.9 ***	11.7 ***	11.9 ***	10.9 ***	9.7 ***	9.7 ***	11.4 ***	10.2 ***	10.2 ***
Pre-pregnancy weight	4.7 ***	5.0 ***	4.9 ***	5.7 ***	5.8 ***	5.6 ***	5.6 ***	5.6 ***	5.6 ***	4.7 ***	5.3 ***	5.2 ***	4.6 ***	5.0 ***	4.9 ***
**SES**															
**Education[b]**															
High (ref)															
Medium		-11.8	-3.2		12.6	13.7		0.1	2.2		-66.0 **	-54.4 **		-1.9	-0.1
Low		-34.4 +	-15.1		-6.5	13.7		-17.4	-13.6		-78.1 ***	-55.9 *		-49.7	-43.6
**Household income quintiles[c]**															
1st quintile (ref.)															
2nd quintile		51.2 ***	43.8 ***		-4.8	7.6		2.4	-6.1		1.0	4.4		23.9	18.0
3th quintile		49.6 ***	32.2 *		-35.3	-15.1		-44.4 .	-33.5		-10.3	0.1		57.2 **	44.8 *
4th quintile		55.1 ***	34.4 *		-37.1	2.3		-43.7 *	-35.7		-37.3	-23.0		93.6 **	73.5 *
5th quintile		58.4 ***	33.9 *					-63.5 **	-32.2					100.9 **	79.6 *
**Mother smoked (pregnancy)**			-119.8 ***			-157.5 ***			-108.9 ***			-139.9 ***			-77.3 ***
**Constant**	**-5042.5** ***	**-4992.3** ***	**-4965.3** ***	**-4264.0** ***	**-4212.1** ***	**-4220.3** ***	**-6049.7** ***	**-5989.8** ***	**-5984.4** ***	**-5822.8** ***	**-5594.3** ***	**-5568.1** ***	**-6189.1** ***	**-6004.2** ***	**-5962.2** ***
N	**12419**	**12419**	**12419**	**2310**	**2310**	**2310**	**4141**	**4141**	**4141**	**4,074**	**4,074**	**4,074**	**5123**	**5123**	**5123**
**R^2**	0.358	0.36	0.369	0.38	0.38	0.4	0.485	0.488	0.492	0.398	0.402	0.408	0.456	0.461	0.465

*†p < .10,*p < .05, **p < .01, ***p < .001.*

[a]The ABCD & RAINE STUDY cohorts only included single births in their sample.

[b]Level of education based on the highest on-going or completed education when the child was born (between >-1 year and <1 year). If more than one educational level is reported within the defined time frame, we used highest recorded educatior

Classification according to International Standard Classification of Education 97/2011 (ISCED-97/2011)

High: Short cycle tertiary, Bachelor, Masters, Doctoral or equivalent (ISCED-2011: 5-8, ISCED-97: 5-6)

Medium: Upper secondary, Post-secondary non-tertiary (ISCED-2011: 3-4, ISCED-97: 3-4)

Low: No education; early childhood; pre-primary; primary; lower secondary or second stage of basic education. (ISCED-2011: 0-2, ISCED-97: 0-2)

[c]Eusilc income quintiles for all cohorts except for RAINE (AU) and ABCD (NL) that measured household income in quartiles.

M1: Basic child et mother's controls at birth

M2: M1 + socioeconomic variables

M3: M2 + mother's smoking during pregnancy

Appendix 1. Sample

	GECKO (NL)		INMA (SP)		NINFEA (IT)		Piccoli Piu (IT)	
	**Freq.**	**%**	**Freq.**	**%**	**Freq.**	**%**	**Freq.**	**%**
**Children's migration status**								
1st generation	0	0	0	0	35	1	0	0
2nd generation	26	0.9	114	5	28	0.8	123	3.7
2.5 generation (mother)	60	2.1	86	3.8	110	3	151	4.5
2.5 generation (father)	64	2.3	131	5.8	95	2.6	106	3.2
natives	2445	86.0	1780	78.4	3348	91.1	2881	85.8
Missing	247	8.7	159	7	59	1.6	95	2.8
Total	**2842**	**100**	**2270**	**100**	**3675**	**100**	**3356**	**100**
**Mother's region of birth**								
Host country-born	2548	89.7	1996	87.9	3516	96.2	3031	90.3
Western EU/EEA	16	0.6	29	1.3	50	1.4	41	1.2
Eastern EU	7	0.2	23	1	32	0.9	109	3.3
Other Europe & Central Asia	11	0.4	6	0.3	12	0.3	38	1.1
East Asia & Pacific	18	0.6	3	0.1	1	0	6	0.2
South Asia	7	0.2	0	0	1	0	4	0.1
Middle East & North Africa	9	0.3	5	0.2	3	0.1	16	0.5
Sub-Saharan Africa	6	0.2	4	0.2	2	0.1	4	0.1
Latin America & Caribbean	16	0.6	161	7.1	37	1	54	1.6
North America	1	0.0	0	0	2	0.1	6	0.2
Missing	203	7.1	43	1.9	0	0	47	1.4
**Total**	**2842**	**100**	**2270**	**100**	**3656**	**100**	**3356**	**100**

For peer review only

Appendix 2 . OLS regression coefficients of mother's region of origin & child's migrant status on child's birthweight (Small cohorts - Extracts)

	GECKO (NL)			INMA (SP)			NINFEA (IT)			PICCOLOPIU (IT)		
**DV1: Birthweight**	**M1 Child controls**	**M2 SES**	**M3 Smoke**	**M1 Child controls**	**M2 SES**	**M3 Smoke**	**M1 Child controls**	**M2 SES**	**M3 Smoke**	**M1 Child controls**	**M2 SES**	**M3 Smoke**
**Child's migrant status**												
Natives (ref.)												
1st Generation	-	-	-	-	-	-	153.43 +	147.14 +	147.89 +	-	-	-
2nd Generation	-139.76	-117.46	-156.54	162.68 ***	169.51 ***	143.41 ***	118.20	109.99	108.60	118.49 ***	100.96 **	97.18 **
2.5 Generation - mother	101.21	99.87	79.96	70.50	78.87 +	75.32	41.05	40.23	39.97	56.51 +	54.19	53.27
2.5 generation - father	16.45	16.31	12.30	21.46	28.86	29.50	-70.12	-73.01 +	-74.15 +	20.68	15.51	14.58
**Constant**	**-5767.43** ***	**-5738.24** ***	**-5690.72** ***	**-4847.76** ***	**-4734.01** ***	**-4723.00** ***	**-3887.01** ***	**-3883.26** ***	**-3873.41** ***	**-4584.30** ***	**-4521.88** ***	**-4539.19** ***
**N**	**2229**	**2229**	**2229**	**1898**	**1898**	**1898**	**3216**	**3216**	**3216**	**3041**	**3041**	**3041**
**R2**	0.4023	0.4057	0.4238	0.333	0.338	0.344	0.311	0.312	0.314	0.306	0.308	0.309
**Mother's region of origin**												
Host country-born (ref.)												
Western EU/EEA	49.22	61.92	60.12	-31.75	-14.98	-22.09	-10.95	-4.73	-4.85	36.70	34.20	32.98
Eastern EU	176.27	171.70	147.02	175.44 +	182.01 +	168.67 +	124.89 *	123.63 *	126.67 *	116.58 **	103.41 **	102.47 **
Other Europe & Central Asia	255.46 +	277.51 +	260.90	-122.58	-110.36	-106.48	96.77	100.00	97.25			
East Asia & Pacific	21.84	21.53	2.13	-	-	-	-62.01	-58.73	-12.27	65.62	57.29	51.86
South Asia	113.32	148.45	88.23	-	-	-	-6.17	-10.28	-15.99			
Middle East & North Africa	1.50	34.60	-16.44	-	-	-	45.25	58.64	53.82			
Sub-Saharan Africa	58.96	83.10	39.75	-	-	-	-187.85	-191.41	-196.06			
Latin America & Caribbean	-146.75	-161.48	-196.00 +	-	-	-	80.17	79.42	76.13	96.08	87.84	88.07
North America	-	-	-	-	-	-	125.89	134.27	128.67	-	-	-
**Constant**	**-5757.77** ***	**-5737.15** ***	**-5671.80** ***	**-4892.39** ***	**-4773.55** ***	**-4763.82** ***	**-4179.89** ***	**-4185.06** ***	**-4182.61** ***	**-4526.39** ***	**-4459.02** ***	**-4477.76** ***
**N**	**2,254**	**2,254**	**2,254**	**1898**	**1898**	**1898**	**5872**	**5872**	**5872**	**3070**	**3070**	**3070**
**R2**	0.4021	0.4059	0.4238	0.336	0.34	0.346	0.408	0.409	0.41	0.304	0.306	0.307

*†p < .10,*p < .05, **p < .01, ***p < .001.*

Piccolopiù used a binary variable for parity and : Region 1: Western Europe, Region 2: Easter Europe + Other Europe & Central Asia, Region 3: Latin America & Caribbean, Region 4: other regions

M1: Basic child et mother's controls at birth

M2: M1 + socioeconomic variables

M3: M2 + mother’s smoking during pregnancy

APPENDIX 3: OLS regression coefficients of children's migration status on child's birthweight (DV2: excluded birthweight < 4,500g; large cohorts)

	ELFE (FR)			RAINE STUDY (AU)			BIB (UK)			ABCD (NL)			GEN R (NL)		
	M1b	M2b	M3b	M1b	M2b	M3b	M1b	M2b	M3b	M1b	M2b	M3b	M1b	M2b	M3b
DV2: Birthweight	Basic controls	Socioeconomic variables	Smoking habits	Basic controls	Socioeconomic variables	Smoking habits	Basic controls	Socioeconomic variables	Smoking habits	Basic controls	Socioeconomic variables	Smoking habits	Basic controls	Socioeconomic variables	Smoking habits
**Children's migration status**															
Natives (ref.)															
2nd generation	16.4	45.7 **	17.1	46.87 *	40.63 +	23.77	-80.6 ***	-81.3 ***	-115.9 ***	-84.73 ***	-58.91 **	-71.28 ***	-69.35 ***	-33.13 +	-48.09 *
2.5 generation (mother)	40.4 **	46.2 **	35.8 *	44.83 +	41.22	36.44	-99.6 ***	-95.9 ***	-133.0 ***	19.78	27.46	20.11	36.18 +	48.18 *	42.64 *
2.5 generation (father)	4.5	17.3	12.3	41.51	39.61	32.02	-108.3 ***	-112.0 ***	-138.2 ***	-73.20 ***	-61.14 **	-57.76 **	-61.37 ***	-41.06 *	-38.47 *
**Child controls**															
Female	-141.2 ***	-141 ***	-142.1 ***	-113.12 ***	-113.04 ***	-123.3 ***	-131.9 ***	-130.3 ***	-131.5 ***	-103.15 ***	-104.73 ***	-104.86 ***	-91.4 ***	-92.4 ***	-93.4 ***
Plural birth[a]	-372.8 ***	-374.7 ***	-378.4 ***	0.00	0.00	0.0	-310.6 ***	-303.6 ***	-308.5 ***	0.00	0.00	0.00	-405.8 ***	-409.5 ***	-409.9 ***
Mother's parity (birth order)	45.7 ***	50.7 ***	49.8 ***	51.40 ***	51.94 ***	64.7 ***	49.5 ***	56.9 ***	54.2 ***	72.77 ***	74.66 ***	73.80 ***	98.8 ***	102.2 ***	99.9 ***
Gestational age	23.1 ***	22.9 ***	22.9 ***	19.32 ***	19.32 ***	20.1 ***	24.9 ***	25.0 ***	24.9 ***	24.43 ***	24.35 ***	24.34 ***	24.9 ***	24.8 ***	24.7 ***
**Mother controls**															
Height	8.3 ***	7.7 ***	7.9 ***	10.86 ***	10.59 ***	10.83 ***	10.9 ***	10.6 ***	10.8 ***	8.82 ***	8.03 ***	8.02 ***	9.3 ***	8.4 ***	8.5 ***
Pre-pregnancy weight	4.3 ***	4.7 ***	4.5 ***	4.92 ***	5.00 ***	4.88 ***	4.8 ***	4.8 ***	4.8 ***	3.76 ***	4.12 ***	4.06 ***	3.9 ***	4.2 ***	4.1 ***
**SES**															
**Education[b]**															
High (ref.)															
Medium		-15.26	-7.13		12.51	13.86		2.50	4.55		-54.6 ***	-43.8 **		-11.0	-8.7
Low		-38.16 *	-19.78		-11.28	7.94		-15.80	-12.71		-59.6 **	-38.1 +		-19.9	-12.9
**Household income quintiles[c]**															
1st quintiles (ref.)															
2nd quintile		46.1 ***	39.1 ***					2.8	-7.8		5.00	7.92		27.4	22.0
3th quintile		45.5 ***	29 *		-6.15	3.76		-35.8	-21.1		-6.91	2.00		60.3 **	48.5 *
4th quintile		52.9 ***	33.3 *		-19.25	-3.87		-42.6 *	-31.6		-24.12	-12.86		85.2 **	66.5 *
5th quintile		57.3 ***	33.9 *		-23.93	6.24		-70.3 **	-34.0		-	-		79.2 *	60.2 +
**Mother smoked (pregnancy)**			-114.6 ***			-140.58 ***			-130.9 ***			-119.00 ***			-71.2 ***
**Constant**	**-4728.70** ***	**-4666.20** ***	**-4645.10** ***	**-4057.57** ***	**-4004.44** ***	**-4030.36** ***	**-5699.3** ***	**-5638.2** ***	**-5594.2** ***	**5089.76** ***	**-4940.62** ***	**4925.09** ***	**-5462.2** ***	**-5338.5** ***	**-5303.6** ***
N	**12373**	**12373**	**12373**	2242	2242	2242	4090	4090	4090	3,918	3,918	3,918	4751	4751	4751
**R^2**	0.36	0.36	0.37	0.38	0.38	0.40	0.49	0.49	0.50	0.40	0.40	0.41	0.46	0.46	0.47

*†p < .10,*p < .05, **p < .01, ***p < .001*

[a]The ABCD & RAINE STUDY cohorts only included single births in their sample.

[b]Level of education based on the highest on-going or completed education when the child was 0 year old (between >-1 year and <1 year). If more than one education level is reported within the defined time frame, we used highest recorded education level.

Classification according to International Standard Classification of Education 97/2011 (ISCED-97/2011)

High: Short cycle tertiary, Bachelor, Masters, Doctoral or equivalent (ISCED-2011: 5-8, ISCED-97: 5-6)

Medium: Upper secondary, Post-secondary non-tertiary (ISCED-2011: 3-4, ISCED-97: 3-4)

Low: No education; early childhood; pre-primary; primary; lower secondary or second stage of basic education. (ISCED-2011: 0-2, ISCED-97: 0-2)

[c]Eusilc income quintiles for all cohorts except for RAINE (AU) and ABCD (NL) that measured household income in quartiles.

M1b: Basic child et mother's controls at birth

M2b: M1b + socioeconomic variables

M3b: M2b + mother's smoking during pregnancy

APPENDIX 4: Logistic regression coefficients of children's migration status on child's birthweight (DV3: low birthweight; large cohorts)

DV3: Birthweight (ODD RATIO)	ELFE (FR)			RAINE STUDY (AU)			BIB (UK)			ABCD (NL)			GEN R (NL)		
	M1b	M2b	M3b	M1b	M2b	M3b	M1b	M2b	M3b	M1b	M2b	M3b	M1b	M2b	M3b
	Basic controls	Socioeconomic variables	Smoking habits	Basic controls	Socioeconomic variables	Smoking habits	Basic controls	Socioeconomic variables	Smoking habits	Basic controls	Socioeconomic variables	Smoking habits	Basic controls	Socioeconomic variables	Smoking habits
**Children's migration status**															
Natives (ref.)															
2nd generation	-0.38	-0.61 +	-0.35	-0.68 *	-0.60 +	-0.50	0.39	0.01	0.01	0.67 *	0.42	0.61 +	0.37	0.37	0.44
2.5 generation (mother)	-0.26	-0.35	-0.20	-0.06	0.01	0.06	0.23	0.00	0.01	-0.35	-0.39	-0.30	-0.18	-0.14	-0.16
2.5 generation (father)	-0.11	-0.18	-0.14	-0.17	-0.11	-0.05	0.85 ***	0.03 **	0.03 ***	0.43	0.25	0.28	0.58 *	0.56 *	0.53 *
**Child controls**															
Female	0.63 ***	0.63 ***	0.65 ***	0.21	0.22	0.20	-0.44 **	0.01 *	0.02 **	0.63 **	0.63 **	0.68 **	0.39 *	0.38 *	0.38 *
Plural birth[a]	1.59 ***	1.60 ***	1.66 ***	0.00	0.00	0.00	1.45 ***	0.27 ***	0.27 ***	0.00	0.00	0.00	2.05 ***	2.03 ***	2.03 ***
Mother's parity (birth order)	-0.14 *	-0.20 **	-0.21 **	-0.32 **	-0.31 **	-0.32 **	-0.14 .	-0.02 ***	-0.01 ***	-0.55 ***	-0.57 ***	-0.55 **	-0.37 **	-0.39 **	-0.37 *
Gestational age	-0.15 ***	-0.15 ***	-0.15 ***	-0.12 ***	-0.12 ***	-0.12 ***	-0.15 ***	-0.01 ***	-0.01 ***	-0.16 ***	-0.16 ***	-0.16 ***	-0.16 ***	-0.16 ***	-0.16 ***
**Mother controls**															
Height	-0.03 **	-0.02 *	-0.02 *	-0.06 **	-0.06 **	-0.06 **	-0.06 ***	0.00 ***	0.00 ***	-0.04 *	-0.03 +	-0.04 +	-0.05 ***	-0.04 **	-0.05 **
Pre-pregnancy weight	-0.02 ***	-0.02 ***	-0.02 ***	0.00	0.00	0.00	-0.02 **	0.00 *	0.00 *	-0.01	-0.01	-0.01	-0.02 *	-0.02 *	-0.02 *
**SES**															
**Education**[b]															
High (ref.)															
Medium		0.16	0.11		0.09	0.07		-0.01	-0.01		0.32	0.13		-0.26	-0.26
Low		0.50 *	0.38		0.36	0.25		0.00	0.00		0.18	-0.12		-1.16 *	-1.19 *
**Household income quintiles**[c]															
1st quintiles (ref.)															
2nd quintile		-0.25	-0.20					-0.01	-0.01		0.12	0.10		-0.07	-0.05
3th quintile		-0.24	-0.10		0.02	-0.07		0.01	0.01		0.68 *	0.60 +		-0.33	-0.27
4th quintile		-0.31	-0.11		-0.31	-0.43		0.01	0.01		0.48	0.41		-1.07 *	-0.96 *
5th quintile		-0.53 *	-0.32		-0.03	-0.26		0.01	0.00					-0.34	-0.23
**Mother smoked (pregnancy)**			0.82 ***			0.72 **			0.01			1.28 ***			0.31
**Constant**	**41.97** ***	**41.23** ***	**41.62** ***	**37.64** ***	**37.37** ***	**37.77** ***	**49.40** ***	**3.53** ***	**3.53** ***	**45.15** ***	**44.16** ***	**45.66** ***	**48.74** ***	**48.80** ***	**48.87** ***
N	12494	12494	12494	2283	2283	2283	4132	4132	4132	4,032	4,032	4,032	4877	4877	4877
**ll**	-1294	-1283.8	-1265.3	-276.92	-269.25	-263.76				-325.45	-321.52	-313.42			

*†p < .10,*p < .05, **p < .01, ***p < .001*

[a]The ABCD & RAINE STUDY cohorts only included single births in their sample.

[b]Level of education based on the highest on-going or completed education when the child was 0 year old (between >-1 year and <1 year). If more than one education level is reported within the defined time frame, we used highest recorded education level.
Classification according to International Standard Classification of Education 97/2011 (ISCED-97/2011)
High: Short cycle tertiary, Bachelor, Masters, Doctoral or equivalent (ISCED-2011: 5-8, ISCED-97: 5-6)
Medium: Upper secondary, Post-secondary non-tertiary (ISCED-2011: 3-4, ISCED-97: 3-4)
Low: No education; early childhood; pre-primary; primary; lower secondary or second stage of basic education. (ISCED-2011: 0-2, ISCED-97: 0-2)

[c]Eusilc income quintiles for all cohorts except for RAINE (AU) and ABCD (NL) that measured household income in quartiles.

M1b: Basic child et mother's controls at birth
M2b: M1b + socioeconomic variables
M3b: M2b + mother's smoking during pregnancy

For peer review only - http://bmjopen.bmj.com/site/about/guidelines.xhtml

BMJ Open

Differences in birthweight between immigrants' and natives' children in Europe and Australia: A LifeCycle comparative observational cohort study

Journal:	*BMJ Open*
Manuscript ID	bmjopen-2022-060932.R1
Article Type:	Original research
Date Submitted by the Author:	03-Feb-2023
Complete List of Authors:	Florian, Sandra; INED<br>Ichou, Mathieu; INED<br>Panico, Lidia; INED<br>Pinel-Jacquemin, Stéphanie; Université Toulouse Jean Jaurès, UMR5193, LISST-CERS<br>Vrijkotte, Tanja G. M.; Amsterdam Public Health Research Institute, Department of Public Occupational Health<br>Harskamp-van Ginkel, Margreet; Amsterdam Public Health Research Institute, Department of Public Occupational Health<br>Huang, Rae-Chi; Edith Cowan University School of Medical and Health Sciences, Nutrition and Health Innovation Research Institute<br>Carson, Jennie; University of Western Australia, Telethon Kids Institute<br>Rodriguez, Loreto; Ministry of Health of the Basque Government, Sub Directorate for Public Health and Addictions of Gipuzkoa; Biodonostia Health Research Institute, Group of Environmental Epidemiology and Child Development<br>Subiza-Pérez, Mikel; Biodonostia Health Research Institute, Group of Environmental Epidemiology and Child Development; Instituto de Salud Carlos III, Spanish Consortium for Research on Epidemiology and Public Health (CIBERESP)<br>Vrijheid, Martine; CIBERESP, Instituto de Salud Carlos III; ISGlobal<br>Fernandez, Sílvia; ISGlobal, Agència de Salut Pública de Barcelona<br>Yang, Tiffany; Bradford Institute for Health Research, Born in Bradford<br>Wright, John; Bradford Institute for Health Research, Bradford Teaching Hospitals NHS Foundation Trust<br>Corpeleijn, Eva; University of Groningen, Department of Epidemiology, GECKO Drenthe Cohort, University Medical Center Groningen<br>Cardol, Marloes; University of Groningen, Department of Epidemiology, GECKO Drenthe Cohort, University Medical Center Groningen<br>Isaevska, Elena; Universita degli Studi di Torino Dipartimento di Scienze Mediche<br>Moccia, Chiara; University of Turin, Department of Medical Sciences<br>Kooijman, Marjolein; Erasmus Medical Center, The Generation R Study Group, University Medical Center; Erasmus Medical Centre Department of General Pediatrics, University Medical Center<br>Voerman, Ellis; Erasmus Medical Center, The Generation R Study Group, University Medical Center; Erasmus Medical Centre Department of General Pediatrics, University Medical Center<br>Jaddoe, Vincent; Erasmus Medical Center, The Generation R Study

	Group, University Medical Center; Erasmus Medical Centre Department of General Pediatrics, University Medical Center<br>Welten, Marieke; Erasmus Medical Center, The Generation R Study Group; Erasmus Medical Center, Department of Pediatrics<br>Spada, Elena; Meyer Children's University Hospital, Unit of Epidemiology<br>REBAGLIATO, MARISA; Universitat Jaume I, Predepartamental Unit of Medicine; CIBERESP<br>Beneito, Andrea; FISABIO, Joint Research Unit in Epidemiology, Environment and Health<br>Ronfani, Luca; Istituto di Ricovero e Cura a Carattere Scientifico materno infantile Burlo Garofolo, Clinical Epidemiology and Public Health Research Unit<br>Charles, Marie-Aline; Inserm and INED joint research group; Inserm (U1018), CESP

**Primary Subject Heading**:	Epidemiology
Secondary Subject Heading:	Health policy
Keywords:	EPIDEMIOLOGY, Health policy < HEALTH SERVICES ADMINISTRATION & MANAGEMENT, Community child health < PAEDIATRICS, PUBLIC HEALTH, STATISTICS & RESEARCH METHODS

SCHOLARONE™ Manuscripts

I, the Submitting Author has the right to grant and does grant on behalf of all authors of the Work (as defined in the below author licence), an exclusive licence and/or a non-exclusive licence for contributions from authors who are: i) UK Crown employees; ii) where BMJ has agreed a CC-BY licence shall apply, and/or iii) in accordance with the terms applicable for US Federal Government officers or employees acting as part of their official duties; on a worldwide, perpetual, irrevocable, royalty-free basis to BMJ Publishing Group Ltd ("BMJ") its licensees and where the relevant Journal is co-owned by BMJ to the co-owners of the Journal, to publish the Work in this journal and any other BMJ products and to exploit all rights, as set out in our licence.

The Submitting Author accepts and understands that any supply made under these terms is made by BMJ to the Submitting Author unless you are acting as an employee on behalf of your employer or a postgraduate student of an affiliated institution which is paying any applicable article publishing charge ("APC") for Open Access articles. Where the Submitting Author wishes to make the Work available on an Open Access basis (and intends to pay the relevant APC), the terms of reuse of such Open Access shall be governed by a Creative Commons licence – details of these licences and which Creative Commons licence will apply to this Work are set out in our licence referred to above.

Other than as permitted in any relevant BMJ Author's Self Archiving Policies, I confirm this Work has not been accepted for publication elsewhere, is not being considered for publication elsewhere and does not duplicate material already published. I confirm all authors consent to publication of this Work and authorise the granting of this licence.

For peer review only

Differences in birthweight between immigrants' and natives' children in Europe and Australia: A LifeCycle comparative observational cohort study

Authors

* Florian, Sandra, Institut National d'Études Démographiques (INED), 9 cours des Humanités, 93322 Aubervilliers, France, sandra.florian@ined.fr, Tel. : +33 1 56 06 22 04

Ichou, Mathieu, Institut National d'Études Démographiques (INED), Aubervilliers, France

Panico, Lidia, Institut National d'Études Démographiques (INED), Aubervilliers, France

Pinel-Jacquemin, Stéphanie; Université Toulouse Jean Jaurès, UMR5193, LISST-CERS, Toulouse, France

Vrijkotte, Tanja G. M.; Amsterdam Public Health Research Institute, Department of Public Occupational Health, Amsterdam, Netherlands

Harskamp-van Ginkel, Margreet; Amsterdam Public Health Research Institute, Department of Public Occupational Health, Amsterdam, Netherlands

Huang, Rae-Chi; Edith Cowan University School of Medical and Health Sciences, Nutrition and Health Innovation Research Institute, Perth, Australia

Carson, Jennie; University of Western Australia, Telethon Kids Institute, Perth, Australia

Rodriguez, Loreto; Ministry of Health of the Basque Government, Sub Directorate for Public Health and Addictions of Gipuzkoa; Biodonostia Health Research Institute, Group of Environmental Epidemiology and Child Development, San Sebastián, Spain

Subiza-Pérez, Mikel; Biodonostia Health Research Institute, Group of Environmental Epidemiology and Child Development; Instituto de Salud Carlos III, Spanish Consortium for Research on Epidemiology and Public Health (CIBERESP), Madrid, Spain

Vrijheid, Martine; CIBERESP, Instituto de Salud Carlos III; ISGlobal, Barcelona, Spain

Fernandez, Sílvia; ISGlobal, Agència de Salut Pública de Barcelona, Barcelona, Spain

Yang, Tiffany; Bradford Institute for Health Research, Born in Bradford, Bradford, UK

Wright, John; Bradford Institute for Health Research, Bradford Teaching Hospitals NHS Foundation Trust, Bradford, UK

Corpeleijn, Eva; University of Groningen, Department of Epidemiology, GECKO Drenthe Cohort, University Medical Center Groningen, Groningen, Netherlands

Cardol, Marloes; University of Groningen, Department of Epidemiology, GECKO Drenthe Cohort, University Medical Center Groningen, Groningen, Netherlands

Isaevska, Elena; Universita degli Studi di Torino Dipartimento di Scienze Mediche, Torino, Italy

Moccia, Chiara; University of Turin, Department of Medical Sciences, Torino, Italy

Kooijman, Marjolein; Erasmus Medical Center, The Generation R Study Group, University Medical Center; Erasmus Medical Centre Department of General Pediatrics, Rotterdam, Netherlands

Voerman, Ellis; Erasmus Medical Center, The Generation R Study Group, University Medical Center; Erasmus Medical Centre Department of General Pediatrics, Rotterdam, Netherlands

Jaddoe, Vincent; Erasmus Medical Center, The Generation R Study Group, University Medical Center; Erasmus Medical Centre Department of General Pediatrics, Rotterdam, Netherlands

Welten, Marieke; Erasmus Medical Center, The Generation R Study Group; Erasmus Medical Center, Department of Pediatrics, Rotterdam, Netherlands

Spada, Elena; Meyer Children's University Hospital, Unit of Epidemiology, Florence, Italy

Rebagliato, Marisa; Universitat Jaume I, Predepartamental Unit of Medicine; CIBERESP, Madrid, Spain

Beneito, Andrea; FISABIO, Joint Research Unit in Epidemiology, Environment and Health, Valencia, Spain

Ronfani, Luca; Istituto di Ricovero e Cura a Carattere Scientifico materno infantile Burlo Garofolo, Clinical Epidemiology and Public Health Research Unit, Trieste, Italy

Charles, Marie-Aline; Inserm and INED joint research group; Inserm (U1018), CESP, Paris, France

* Corresponding author

For peer review only - http://bmjopen.bmj.com/site/about/guidelines.xhtml

Key words: birth weight, child health, health inequalities, cohort studies, life course epidemiology.

Word count: 3902

ABSTRACT

Objective: Research on adults has identified an immigrant health advantage, known as the "immigrant heath paradox", by which migrants exhibit better health outcomes than natives. Is this health advantage transferred from parents to children in the form of higher birthweight relative to children of natives?

Setting: Western Europe and Australia.

Participants: We use data from nine birth cohorts participating in the LifeCycle Project, including five studies with large samples of immigrants' children: ELFE-France (*N*=12,494), the Raine study-Australia (*N*=2,283), BiB-UK (*N*=4,132), ABCD study-Netherlands (*N*=4,030), and the Generation R study-Netherlands (*N*=4,877). We include male and female babies born to immigrant and native parents.

Primary and secondary outcome measures: The primary outcome is birthweight measured in grams. Different specifications were tested: Birthweight as a continuous variable including all births (DV1), the same variable but excluding babies born with over 4500 grams (DV2), low birthweight as a 0-1 binary variable (1 = birthweight below 2500 grams) (DV3). Results using these three measures were similar, only results using DV1 are presented. Parental migration status is measured in four categories: both parents natives, both born abroad, only mother born abroad, and only father born abroad.

Results: Two patterns in children's birthweight by parental migration status emerged: higher birthweight among children of immigrants in France (+12g, $p<.10$) and Australia (+40g, $p<.10$) and lower birthweight among children of immigrants in the UK (-82g, $p<.05$) and the Netherlands (-80g and -73g, $p<.001$) compared to natives' children. Smoking during pregnancy emerged as a mechanism explaining some of the birthweight gaps between children of immigrants and natives.

Conclusion: The immigrant health advantage is not universally transferred to children in the form of higher birthweight in all host countries. Further research should investigate whether this cross-national variation is due to differences in immigrant communities, social and health care contexts across host countries.

For peer review only

Strengths and Limitations

- This study includes data from nine cohorts in six countries, allowing for a comparison of varied national contexts and diverse immigrant groups.
- Results are based on a large combined sample of more than 38,000 children.
- The use of harmonized health outcomes and socio-demographic variables, as well as standardized statistical analyses enables a rigorous multinational comparative study.
- Yet, some of the cohort samples are representative at the national level while others at the local level, which limits the generalizability of the findings for certain cohorts.
- Some of the most vulnerable, recently arrived immigrant groups may be underrepresented in certain cohorts, potentially biasing the results for these immigrant communities.

INTRODUCTION

Birthweight is an important health indicator that has been associated with lifelong development, morbidity and mortality.[1–4] Studies have found a U-shape relationship between birthweight and the risk of developing health problems, including cardiometabolic complications, with both low and high birthweight associated with a higher risk.[4]

Research on adults has identified an immigrant health advantage, known as the "immigrant heath paradox",[5–7] by which migrants appear to have better health outcomes, including lower morbidity and mortality as well as better mental health and fewer health risk behaviors, than their native counterparts, despite being on average more socioeconomically disadvantaged.[8] This health advantage has been hypothesized to be linked to positive pre-migration selectivity.[7] Studies using American data suggest that this health advantage can be transferred from parents to children, at least during the early years, finding healthier birthweight (i.e ≥ 2,500g and ≤ 4,500g) and lower

For peer review only - http://bmjopen.bmj.com/site/about/guidelines.xhtml

For peer review only

probability of low birthweight among children of Hispanic immigrants in the USA[4,9,10] compared to native peers. This advantage does not appear to apply to second-generation parents and their children.[11]

There is less research on these relationships in Europe, although the available evidence suggests similar patterns, i.e., better birth health of children born to first generation migrant mothers.[12] Little research has however investigated patterns in children's birthweight by parental migration status through a cross-country comparative lens, particularly in Europe.[13] Investigating inequality in children's birthweight according to parents' migration status is important given its potential to inform social and health policies. Differences in national origin and host country contexts (social policies, health systems, economic opportunities, etc.) are likely to shape inequalities in children's birth outcomes as well as immigrant parents' abilities to invest in their children's health and transfer them health "capital".[2] Thus, we expect that children' birthweight by parental migrant status will vary across host countries.

Parental socioeconomic status is also an important determinant of children's health outcomes,[8,14,15] and, hence, a potential confounder of the relationship between migration status and birthweight. Children of less educated parents and with low family incomes are more likely to exhibit lower birthweight.[6,16,17] This has led researchers to highlight the importance of considering parental socioeconomic background when investigating differences in birth outcomes according to migrant status.[18] Although immigrants in developed countries tend to be socioeconomically disadvantaged on average, the level of this disadvantage differs across host countries; it is therefore even more critical to consider socio-economic status in comparative research.[18]

Parental health behaviors also constitute an important possible mechanism that may contribute to health inequality at birth.[19] The deleterious effects of mother's smoking during pregnancy for children's health at birth are well established in the literature.[20–23] Research indicates

For peer review only - http://bmjopen.bmj.com/site/about/guidelines.xhtml

that immigrant mothers appear to have fewer health risk behaviors, such as less smoking and lower alcohol consumption.[22] We explore the role of maternal health behavior (smoking during pregnancy) as a potential mechanism shaping inequalities in birthweight across migrant groups.

This study focuses on the differences in birthweight between children of natives and children of immigrants using recent data from eight European countries and Australia to investigate three questions. First, do children of immigrants exhibit higher or lower birthweight relative to children of natives? Second, how do these associations vary across host countries? Third, can maternal smoking during pregnancy explain any of these differences?

METHODS

Data

Data come from the Horizon 2020-funded LifeCycle Project-EU Child Cohort Network,[24] which comprises nineteen pregnancy and childhood cohorts in Europe and Australia investigating early life health and its determinants, starting from pregnancy. LifeCycle cohorts collected data on demographic and socio-economic characteristics, including maternal health, marital status, living arrangements, parental education, family income, and parental health behaviors, amongst other variables.[24]

A total of nine cohorts collected data on migration status and were able to harmonize variables on socioeconomic and migrant status associated with health at birth. The results presented in the main text are based on five cohorts with a sufficiently large sample size (> 1,700 children of immigrants) to conduct multivariate analyses. These cohorts include: the Etude Longitudinale Française depuis l'Enfance ELFE (France), a nationally representative cohort study following 18,329 births in France to mothers aged 18 and over in 2011;[25] the Raine study (Australia), a prospective study of 2,788 babies collecting data starting in 1989 from pregnancy

For peer review only - http://bmjopen.bmj.com/site/about/guidelines.xhtml

onwards;[26] the Born in Bradford BiB study (UK) that includes data from 13,524 children born during 2007–2011 in Bradford;[27] the Amsterdam Born Children and their Development ABCD study (Netherlands) including data from 11,474 pregnancies in Amsterdam between 2003 and 2004;[28] and the Generation R study (Netherlands) which enrolled 9,153 mothers with a delivery date from April 2002 until January 2006, with a total of 9,747 live born children.[29]

The other participating cohorts with smaller sample sizes (< 1,700 children of immigrants) included the NINFEA study (Italy),[30] the Piccolipiù study (Italy),[31] the INfancia y Medio Ambiente (Environment and Childhood) INMA study (Spain)[32] and the GECKO Drenthe study (Netherlands).[33] Because of smaller sample sizes, it is more difficult to draw conclusions from these cohorts, nevertheless, Appendix 1 presents the sample distribution and children's birthweight by parental migration status and maternal region of birth for participating cohorts with a small number of children of immigrants. Model results for these smaller-sample cohorts are presented in Appendix 2.

Patient and Public Involvement

Participants were recruited prior to and during pregnancy, as well as in childhood. All data have been deidentified. Ethical and legal responsibility for data management and security is maintained by the source studies or home institutions.[24]

Measures

The dependent variable is the child's birthweight, a continuous variable measured in grams. Different specifications of our dependent variable are tested (see Data analysis section below). Children's migration status is measured in four categories: second generation children, with both parents born abroad; 2.5 generation children with a mother born abroad, and native-born father; 2.5 generation children with a father born abroad and a native-born mother; and natives (reference), which included children with both parents born in the host country. Mothers' region of birth was

For peer review only - http://bmjopen.bmj.com/site/about/guidelines.xhtml

measured in ten categories: host country (reference for each cohort study), Western Europe, Eastern Europe, Other Europe and Central Asia, East Asia and Pacific, South Asia, Middle East and North Africa, Sub-Saharan Africa, Latin America and the Caribbean, and North America.

Child characteristics at birth that were used as control variables included: the child's sex; a binary indicator for multiple births (single birth, multiple birth); birth order measured with a continuous variables indicating the mother's parity; and the child's gestational age (in days).

Basic controls for mother's characteristics included maternal height (cm) and pre-pregnancy weight (kg), measured as continuous variables.

We consider two indicators of socioeconomic background: mother's education and household income at the child's birth. Mother's level of education was measured in three categories: high (reference), medium, and low education. Household income quintile was measured using a cohort-specific log of the equivalized total disposable household monthly income in 2011, predicted using pan-European Union Statistics on Income and Living Conditions (EUSILC) data.[34] The Australian Raine Study and the Dutch ABCD cohort used a household income quartile measure (the fourth quartile being the richest), collected at ages 1 and 5 respectively.

Finally, we included a binary indicator of mother's smoking behavior during pregnancy (0 = no smoking during pregnancy, 1 = any smoking during the pregnancy). Each of the participating cohorts conducted the variable harmonization for all the variables, following strict step-by-step protocols (see https://pubmed.ncbi.nlm.nih.gov/33884544/). Quality control checks were conducted to ensure the correct harmonization of variables across cohorts.

For peer review only - http://bmjopen.bmj.com/site/about/guidelines.xhtml

Data analysis

After descriptive statistics analyses, we selected our analytical sample following a complete-case analysis approach. We used ordinary least square (OLS) and logistic regressions to model different specifications of birthweight. First, we modelled birthweight as a continuous variable, including all births (DV1). Second, we excluded babies born with a birthweight higher than 4500 grams (DV2) given their higher risk of negative health outcomes. Finally, we modelled low birthweight as a 0-1 binary variable where 1 indicates birthweight below 2500 grams (DV3). As the results for these three models were very similar, only DV1 is presented in the results section. Models using the other specifications are available as supplementary material (Appendix 3 & 4).

Analyses were conducted separately for each cohort. Each cohort conducted two sets of models for all three birthweight specifications. The first set predicted birthweight regressed on children's migration status, while the second set regressed birthweight on maternal region of birth. Each set of models included three nested models. Model 1 (M1) controlled for basic characteristics at birth, including the child's sex, birth order, gestational age, and the mother's height and pre-pregnancy weight. Model 2 (M2) additionally controlled for family socioeconomic characteristics, including the mothers' education and family income. Model 3 (M3) further adjusted for mothers' smoking during pregnancy.

RESULTS

Birthweight differences by parental migration status

Table 1 presents the sample distribution and the average birthweight for children by parental migration status and maternal region of birth for the five largest cohorts, as well as the distribution of cases excluded from the analysis due to missing values. As Table 1 shows, immigrant group representation varies by cohort. The largest immigrant groups in Raine (AU)

For peer review only - http://bmjopen.bmj.com/site/about/guidelines.xhtml

**Table 1 Cohort sample distribution & children's birthweight (in grams) by migration status and mother's region of origin (large cohorts)**															
	**Raine (AU)**			**ELFE (FR)**			**BiB (UK)**			**ABCD (NL)**			**Gen R (NL)**		
	**Freq.**	**%**	**Weight Mean**	**Freq.**	**%**	**Weight Mean**	**Freq.**	**%**	**Weight Mean**	**Freq.**	**%**	**Weight Mean**	**Freq.**	**%**	**Weight Mean**
**Children's migration status**															
2nd Generation	697	25.0	3314	1289	7.0	3349	2488	18.5	3186	2165	18.9	3313	1887	19.4	3321.036
2.5 Gen. (immigrant mother)	407	14.6	3303	918	5.0	3344	1595	11.7	3112	743	6.5	3393	767	7.9	3397
2.5 Gen. (immigrant father)	437	15.7	3363	1159	6.3	3348	1831	13.5	3120	943	8.2	3362	1040	10.7	3318
Natives	1137	40.8	3288	13257	72.3	3308	5224	38.7	3293	3957	34.5	3455	4588	47.1	3452
Missing	110	3.9	2677	1706	9.3	3243	2386	17.6	3162	3666	32.0	3368	1465	15.0	3309
**Total**	**2788**	**100**	**3284**	**18329**	**100**	**3309**	**13524**	**100**	**3205**	**11474**	**100**	**3389**	**9747**	**100**	3387
**Mother's region of birth**															
Host country-born	1608	57.7	3305	14839	80.96	3307	7084	52.4	3247	4985	43.4	3435	5929	60.8	3419
Western EU/EEA	620	22.2	3344	278	1.5	3313	91	0.7	3256	401	3.5	3423	436	4.5	3408
Eastern EU	33	1.2	3414	101	0.6	3410	243	1.8	3388	67	0.6	3390	56	0.6	3333
Other Europe & Central Asia	0	0	0	140	0.8	3341	27	0.2	3490	390	3.4	3384	587	6.0	3422
East Asia & Pacific	300	10.8	3264	129	0.7	3354	121	0.9	3272	151	1.3	3409	200	2.1	3304
South Asia	55	2.0	3152	23	0.1	3162	3291	24.2	3118	145	1.3	3199	31	0.3	3393

For peer review only

For peer review only - http://bmjopen.bmj.com/site/about/guidelines.xhtml

Middle East & North Africa	15	0.5	3298	858	4.7	3392	78	0.6	3423	729	6.4	3444	533	5.5	3531
Sub-Saharan Africa	60	2.2	3219	604	3.3	3297	219	1.7	3232	344	3.0	3226	363	3.7	3236
Latin America & Caribbean	16	0.6	3320	125	0.7	3230	12	0.1	3011	666	5.8	3201	884	9.1	3200
North America	12	0.4	3404	18	0.1	3131	15	0.1	3354	67	0.6	3421	39	0.4	3391
Missing	69	2.5	2412	1214	6.6	3282	2343	17.3	3164	3529	30.8	3366	689	7.1	3300
**Total**	**2788**	**100**	**3284**	**18329**	**100**	**3309**	**13524**	**100**	**3205**	**11474**	**100**	**3389**	**9747**	**100**	3387
***n* Missing cases in analyses**	***n***		**Mean**	***n***		**Mean**	***n***		**Mean**	***n***		**Mean**	***n***		**Mean**
Birthweight	80		---	503		---	334		---	921		---	90		---
Child & mother's controls	112		2520	531		3301	8097		3197	3307		3370	2376		3349
Migration status	44		3178	1495		3239	2386		3162	148		3304	636		3326
Missing other variables	269		3286	3306		3261	3564		3183	3068		3311	1768		3341
**Total excluded cases**	**505**		**2985**	**5835**		**3259**	**9392**		**3198**	**7444**		**3344**	**4870**		**3343**

For peer review only

come from Western Europea and East Asian and Pacific countries. For ELFE (FR) the largest groups are immigrants from Middle East and North Africa followed by Sub-Saharan African. For BiB (UK) the largest group come from South Asia; whereas in ABCD (NL) and Generation R (NL) the groups with the largest representations come from Latin America, the Carribean as well as Middle East and North Africa.

Table 2 presents the first set of models predicting birthweight regressed on children's migration status and Figures 1 and 2 illustrate the results of Table 2. We observed two distinct patterns regarding the disparities of children's weight at birth by parental migration status in the five largest cohorts (Table 2 & Figures 1 and 2). The "small sample" cohorts exhibited similar patterns, but the differences were not statistically significant, possibly due to small samples of children of immigrants (Appendix 2).

In the first pattern, depicted by two cohorts, ELFE (France) and Raine (Australia), children of immigrants exhibited on average higher weight at birth relative to children of natives, as shown in Figure 1. For ELFE, Model M1 (gray bars) shows a slight tendency for children of immigrants to be born with higher weight than children with both native parents only among 2.5 generation, immigrant mother (+48g, $p<.001$). For the ELFE cohort, adjusting for socioeconomic characteristics in Model M2 (white bars), children of immigrants' weight *increased* compared to natives and was significant for the 2nd generation (+43g, $p<.05$) and the 2.5 generation mother (+54g, $p<.001$). By contrast, for the Raine Study, controlling for socioeconomic factors somewhat *decreased* children of immigrants' birthweight relative to children of natives, the few marginally significant differences became non-statistically significant. Model M3, which additionally controlled for mothers' smoking during pregnancy (black bars), showed a reduction in the

For peer review only - http://bmjopen.bmj.com/site/about/guidelines.xhtml

Table 2 OLS regression coefficients of children's migration status on child's birthweight (large cohorts)

	ELFE (FR)						RAINE STUDY (AU)						BIB (UK)					
	M1		M2		M3		M1		M2		M3		M1		M2		M3	
**DV1: Birthweight**	Child controls		SES		Smoked		Child controls		SES		Smoked		Child controls		SES		Smoked	
**Children's migration status**																		
Natives (ref.)																		
2nd generation	11.9		42.5	*	13.0		39.8		34.4		16.7		-81.5	***	-83.4	***	-118.8	***
2.5 generation (mother)	47.6	**	53.9	***	43.2	**	41.4		38.0		32.6		-97.8	***	-94.7	***	-132.8	***
2.5 generation (father)	3.2		16.7		11.6		48.1		46.7		38.2		-117.0	***	-120.8	***	-147.8	***
**Child controls**																		
Female	-149.3	***	-149.2	***	-150.2	***	-130.2	***	-129.8	***	-123.3	***	-145.4	***	-143.5	***	-144.6	***
Plural birth[a]	-372.1	***	-374.2	***	-378.0	***	0.0		0.0		0.0		-311.2	***	-305.0	***	-309.9	***
Mother's parity (birth order)	49.9	***	54.9	***	54.0	***	64.8	***	65.3	***	64.7	***	54.8	***	62.7	***	59.8	***
Gestational age	23.7	***	23.6	***	23.5	***	20.0	***	20.0	***	20.1	***	25.3	***	25.3	***	25.2	***
**Mother controls**																		
Height	9.0	***	8.5	***	8.7	***	11.0	***	10.7	***	11.0	***	11.3	***	10.9	***	11.1	***
Pre-pregnancy weight	4.7	***	5.0	***	4.9	***	5.6	***	5.7	***	5.5	***	5.6	***	5.6	***	5.6	***
**SES**																		
**Education[b]**																		
High (ref.)																		
Medium			-11.0		-2.7				16.4		18.5				-0.7		1.7	
Low			-34.8	*	-15.9				-1.0		19.5				-24.9		-21.9	
**Household income quintiles[c]**																		
1st quintiles (ref.)																		
2nd quintile			52.3	***	44.9	***			-6.2		4.9				1.7		-9.7	
3th quintile			50.2	***	32.9	*			-31.4		-13.7				-38.0	.	-22.9	
4th quintile			57.3	***	36.9	*			-30.6		2.3				-38.8	.	-27.7	
5th quintile			60.8	***	36.4	*			-		-				-68.6	**	-31.0	

For peer review only

**Mother smoked (pregnancy)**					-118.6	***					-151.6	***					-135.7	***
**Constant**	**-5049.5**	***	**-4996.3**	***	**-4972.1**	***	**-4281.4**	***	**-4236.2**	***	**-4261.9**	***	**-5889.6**	***	**-5810.7**	***	**-5766.4**	***
N	12494		12494		12494		2283		2283		2283		4132		4132		4132	
**R^2**	0.358		0.361		0.369		0.379		0.38		0.393		0.489		0.492		0.498	
$*p < .05$, $**p < .01$, $***p < .001$																		

[a]The ABCD & RAINE STUDY cohorts only included single births in their sample.
[b]Level of education based on the highest on-going or completed education when the child was 0-year-old (between >-1 year and <1 year). If more than one education level is reported within the defined time frame, we used highest recorded education level. Classification according to International Standard Classification of Education 97/2011 (ISCED-97/2011)
High: Short cycle tertiary, Bachelor, Masters, Doctoral or equivalent (ISCED-2011: 5-8, ISCED-97: 5-6)
Medium: Upper secondary, Post-secondary non-tertiary (ISCED-2011: 3-4, ISCED-97: 3-4)
Low: No education; early childhood; pre-primary; primary; lower secondary or second stage of basic education (ISCED-2011: 0-2, ISCED-97: 0-2)
[c]Eusilc income quintiles for all cohorts except for RAINE (AU) and ABCD (NL) that measured household income in quartiles.
M1: Basic child et mother's controls at birth
M2: M1 + socioeconomic variables
M3: M2 + mother's smoking during pregnancy

For peer review only

For peer review only - http://bmjopen.bmj.com/site/about/guidelines.xhtml

**Table 2 Continued**												
	**ABCD (NL)**						**GEN R (NL)**					
	**M1**		**M2**		**M3**		**M1**		**M2**		**M3**	
**DV1: Birthweight**	**Child controls**		**SES**		**Smoked**		**Child controls**		**SES**		**Smoked**	
**Children's migration status**												
Natives (ref.)												
2nd generation	-80.1	***	-52.6	*	-66.5	**	-73.4	***	-33.6		-50.2	*
2.5 generation (mother)	14.6		23.3		14.9		26.7		42.5		36.4	
2.5 generation (father)	-78.3	***	-65.6	**	-61.5	**	-75.3	***	-53.9	**	-51.1	**
**Child controls**												
Female	-121.9	***	-123.4	***	-123.6	***	-106.8	***	-107.2	***	-108.2	***
Plural birth[a]	0.0		0.0		0.0		-404.8	***	-408.5	***	-409.1	***
Mother's parity (birth order)	82.0	***	84.3	***	83.1	***	109.3	***	111.8	***	109.3	***
Gestational age	25.7	***	25.6	***	25.6	***	26.1	***	26.0	***	25.9	***
**Mother controls**												
Height	10.4	***	9.5	***	9.5	***	10.1	***	9.2	***	9.3	***
Pre-pregnancy weight	4.6	***	5.0	***	4.9	***	4.5	***	4.8	***	4.7	***
**SES**												
**Education[b]**												
High (ref.)												
Medium			-54.9	***	-44.1	**			-10.0		-7.3	
Low			-59.7	**	-38.2				-30.7		-23.0	
**Household income quintiles[c]**												
1st quintiles (ref.)												
2nd quintile			2.7		5.9				21.4		15.2	
3th quintile			-3.9		5.9				56.1	*	42.9	
4th quintile			-30.6		-18.1				77.1	*	56.4	
5th quintile			-		-				84.2	*	63.1	
**Mother smoked (pregnancy)**					-135.5	***					-79.5	***
**Constant**	**-5733.5**	***	**-5573.2**	***	**-5550.5**	***	**-5951.2**	***	**-5823.6**	***	**-5780.1**	***
N	4,030		4,030		4,030		4877		4877		4877	
**R^2**	0.401		0.404		0.409		0.46		0.46		0.46	
**p < .05, **p < .01, ***p < .001*												

For peer review only

magnitude of the coefficients for children of immigrants for both cohorts, which is still significant and positive for ELFE's 2.5 generation mother (+43g, $p<.01$).

[Insert Fig. 1 about here]

The opposite pattern is observed in Figure 2 Model 1, where children of immigrants exhibit lower birthweights compared to children of natives. This pattern characterizes the BiB (Bradford, UK), the ABCD (Amsterdam, Netherlands), and the Generation R (Rotterdam, Netherlands) cohorts. Exceptions to this pattern are 2.5 generation children with an immigrant mother in the two Dutch cohorts, where no significant differences from natives' birthweight could be detected.

For the Dutch cohorts (ABCD and Generation R), the differences in birthweight for children with two immigrant parents and for those with one immigrant father are somewhat reduced after controlling for socioeconomic characteristics (Model M2), suggesting that the lower birthweight might be due to immigrants' disadvantaged socioeconomic position within the host country. By contrast socioeconomic factors do not seem to play an important role in explaining birthweight differences for the BiB (UK) cohort[35].

[Insert Fig. 2 about here]

As in the first group, mothers' smoking during pregnancy had a negative effect on children's birthweight. Despite mothers' lower prevalence of smoking during pregnancy, children of immigrants' still exhibit lower birthweight than children of natives after controlling for this variable. As shown in Figure 2, for BiB birthweight gaps tend to increase in magnitude after controlling for smoking during pregnancy (Model M3, 2nd generation -119g, $p<.001$, 2.5 gen-mother -133gr, $p<.001$ and 2.5 gen-father -148gr, $p<.001$), indicating that if immigrant mothers

For peer review only - http://bmjopen.bmj.com/site/about/guidelines.xhtml

smoked as much as native mothers, children of immigrants' birthweight would be even lower. For ABCD and GEN R, smoking has a smaller effect, but it slightly increased the gap for the second generation (-67g, $p<.01$ for ABCD, and -50g, $p<.05$ for GEN R). The mothers' height and pre-pregnancy weight is positively and similarly associated with the child's birthweight for all cohorts.

In sum, we find higher birthweight for children of immigrants in our southern European studies, such as in France and in the smaller cohorts representing Italy and Spain, as well as in Australia; and lower birthweight for children of immigrants in central and northern European countries, such as the UK and the Netherlands. These patterns could result from differences in the immigrants' regions of origin across host countries. We therefore analyze birthweight differences by maternal region of origin in the next section.

Birthweight differences by maternal region of birth

Results from the models by mothers' region of origin are presented in Table 3. First, we observe heterogeneity in birthweight by immigrant mothers' regions of birth. For ELFE (France), the pattern of birthweight "advantage" and the changes across models are mostly driven by children of Middle East and North African immigrants, the largest immigrant group in France, as well as by children of East Asia & Pacific immigrants. However, children of Sub-Saharan Africans, the second largest immigrant group in France do not appear to have statistically significantly different birthweights relative to children of natives. Nonetheless, immigrant mothers exhibit lower smoking rates in most cohorts; thus, controlling for mother's smoking during pregnancy (Table 3) explained part of the weight "advantage" for two groups (East Asia & Pacific: +132g, $p<.01$; Middle East & North Africa: +61g, $p<.01$).

In the Raine study, children of Western Europeans, the largest immigrant group, showed slightly higher birthweight than natives, but differences were not statistically significant. Children

For peer review only - http://bmjopen.bmj.com/site/about/guidelines.xhtml
For peer review only

Table 3. OLS regression coefficients of mother's region of origin on child's birthweight (large cohorts)																		
	ELFE (FR)						RAINE STUDY (AU)						BIB (UK)					
**DV: Birthweight**	**M1**		**M2**		**M3**		**M1**		**M2**		**M3**		**M1**		**M2**		**M3**	
	**Child controls**		**SES**		**Smoked**		**Child controls**		**SES**		**Smoked**		**Child controls**		**SES**		**Smoked**	
**Mother's region of origin**																		
Host country-born (ref.)																		
Western EU/EEA	24.7		21.0		20.8		37.2		30.3		25.3		45.1		42.9		30.3	
Eastern EU	97.9		94.6		85.2		38.1		37.5		29.9		44.1		34.6		29.5	
Other Europe & Central Asia	5.7		13.8		1.0								-10.2		6.5		-13.3	
East Asia & Pacific	145.3	**	142.3	**	131.8	**	13.8		10.1		-9.0		115.3		96.1		80.2	
South Asia	211.9		211.1		244.7		-51.2		-54.2		-87.4		-74.1	***	-69.4	***	-94.4	***
Middle East & North Africa	60.4	**	86.6	***	61.2	**	40.2		40.8		36.5		-35.3		-45.0		-60.5	
Sub-Saharan Africa	-38.5		-9.8		-40.1		-35.8		-46.9		-67.9		-53.8		-58.1		-82.1	
Latin America & Caribbean	-7.1		8.7		-14.5		165.7		165.7		126.6							
North America	-67.6		-76.6		-56.7		374.4	**	373.1	**	354.9	**	108.7		86.4		70.2	
**Child controls**																		
Female	-149.5	***	-149.2	***	-150.3	***	-128.2	***	-128.3	***	-122.4	***	-143.1	***	-141.4	***	-141.9	***
Plural birth[a]	-372.7	***	-374.2	***	-378.6	***	0.0		0.0		0.0		-319.7	***	-313.2	***	-318.7	***
Mother's parity (birth order)	50.3	***	55.3	***	54.2	***	64.7	***	65.1	***	64.0	***	52.8	***	59.9	***	57.0	***
Gestational age	23.7	***	23.6	***	23.5	***	20.1	***	20.2	***	20.2	***	25.4	***	25.5	***	25.3	***
**Mother controls**																		
Height	9.1	***	8.5	***	8.8	***	10.6	***	10.3	***	10.6	***	11.9	***	11.7	***	11.9	***
Pre-pregnancy weight	4.7	***	5.0	***	4.9	***	5.7	***	5.8	***	5.6	***	5.6	***	5.6	***	5.6	***
**SES**																		
**Education[b]**																		
High (ref)																		
Medium			-11.8		-3.2				12.6		13.7				0.1		2.2	

For peer review only

For peer review only - http://bmjopen.bmj.com/site/about/guidelines.xhtml

Low			-34.4		-15.1				-6.5		13.7				-17.4		-13.6	
**Household income quintiles[c]**																		
1st quintile (ref.)																		
2nd quintile			51.2	***	43.8	***			-4.8		7.6				2.4		-6.1	
3th quintile			49.6	***	32.2	*			-35.3		-15.1				-44.4		-33.5	
4th quintile			55.1	***	34.4	*			-37.1		2.3				-43.7	*	-35.7	
5th quintile			58.4	***	33.9	*									-63.5	**	-32.2	
**Mother smoked (pregnancy)**					-119.8	***					-157.5	***					-108.9	***
**Constant**	**-5043**	***	**-4992**	***	**-4965**	***	**-4264**	***	**-4212**	***	**-4220**	***	**-6050**	***	**-5990**	***	**-5984**	***
N	**12419**		**12419**		**12419**		**2310**		**2310**		**2310**		**4141**		**4141**		**4141**	
**R^2**	0.358		0.36		0.369		0.38		0.38		0.4		0.485		0.488		0.492	
$*p < .05$, $**p < .01$, $***p < .001$.																		

[a]The ABCD & RAINE STUDY cohorts only included single births in their sample.
[b]Level of education based on the highest on-going or completed education when the child was 0-year-old (between >-1 year and <1 year). If more than one education level is reported within the defined time frame, we used highest recorded education level. Classification according to International Standard Classification of Education 97/2011 (ISCED-97/2011)
High: Short cycle tertiary, Bachelor, Masters, Doctoral or equivalent (ISCED-2011: 5-8, ISCED-97: 5-6)
Medium: Upper secondary, Post-secondary non-tertiary (ISCED-2011: 3-4, ISCED-97: 3-4)
Low: No education; early childhood; pre-primary; primary; lower secondary or second stage of basic education (ISCED-2011: 0-2, ISCED-97: 0-2)
[c]Eusilc income quintiles for all cohorts except for RAINE (AU) and ABCD (NL) that measured household income in quartiles.
M1: Basic child et mother's controls at birth
M2: M1 + socioeconomic variables
M3: M2 + mother’s smoking during pregnancy

For peer review only

Table 3 Continued												
	ABCD (NL)						GEN R (NL)					
DV: Birthweight	M1		M2		M3		M1		M2		M3	
	Child controls		SES		Smoked		Child controls		SES		Smoked	
**Mother's region of origin**												
Host country-born (ref.)												
Western EU/EEA	7.8		10.5		0.8		32.3		40.5		34.3	
Eastern EU	-99.4		-83.2		-90.1		68.3		99.1		78.7	
Other Europe & Central Asia	28.8		67.7		68.5		11.5		58.5	*	52.2	
East Asia & Pacific	121.3	*	134.5	*	122.7	*	124.0	**	143.3	**	127.5	**
South Asia	-174.8	**	-144.3	*	-170.9	**	11.4		35.8		9.4	
Middle East & North Africa	-6.2		25.7		1.7		20.5		63.3		38.8	
Sub-Saharan Africa	-54.6		-19.9		-41.1		-115.8	**	-68.5		-86.9	*
Latin America & Caribbean	-89.7	**	-63.0	*	-73.3	*	-71.5	**	-37.1		-49.8	*
North America	-148.5		-143.1		-151.0		-62.5		-64.6		-74.8	
**Child controls**												
Female	-121.7	***	-123.4	***	-124.0	***	-107.8	***	-108.2	***	-109.1	***
Plural birth[a]	0.0		0.0		0.0		-404.5	***	-409.0	***	-409.4	***
Mother's parity (birth order)	80.3	***	83.5	***	82.3	***	107.1	***	110.9	***	108.7	***
Gestational age	25.7	***	25.5	***	25.5	***	26.1	***	25.9	***	25.9	***
**Mother controls**												
Height	10.9	***	9.7	***	9.7	***	11.4	***	10.2	***	10.2	***
Pre-pregnancy weight	4.7	***	5.3	***	5.2	***	4.6	***	5.0	***	4.9	***
**SES**												
**Education[b]**												
High (ref)												
Medium			-66.3	***	-54.7	***			-1.9		-0.1	
Low			-78.3	***	-56.0	*			-49.7		-43.6	
**Household income quintiles[c]**												
1st quintile (ref.)												
2nd quintile			0.5		3.9				23.9		18.0	
3th quintile			-10.9		-0.5				57.2	**	44.8	*
4th quintile			-37.9		-23.5				93.6	**	73.4	*
5th quintile									100.9	**	79.6	*
**Mother smoked (pregnancy)**					-140.1	***					-77.3	***
**Constant**	**-5827**	***	**-5598**	***	**-5571**	***	**-6189**	***	**-6004**	***	**-5962**	***
N	**4,072**		**4,072**		**4,072**		**5123**		**5123**		**5123**	
**R^2**	0.398		0.402		0.408		0.456		0.461		0.465	

For peer review only

**p* < .05, ***p* < .01, ****p* < .001.													

of North American mothers also reported heavier weights at birth (M1: +374g, $p<.01$; M2: +373g, $p<.01$, M3: + 355g, $p<.01$), although these estimates are based on small sample sizes. Children with a South Asian and Sub-Saharan's background tended to have lower birthweight than children of natives, but the difference was again not statistically significant.

For BiB, the observed pattern is driven by children of South Asian immigrants, the largest immigrant group in Bradford, who weighed less at birth than children of natives (M1: -74g, $p<.001$, M2: -69g, $p<.001$, M3: -94g, $p<.001$). By contrast, children of European and East Asia and Pacific as well as North American immigrants showed a higher, but non-significant, birthweight than children of natives. The weight disadvantage among children of South Asian immigrants was partly explained by socioeconomic status. Adjusting for mother's smoking during pregnancy exacerbated this disadvantage.

For ABCD, children of women from South Asia and Latin America & Caribbean exhibited significantly lower birthweight, even after controls. Children of East Asian mothers had a significantly higher birthweight after controlling for socioeconomic variables and mother's smoking.

For Generation R (Rotterdam, the Netherlands), both patterns also coexist: children of women born in Sub-Saharan Africa and Latin America & Caribbean exhibited significantly lower birthweight, even after controls. Children of East Asian and of other European & Central Asian mothers had, on the contrary, significantly higher birthweights.

DISCUSSION

For peer review only - http://bmjopen.bmj.com/site/about/guidelines.xhtml

Research in developed countries has identified an immigrant health advantage among adults,[5,7] yet evidence on whether immigrants can transfer this health advantage to their children is mixed, and remains particularly scarce in the European context.[4,8,11,12] This study investigates whether children of first-generation immigrants exhibit a health advantage on weight at birth, comparing eight European countries and Australia.

Prior studies on the role of immigration on birthweight have focused on Latin-American immigrants in the U.S., providing mixed evidence, for example finding lighter birthweight among Mexican and Cuban babies and heavier birthweight among Puerto Rican babies relative to U.S.-born white women.[8,9] Our research extends this area of research to European countries and Australia. The first aim of this study was to evaluate whether children of immigrants exhibit a higher or lower birthweight relative to children of natives, using recent harmonized panel data of rich host countries. Two broad patterns emerged: we found higher birthweight for immigrants' children in southern European and Australian cohort studies, and lower birthweights in northern European studies, relative to natives'. This finding questions, at least when considering birth health, whether the "healthy migrant" narrative applies to all contexts, and calls for a more nuanced approach to study this phenomenon.

Our second aim was to explore in more detail birthweight variation by mother's country of origin, controlling for the mother's height and pre-pregnancy weight. Previous studies indicate that geographic origins could be associated with children of immigrants' birthweight. For example, previous work has highlighted higher birthweights for infants born in Spain to African and Latin-American mothers,[12,36] while lower mean birthweights were observed for infants born in China to Asian Indian mothers.[37] One of the most interesting findings in our study is that children of South Asian mothers showed consistently lower birthweights, in both BiB-UK (where south Asian

For peer review only - http://bmjopen.bmj.com/site/about/guidelines.xhtml

immigrants represent the largest immigrant group), ABCD-NL and to a lesser extent in the RAINE-AU study. South Asia bears half of global low birthweight burden; children of South Asian parents were consistently shown to have lower birthweight than natives in UK[38], Australia and Netherlands. Another group that stands out includes East Asian and Pacific mothers' babies, who were significantly heavier at birth in three cohorts, France and the two Dutch cohorts. In France, the largest immigrant group, immigrants from the Middle East and North Africa, showed the strongest advantage in birthweight, mirroring similar results previously found in Belgium.[18,39] Birthweights of children with Latin-American and Caribbean mothers were significantly lower in the Dutch cohorts, in contrast to findings in previous work in Spain.[12,36]

Our third aim was to evaluate the impact of mother's smoking during her pregnancy. Our results seem to go in the same direction as previous studies,[20,22] finding that mother's smoking during pregnancy has a negative effect on birthweight, as tobacco being a vasoconstrictor reduces placental circulation. Immigrant mothers' lower prevalence of smoking during pregnancy in all study cohorts had therefore a protective effect, favoring their children's health at birth across countries.

The strength of our work lies in the use of the recent and harmonized cross-country EU Child Cohort Network data.[24] We are however not able to empirically measure other important contextual factors (potential confounders) that might drive some of these patterns and that differ across countries, such as access to health services, in both origin and host countries, access to employment, exposure to discrimination, and to other health risk factors. Another methodological limitation is that the cohort samples are not all representative of the general population at the national level; some are local samples and some exclude specific groups (such as preterm babies in the Piccolipiù or ELFE cohorts). In addition, the most vulnerable and recently-arrived immigrant

For peer review only - http://bmjopen.bmj.com/site/about/guidelines.xhtml

groups, who are likely to have language difficulties and limited access to health institutions, may be underrepresented in some cohort samples. If children in these groups are lighter at birth[18,39], the current study may underreport low birthweight for children of immigrant, and thus overestimate their average birthweight. As a result, our findings can be better generalized to the largest nationally representative samples and longer established immigrant groups, and may be more limited for smaller and locally based samples, and newly arrived immigrant groups. Finally, the relationship between parental migration status and children's birthweight may change over time. This change is unlikely to meaningfully affect the comparability of our findings between cohorts, as all our cohorts include births in the 2000s and early 2010s, expect for RAINE in which babies were born in 1989. Therefore, given the large difference in data collection period, the comparison between the European and Australian (RAINE) results should be interpreted with caution.

CONCLUSION

The patterns of birthweight of children of first-generation immigrants relative to natives differ across host countries. Some of this cross-country variation seems to be due to the diverse composition of immigrant communities across Europe and Australia. Further research should investigate whether these variations are also partly driven by the different social and health policies in host countries. Improving access to healthcare, especially during pregnancy, and more inclusive social policies are needed to reduce the inequalities in birthweight, especially for disadvantaged immigrant groups, while supporting positive parental health behaviors. Our results confirm the protective effects of not smoking during pregnancy for child's birthweight, highlighting the importance of maintaining immigrants' healthier practices.

For peer review only - http://bmjopen.bmj.com/site/about/guidelines.xhtml

Research Ethics Approval

This study analyses secondary data. Each cohort study collected its own data previously and was either approved by an Ethics Committee(s) or Institutional Board(s) or exempted by them. For more information on data from each of the participating cohorts, please refer to:

Jaddoe VW V., Felix JF, Andersen AMN, et al. The LifeCycle Project-EU Child Cohort Network: a federated analysis infrastructure and harmonized data of more than 250,000 children and parents. Eur J Epidemiol. 2020;35(7):709-724. doi:10.1007/s10654-020-00662-z

Contributorship statement

LP and MI conceived the idea for the study. VJ supervised the collaboration project for all LifeCycle cohorts. SF conducted the analyses for the EFLE cohort. SF, MI, and LP wrote the statistical analysis plan. LP, MI, SF, and MAC contributed to the interpretation of results for ELFE. JW monitored data collection for the BiB study. TY and JW analyzed the data and contributed to the interpretation of the findings for the BiB study. MC and EC conducted the analysis and interpreted the results for GECKO. MW, EV, VJ and MNK and conducted the analysis for the Generation R study. TV and WMH conducted the analysis and interpreted the results for the ABCD cohort RCH and JC conducted the analyses and interpreted the results for the Raine Study. SFB, LSM, MSP, and MV analyzed data for the INMA cohort. EI, CM and LR participated in the analysis and interpretation of data for NINFEA, whereas ES, MR and AB did so for the Piccolipiù cohort. SF and SPJ coordinated the analyses among all cohorts. SF drafted the manuscript with the feedback of LP and MI. All authors revised the manuscript. All authors approved the final version of the paper.

Competing interests

The authors declare no competing interests with any commercial entity or financial or non-financial associations relating to the work in the submitted manuscript.

Acknowledgments

The Elfe survey is a joint project between the French Institute for Demographic Studies (INED) and the National Institute of Health and Medical Research (INSERM), in partnership with the French blood transfusion service (Etablissement français du sang, EFS), Santé publique France, the National Institute for Statistics and Economic Studies (INSEE), the Direction générale de la santé (DGS, part of the Ministry of Health and Social Affairs), the Direction générale de la prévention des risques (DGPR, Ministry for the Environment), the Direction de la recherche, des études, de l'évaluation et des statistiques (DREES, Ministry of Health and Social Affairs), the Département des études, de la prospective et des statistiques (DEPS, Ministry of Culture), and the Caisse nationale des allocations familiales (CNAF), with the support of the Ministry of Higher Education and Research and the Institut national de la jeunesse et de l'éducation populaire (INJEP).

The ELFE cohort also acknowledges the support from F. Zariouh, M.A. Bellance, M. Cornet, and B. de Lauzon-Guillain in harmonizing the Elfe cohort data for the Life cycle project.

For peer review only - http://bmjopen.bmj.com/site/about/guidelines.xhtml

The authors would like to acknowledge the Raine Study participants and their families for their ongoing participation in the study and the Raine Study team for study co-ordination and data collection. We also thank the NHMRC for their long term contribution to funding the study over the last 30 years. The core management of the Raine Study is funded by The University of Western Australia, Curtin University, Telethon Kids Institute, Women and Infants Research Foundation, Edith Cowan University, Murdoch University, The University of Notre Dame Australia and the Raine Medical Research Foundation.

Born in Bradford is only possible because of the enthusiasm and commitment of the Children and Parents in BiB. We are grateful to all the participants, practitioners and researchers who have made Born in Bradford happen.

The Generation R Study is conducted by the Erasmus Medical Center in close collaboration with the School of Law and Faculty of Social Sciences of the Erasmus University Rotterdam, the Municipal Health Service Rotterdam area, Rotterdam, the Rotterdam Homecare Foundation, Rotterdam and the Stichting Trombosedienst & Artsenlaboratorium Rijnmond (STAR-MDC), Rotterdam. We gratefully acknowledge the contribution of children and parents, general practitioners, hospitals, midwives and pharmacies in Rotterdam.

We also acknowledge The University of Western Australia, Curtin University, Women and Infants Research Foundation, Telethon Kids Institute, Edith Cowan University, Murdoch University, The University of Notre Dame Australia, and The Raine Medical Research Foundation for providing funding for the core management of the Raine Study.

INMA-Gipuzkoa is grateful to the participants of the study for their kind collaboration. The recruitment of participants and the development of the study was funded by grants from Instituto de Salud Carlos III (PI06/0867 and FIS-PI09/00090 incl. FEDER funds), Department of Health of the Basque Government (2005111093), Provincial Government of Gipuzkoa (DFG06/002), and annual agreements with the municipalities of the study area (Zumarraga, Urretxu , Legazpi, Azkoitia y Azpeitia y Beasain).

We are grateful to all the participating children, parents, practitioners and researchers who took part in the INMA Sabadell study. We acknowledge support from the Spanish Ministry of Science and Innovation through the “Centro de Excelencia Severo Ochoa 2019-2023” Program (CEX2018-000806-S), and support from the Generalitat de Catalunya through the CERCA Program.

The authors acknowledge the Piccolipiù Working Group and the families involved in the study.
The Childhood Obesity Project (CHOP) thanks the participating families and all project partners for their enthusiastic support of the project. Furthermore, thanks to the European Childhood Obesity Trial Study Group, who designed and conducted the study, entered the data, and participated in the data analysis.

The authors are grateful to all the participants of the NINFEA birth cohort.

We are grateful to the families who took part in the GECKO Drenthe study, the midwives, gynecologists, nurses and GPs for their help for recruitment and measurement of participants, and the whole team from the GECKO Drenthe study.

For peer review only - http://bmjopen.bmj.com/site/about/guidelines.xhtml

Funding

The LifeCycle project received funding from the European Union's Horizon 2020 research and innovation programme (Grant Agreement No. 733206 LifeCycle).

The ELFE study received a government grant managed by the National Research Agency under the "Investissements d'avenir" programme (ANR-11-EQPX-0038).

Core funding for Born in Bradford (BiB) has been funded by the Wellcome Trust (WT101597MA), a joint grant from the UK Medical Research Council (MRC) and UK Economic and Social Science Research Council (ESRC) (MR/N024397/1); the British Heart Foundation (CS/16/4/32482); and the National Institute for Health Research ARC Yorkshire and Humber (NIHR200166). The views expressed in this publication are those of the author(s) and not necessarily those of the National Institute for Health Research or the Department of Health and Social Care or Wellcome Trust.

The GECKO Drenthe birth cohort was funded by an unrestricted grant of Hutchison Whampoa Ld, Hong Kong and supported by the University of Groningen, Well Baby Clinic Foundation Icare, Noordlease, Paediatric Association Of The Netherlands, Youth Health Care Drenthe and European Union's Horizon 2020 research and innovation programme (Grant Agreement No. 733206 LifeCycle).

The Raine Study has been funded by program and project grants from the Australian National Health and Medical Research Council, the Commonwealth Scientific and Industrial Research Organisation, Healthway and the Lions Eye Institute in Western Australia. The Raine Study Gen2-17 year follow-up was funded by the NHMRC Program Grant (Stanley et al, ID 353514). The Raine Study participation in LIFECYCLE was funded by a grant from the National Health and Medical Research Council, Australia (GNT1142858). The University of Western Australia (UWA), Curtin University, the Raine Medical Research Foundation, the Telethon Kids Institute, the Women's and Infant's Research Foundation (KEMH), Murdoch University, The University of Notre Dame Australia and Edith Cowan University provide funding for the Core Management of the Raine Study.

The general design of the Generation R Study is made possible by financial support from the Erasmus MC, University Medical Center, Rotterdam, Erasmus University Rotterdam, Netherlands Organization for Health Research and Development (ZonMw), Netherlands Organisation for Scientific Research (NWO), Ministry of Health, Welfare and Sport and Ministry of Youth and Families. This project received funding from the European Union's Horizon 2020 research and innovation programme (LIFECYCLE, grant agreement No 733206, 2016; EUCAN-Connect grant agreement No 824989; ATHLETE, grant agreement No 874583). VJ received funding from a Consolidator Grant from the European Research Council (ERC-2014-CoG-648916).

INMA-Sabadell data collections were supported by grants from the Instituto de Salud Carlos III (Red INMA G03/176), CIBERESP, and the Generalitat de Catalunya-CIRIT (1999SGR 00241).
The NINFEA cohort was partially funded by the Compagnia San Paolo and by the Piedmont Region.

For peer review only - http://bmjopen.bmj.com/site/about/guidelines.xhtml

The Piccolipiù project was financially supported by the Italian National Center for Disease Prevention and Control (CCM grants years 2010 and 2014) and by the Italian Ministry of Health (art 12 and 12 bis D.lgs 502/92).

Childhood Obesity Project (CHOP): The study reported has been carried out with partial financial support from the Commission of the European Community; specific research, technological development, and demonstration program "Quality of Life and Management of Living Resources", within the European Union's Seventh Framework Programme (FP7/2007-2013); project EarlyNutrition under grant agreement no. 289346; the European Union H2020 project LIFECYCLE under grant no. 733206; the European Research Council Advanced Grant META-GROWTH (ERC-2012-AdG: no.322605) and partial financial support from the Polish Ministry of Science and Higher Education (2571/7.PR/2012/2).

Data sharing statement

This study analyses secondary data. Each cohort study collected its own data previously and was either approved by an Ethics Committee(s) or Institutional Board(s) or exempted by them. For more information on data from each of the participating cohorts, please refer to:

Jaddoe VW V., Felix JF, Andersen AMN, et al. The LifeCycle Project-EU Child Cohort Network: a federated analysis infrastructure and harmonized data of more than 250,000 children and parents. Eur J Epidemiol. 2020;35(7):709-724. doi:10.1007/s10654-020-00662-z

Patient and Public Involvement

No patient involved.

For peer review only - http://bmjopen.bmj.com/site/about/guidelines.xhtml

REFERENCES

1. Irwin LG, Siddiqi A, Hertzman C. *Early Child Development : A Powerful Equalizer. Final Report.*; 2007.
2. Kim D, Saada A. The social determinants of infant mortality and birth outcomes in western developed nations: A cross-country systematic review. *Int J Environ Res Public Health*. 2013;10(6):2296-2335. doi:10.3390/ijerph10062296
3. Gluckman PD, Hanson MA, Cooper C, Thornburg KL. Effect of In Utero and Early-Life Conditions on Adult Health and Disease. *New England Journal of Medicine*. 2008;359(1):61-73. doi:10.1056/nejmra0708473
4. Sun D, Wang T, Heianza Y, et al. Birthweight and cardiometabolic risk patterns in multiracial children. *Int J Obes*. 2018;42(1):20-27. doi:10.1038/ijo.2017.196
5. Wallace M. Adult mortality among the descendants of immigrants in England and Wales: does a migrant mortality advantage persist beyond the first generation? *J Ethn Migr Stud*. 2016;42(9):1558-1577. doi:10.1080/1369183X.2015.1131973
6. Ichou M, Wallace M. The Healthy Immigrant Effect: The role of educational selectivity in the good health of migrants. *Demogr Res*. 2019;40:61-94. doi:10.4054/DEMRES.2019.40.4
7. Guillot M, Khlat M, Elo I, Solignac M, Wallace M. Understanding age variations in the migrant mortality advantage: An international comparative perspective. *PLoS One*. 2018;13(6). doi:10.1371/journal.pone.0199669
8. Mendoza FS. Health disparities and children in immigrant families: A research agenda. *Pediatrics*. 2009;124(Suppl. 3):S187-S195. doi:10.1542/peds.2009-1100F
9. Giuntella O. The Hispanic health paradox: New evidence from longitudinal data on second and third-generation birth outcomes. *SSM Popul Health*. 2016;2:84-89. doi:10.1016/j.ssmph.2016.02.013
10. Wingate MS, Alexander GR. The healthy migrant theory: Variations in pregnancy outcomes among US-born migrants. *Soc Sci Med*. 2006;62(2):491-498. doi:10.1016/j.socscimed.2005.06.015
11. Ramraj C, Pulver A, Siddiqi A. Intergenerational transmission of the healthy immigrant effect (HIE) through birth weight: A systematic review and meta-analysis. *Soc Sci Med*. 2015;146:29-40. doi:10.1016/j.socscimed.2015.10.023
12. Restrepo-Mesa SL, Estrada-Restrepo A, González-Zapata LI, Agudelo-Suárez AA. Newborn Birth Weights and Related Factors of Native and Immigrant Residents of Spain. *J Immigr Minor Health*. 2015;17(2):339-348. doi:10.1007/s10903-014-0089-5
13. Sørbye IK, Vangen S, Juarez SP, et al. Birthweight of babies born to migrant mothers - What role do integration policies play? *SSM Popul Health*. 2019;9:100503. doi:10.1016/j.ssmph.2019.100503
14. Almond D, Currie J. Killing me softly: The fetal origins hypothesis. *Journal of Economic Perspectives*. 2011;25(3):153-172. doi:10.1257/jep.25.3.153
15. Castañeda H, Holmes SM, Madrigal DS, Young MED, Beyeler N, Quesada J. Immigration as a Social Determinant of Health. *Annu Rev Public Health*. 2015;36(1):375-392. doi:10.1146/annurev-publhealth-032013-182419
16. Panico L, Tô M, Thevenon O. What is the link between mothers' level of education and low birthweight? *Population and Societies*. 2015;523(1):1-4.

For peer review only - http://bmjopen.bmj.com/site/about/guidelines.xhtml

17. Auger N, Luo ZC, Platt RW, Daniel M. Do mother's education and foreign born status interact to influence birth outcomes? Clarifying the epidemiological paradox and the healthy migrant effect. *J Epidemiol Community Health (1978)*. 2008;62(5):402-409. doi:10.1136/jech.2007.064535
18. Racape J, Schoenborn C, Sow M, Alexander S, de Spiegelaere M. Are all immigrant mothers really at risk of low birth weight and perinatal mortality? The crucial role of socio-economic status. *BMC Pregnancy Childbirth*. 2016;16(1). doi:10.1186/s12884-016-0860-9
19. Wanner P, Khlat M, Bouchardy C. Health habits and behaviour of Southern European and Nort African immigrants in France. *Rev Epidemiol Sante Publique*. 1995;43(6):548-559.
20. El-Khoury Lesueur F, Sutter-Dallay AL, Panico L, et al. The perinatal health of immigrant women in France: a nationally representative study. *Int J Public Health*. 2018;63(9):1027-1036. doi:10.1007/s00038-018-1146-y
21. Palloni A, Morenoff JD. Interpreting the paradoxical in the hispanic paradox. Demographic and epidemiologic approaches. *Ann N Y Acad Sci*. 2001;954:140-174. doi:10.1111/j.1749-6632.2001.tb02751.x
22. Melchior M, Chollet A, Glangeaud-Freudenthal N, et al. Tobacco and alcohol use in pregnancy in France: The role of migrant status. The nationally representative ELFE study. *Addictive Behaviors*. 2015;51:65-71. doi:10.1016/j.addbeh.2015.07.015
23. Harley K, Eskenazi B. Time in the United States, social support and health behaviors during pregnancy among women of Mexican descent. *Soc Sci Med*. 2006;62(12):3048-3061. doi:10.1016/j.socscimed.2005.11.036
24. Jaddoe VW v., Felix JF, Andersen AMN, et al. The LifeCycle Project-EU Child Cohort Network: a federated analysis infrastructure and harmonized data of more than 250,000 children and parents. *Eur J Epidemiol*. 2020;35(7):709-724. doi:10.1007/s10654-020-00662-z
25. Charles MA, Thierry X, Lanoe JL, et al. Cohort Profile: The French National cohort of children ELFE: birth to 5 years. *Int J Epidemiol*. Published online November 20, 2019. doi:10.1093/ije/dyz227
26. Newnham JP, Evans SF, Michael CA, Stanley FJ, Landau LI. Effects of frequent ultrasound during pregnancy: a randomised controlled trial. *The Lancet*. 1993;342(8876):887-891. doi:10.1016/0140-6736(93)91944-H
27. Wright J, Small N, Raynor P, et al. Cohort Profile: The Born in Bradford multi-ethnic family cohort study. *Int J Epidemiol*. 2013;42(4):978-991. doi:10.1093/ije/dys112
28. van Eijsden M, Vrijkotte TGM, Gemke RJBJ, van der Wal MF. Cohort profile: the Amsterdam Born Children and their Development (ABCD) study. *Int J Epidemiol*. 2011;40(5):1176-1186. doi:10.1093/ije/dyq128
29. Kooijman MN, Kruithof CJ, van Duijn CM, et al. The Generation R Study: design and cohort update 2017. *Eur J Epidemiol*. 2016;31(12):1243-1264. doi:10.1007/s10654-016-0224-9
30. Richiardi L, Baussano I, Vizzini L, Douwes J, Pearce N, Merletti F. Feasibility of recruiting a birth cohort through the Internet: The experience of the NINFEA cohort. *Eur J Epidemiol*. 2007;22(12):831-837. doi:10.1007/s10654-007-9194-2

For peer review only - http://bmjopen.bmj.com/site/about/guidelines.xhtml

31. Farchi S, Forastiere F, Vecchi Brumatti L, et al. Piccolipiù, a multicenter birth cohort in Italy: protocol of the study. *BMC Pediatr*. 2014;14(1):36. doi:10.1186/1471-2431-14-36
32. Guxens M, Ballester F, Espada M, et al. Cohort Profile: the INMA--INfancia y Medio Ambiente--(Environment and Childhood) Project. *Int J Epidemiol*. 2012;41(4):930-940. doi:10.1093/ije/dyr054
33. L'Abee C, Sauer PJ, Damen M, Rake JP, Cats H, Stolk RP. Cohort Profile: The GECKO Drenthe study, overweight programming during early childhood. *Int J Epidemiol*. 2008;37(3):486-489. doi:10.1093/ije/dym218
34. Pizzi C, Richiardi M, Charles MA, et al. Measuring Child Socio-Economic Position in Birth Cohort Research: The Development of a Novel Standardized Household Income Indicator. *Int J Environ Res Public Health*. 2020;17(5):1700. doi:10.3390/ijerph17051700
35. Uphoff EP, Pickett KE, Wright J. Social gradients in health for Pakistani and White British women and infants in two UK birth cohorts. *Ethn Health*. 2016;21(5):452-467. doi:10.1080/13557858.2015.1091442
36. Stanek M, Requena M, Del Rey A, García-Gómez J. Beyond the healthy immigrant paradox: decomposing differences in birthweight among immigrants in Spain. *Global Health*. 2020;16(1):87. doi:10.1186/s12992-020-00612-0
37. Hayes DK, Lukacs SL, Schoendorf KC. Heterogeneity within Asian subgroups: A comparison of birthweight between infants of US and non-us born Asian Indian and Chinese mothers. *Matern Child Health J*. 2008;12(5):549-556. doi:10.1007/s10995-007-0270-8
38. Margetts BM, Yusof SM, Al Dallal Z, Jackson AA. Persistence of lower birth weight in second generation South Asian babies born in the United Kingdom. *J Epidemiol Community Health (1978)*. 2002;56(9):684-687. doi:10.1136/jech.56.9.684
39. Sow M, Schoenborn C, de Spiegelaere M, Racape J. Influence of time since naturalisation on socioeconomic status and low birth weight among immigrants in Belgium. A population-based study. *PLoS One*. 2019;14(8):e0220856. doi:10.1371/journal.pone.0220856

For peer review only

Figure Legend

Fig.1. OLS coefficients for cohorts in which children of immigrants exhibit higher birthweight (in grams)

Fig. 2 OLS regression coefficients for cohorts in which children of immigrants exhibit lower birthweight (in grams)

For peer review only - http://bmjopen.bmj.com/site/about/guidelines.xhtml

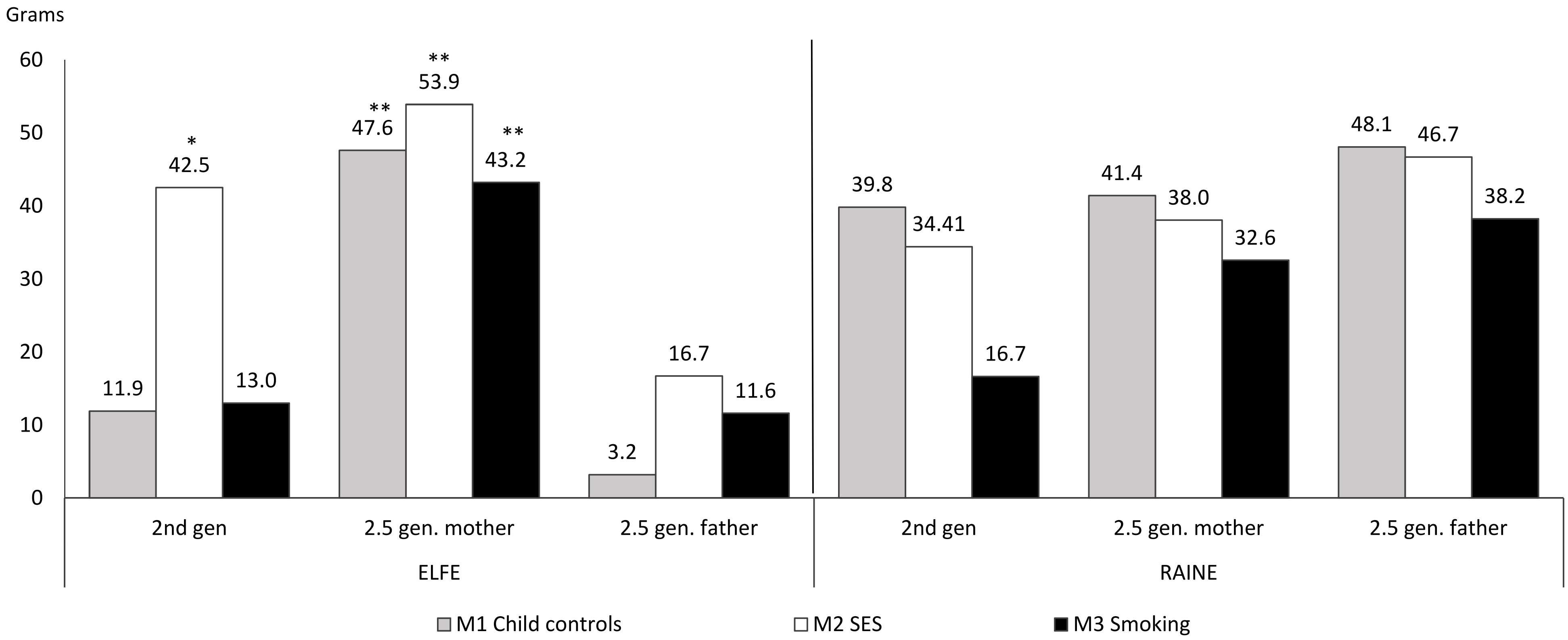

*$p < .05$, **$p < .01$

Fig. 1. OLS coefficients for cohorts in which children of immigrants exhibit higher birthweight (in grams)

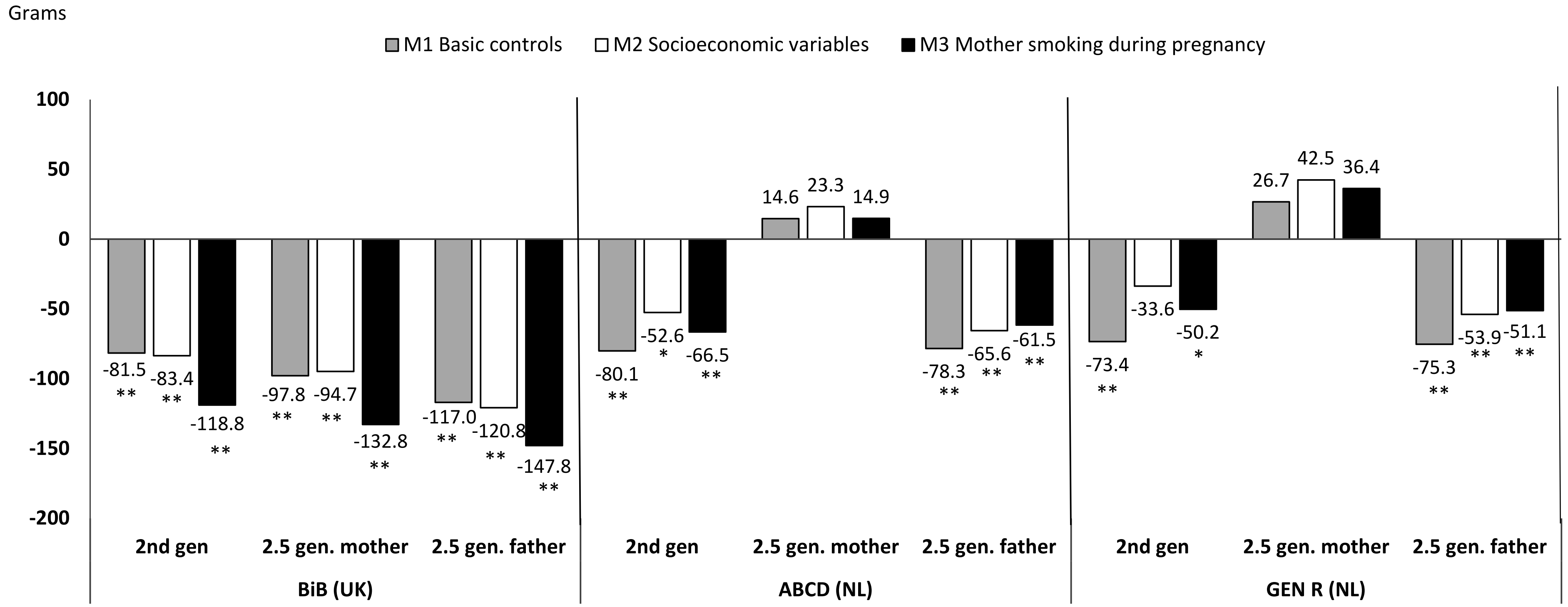

*p < .05, **p <.01

Fig. 2 OLS regression coefficients for cohorts in which children of immigrants exhibit lower birthweight (in grams)

Appendix 1 Cohort sample distribution & children's birthweight (in grams) by migration status and mother's region of origin (cohorts with small samples)

	GECKO (NL)			INMA (SP)			NINFEA (IT)			Piccoli Piu (IT)		
			Weight			Weight			Weight			Weight
	Freq.	%	Mean	Freq.	%	Mean	Freq.	%	Mean	Freq.	%	Mean
**Children's migration status**												
1st generation	0	0	---	0	0	---	16	0.4	3539	0	0	---
2nd generation	26	0.9	3414	125	5.5	3343	28	0.8	3390	123	3.7	3413
2.5 generation (mother)	60	2.1	3428	88	3.9	3321	110	3.0	3294	151	4.5	3376
2.5 generation (father)	64	2.3	3578	117	5.2	3164	95	2.6	3202	106	3.2	3388
natives	2445	86.0	3549	1791	78.9	3252	3348	91.1	3271	2881	85.8	3327
Missing	247	8.7	3484	20	0.9	3486	59	1.6	3240	95	2.8	3297
**Total**	**2842**	**100**	**3540**	**2141**	**100**	**3258**	**3675**	**100**	**3272**	**3356**	**100**	**3334**
**Mother's region of birth**												
Host country-born	2548	89.7	3548	1913	89.4	3247	3516	96.2	3244	3031	90.3	3329
Western EU/EEA	16	0.6	3503	29	1.35	3301	50	1.4	3430	41	1.2	3328
Eastern EU	7	0.2	3546	19	0.89	3186	32	0.9	3628	109	3.3	3426
Other Europe & Central Asia	11	0.4	3558	6	0.28	3127	12	0.3	2550	38	1.1	3421
East Asia & Pacific	18	0.6	3371	1	0.05	3210	1	0	2750	6	0.2	3372
South Asia	7	0.2	3388	0	0	---	1	0	3385	4	0.1	3099
Middle East & North Africa	9	0.3	3490	4	0.19	3415	3	0.1	2945	16	0.5	3427
Sub-Saharan Africa	6	0.2	3501	3	0.14	3122	2	0.1	3253	4	0.1	3239
Latin America & Caribbean	16	0.6	3161	151	7.05	3373	37	1	3225	54	1.6	3338
North America	1	0.0	4460	0	0	---	2	0.1	---	6	0.2	3748
Missing	203	7.1	3495	15	0.7	3481	0	0	3272	47	1.4	3288
**Total**	**2842**	**100**	**3540**	**2141**	**100**	**3258**	**3656**	**100**		**3356**	**100**	**3334**
***n* Missing cases in analyses**	***n***		**Mean**	***n***		**Mean**	***n***		**Mean**	***n***		**Mean**
Birthweight	50		---	21		---	69		---	19		---
Child & mother's controls	438		3486	118		3354	181		3315	50		3409
Migration status	39		3439	7		3294	51		3235	71		3277
Missing other variables	86		3458	97		3059	148		3313	175		3310
**Total excluded cases**	**613**		**3479**	**243**		**3222**	**362**		**3304**	**315**		**3319**

For peer review only

Appendix 2 OLS regression coefficients of mother's region of origin & child's migrant status on child's birthweight (Small cohorts - extracts)

	GECKO (NL)			INMA (SP)		
**DV1: Birthweight**	**M1**	**M2**	**M3**	**M1**	**M2**	**M3**
	**Child controls**	**SES**	**Smoke**	**Child controls**	**SES**	**Smoke**
**Child's migrant status**						
Natives (ref.)						
1st Generation	-	-	-	-	-	-
2nd Generation	-139.76	-117.46	-156.54	162.68 ***	169.51 ***	143.41 ***
2.5 Generation - mother	101.21	99.87	79.96	70.50	78.87	75.32
2.5 generation - father	16.45	16.31	12.30	21.46	28.86	29.50
**Constant**	**-5767.43** ***	**-5738.24** ***	**-5690.72** ***	**-4847.76** ***	**-4734.01** ***	**-4723.00** ***
**N**	**2229**	**2229**	**2229**	**1898**	**1898**	**1898**
**R2**	0.4023	0.4057	0.4238	0.333	0.338	0.344
**Mother's region of origin**						
Host country-born (ref.)						
Western EU/EEA	49.22	61.92	60.12	-31.75	-14.98	-22.09
Eastern EU	176.27	171.70	147.02	175.44	182.01	168.67
Other Europe & Central Asia	255.46	277.51	260.90	-122.58	-110.36	-106.48
East Asia & Pacific	21.84	21.53	2.13	-	-	-
South Asia	113.32	148.45	88.23	-	-	-
Middle East & North Africa	1.50	34.60	-16.44	-	-	-
Sub-Saharan Africa	58.96	83.10	39.75	-	-	-
Latin America & Caribbean	-146.75	-161.48	-196.00	173.31 ***	176.04 ***	155.59 ***
North America	-	-	-	-	-	-
**Constant**	**-5757.77** ***	**-5737.15** ***	**-5671.80** ***	**-4892.39** ***	**-4773.55** ***	**-4763.82** ***
**N**	**2,254**	**2,254**	**2,254**	**1898**	**1898**	**1898**
**R2**	0.4021	0.4059	0.4238	0.336	0.34	0.346

**p < .05, **p < .01, ***p < .001.*

Piccolopiù used a binary variable for parity and : Region 1: Western Europe, Region 2: Easter Europe + Other Europe & Central Asia, Region 3: Latin America & Caribbean, Region 4: other regions.

INMA East Asia & Pacific includes Middle East & North Africa and Sub-Saharan Africa due to small number sizes.

M1: Basic child et mother's controls at birth

M2: M1 + socioeconomic variables

M3: M2 + mother's smoking during pregnancy

Appendix 2 Continued

	NINFEA (IT)			PICCOLOPIU (IT)		
**DV1: Birthweight**	**M1**	**M2**	**M3**	**M1**	**M2**	**M3**
	**Child controls**	**SES**	**Smoke**	**Child controls**	**SES**	**Smoke**
**Child's migrant status**						
Natives (ref.)						
1st Generation	153.43	147.14	147.89	-	-	-
2nd Generation	118.20	109.99	108.60	118.49 ***	100.96 **	97.18 **
2.5 Generation - mother	41.05	40.23	39.97	56.51	54.19	53.27
2.5 generation - father	-70.12	-73.01	-74.15	20.68	15.51	14.58
**Constant**	**-3887.01** ***	**-3883.26** ***	**-3873.41** ***	**-4584.30** ***	**-4521.88** ***	**-4539.19** ***
**N**	**3216**	**3216**	**3216**	**3041**	**3041**	**3041**
**R2**	0.311	0.312	0.314	0.306	0.308	0.309
**Mother's region of origin**						
Host country-born (ref.)						
Western EU/EEA	-10.95	-4.73	-4.85	36.70	34.20	32.98
Eastern EU	124.89 *	123.63 *	126.67 *	116.58 **	103.41 **	102.47 **
Other Europe & Central Asia	96.77	100.00	97.25			
East Asia & Pacific	-62.01	-58.73	-12.27	65.62	57.29	51.86
South Asia	-6.17	-10.28	-15.99			
Middle East & North Africa	45.25	58.64	53.82			
Sub-Saharan Africa	-187.85	-191.41	-196.06			
Latin America & Caribbean	80.17	79.42	76.13	96.08	87.84	88.07
North America	125.89	134.27	128.67	-	-	-
**Constant**	**-4179.89** ***	**-4185.06** ***	**-4182.61** ***	**-4526.39** ***	**-4459.02** ***	**-4477.76** ***
**N**	**5872**	**5872**	**5872**	**3070**	**3070**	**3070**
**R2**	0.408	0.409	0.41	0.304	0.306	0.307

**p < .05, **p < .01, ***p < .001.*

Appendix 3 OLS regression coefficients of children's migration status on child's birthweight (DV2: excluded birthweight > 4,500g; large cohorts)

	ELFE (FR)			RAINE STUDY (AU)			BIB (UK)		
	**M1**	**M2**	**M3**	**M1**	**M2**	**M3**	**M1**	**M2**	**M3**
**DV2: Birthweight**	**Child controls**	**SES**	**Smoke**	**Child controls**	**SES**	**Smoke**	**Child controls**	**SES**	**Smoke**
**Children's migration status**									
Natives (ref.)									
2nd generation	16.4	45.7 **	17.1	46.9 *	40.6	23.8	-80.6 ***	-81.3 ***	-115.9 ***
2.5 generation (mother)	40.4 **	46.2 **	35.8 *	44.8	41.2	36.4	-99.6 ***	-95.9 ***	-133.0 ***
2.5 generation (father)	4.5	17.3	12.3	41.5	39.6	32.0	-108.3 ***	-112.0 ***	-138.2 ***
**Child controls**									
Female	-141.2 ***	-141 ***	-142.1 ***	-113.1 ***	-113.0 ***	-123.3 ***	-131.9 ***	-130.3 ***	-131.5 ***
Plural birth[a]	-372.8 ***	-374.7 ***	-378.4 ***	0	0	0	-310.6 ***	-303.6 ***	-308.5 ***
Mother's parity (birth order)	45.7 ***	50.7 ***	49.8 ***	51.4 ***	51.9 ***	64.7 ***	49.5 ***	56.9 ***	54.2 ***
Gestational age	23.1 ***	22.9 ***	22.9 ***	19.3 ***	19.3 ***	20.1 ***	24.9 ***	25.0 ***	24.9 ***
**Mother controls**									
Height	8.3 ***	7.7 ***	7.9 ***	10.9 ***	10.6 ***	10.8 ***	10.9 ***	10.6 ***	10.8 ***
Pre-pregnancy weight	4.3 ***	4.7 ***	4.5 ***	4.9 ***	5.0 ***	4.9 ***	4.8 ***	4.8 ***	4.8 ***
**SES**									
**Education[b]**									
High (ref.)									
Medium		-15.26	-7.13		12.5	13.9		2.50	4.55
Low		-38.16 *	-19.78		-11.3	7.9		-15.80	-12.71
**Household income quintiles[c]**									
1st quintiles (ref.)									
2nd quintile		46.1 ***	39.1 ***					2.8	-7.8
3th quintile		45.5 ***	29 *		-6.2	3.8		-35.8	-21.1
4th quintile		52.9 ***	33.3 *		-19.2	-3.9		-42.6 *	-31.6
5th quintile		57.3 ***	33.9 *		-23.9	6.2		-70.3 **	-34.0
**Mother smoked (pregnancy)**			-114.6 ***			-140.6 ***			-130.9 ***
**Constant**	**-4729** ***	**-4666** ***	**-4645** ***	**-4058** ***	**-4004** ***	**-4030** ***	**-5699** ***	**-5638** ***	**-5594** ***
N	**12373**	**12373**	**12373**	2242	2242	2242	4090	4090	4090
**R^2**	0.36	0.36	0.37	0.38	0.38	0.40	0.49	0.49	0.50

**p* < .05, ***p* < .01, ****p* < .001

[a] The ABCD & RAINE STUDY cohorts only included single births in their sample.

[b] Level of education based on the highest on-going or completed education when the child was 0 year old (between >-1 year and <1 year). If more than one education level is reported within the defined time frame, we used highest recorded education level.

Classification according to International Standard Classification of Education 97/2011 (ISCED-97/2011)

High: Short cycle tertiary, Bachelor, Masters, Doctoral or equivalent (ISCED-2011: 5-8, ISCED-97: 5-6)

Medium: Upper secondary, Post-secondary non-tertiary (ISCED-2011: 3-4, ISCED-97: 3-4)

Low: No education; early childhood; pre-primary; primary; lower secondary or second stage of basic education (ISCED-2011: 0-2, ISCED-97: 0-2)

[c] Eusilc income quintiles for all cohorts except for RAINE (AU) and ABCD (NL) that measured household income in quartiles.

M1b: Basic child et mother's controls at birth

M2b: M1b + socioeconomic variables

M3b: M2b + mother's smoking during pregnancy

Appendix 3 Continued

	ABCD (NL)			GEN R (NL)		
	M1	M2	M3	M1	M2	M3
**DV2: Birthweight**	Child controls	SES	Smoke	Child controls	SES	Smoke
**Children's migration status**						
Natives (ref.)						
2nd generation	-84.7 ***	-58.9 **	-71.3 ***	-69.35 ***	-33.1	-48.1 *
2.5 generation (mother)	19.8	27.5	20.1	36.18	48.2 *	42.6 *
2.5 generation (father)	-73.2 ***	-61.1 **	-57.8 **	-61.37 ***	-41.1 *	-38.5 *
**Child controls**						
Female	-103.2 ***	-104.7 ***	-104.9 ***	-91.9 ***	-92.4 ***	-93.4 ***
Plural birth[a]	0	0	0	-405.8 ***	-409.5 ***	-409.9 ***
Mother's parity (birth order)	72.8 ***	74.7 ***	73.8 ***	98.8 ***	102.2 ***	99.9 ***
Gestational age	24.4 ***	24.4 ***	24.3 ***	24.9 ***	24.8 ***	24.7 ***
**Mother controls**						
Height	8.8 ***	8.0 ***	8.0 ***	9.3 ***	8.4 ***	8.5 ***
Pre-pregnancy weight	3.8 ***	4.1 ***	4.1 ***	3.9 ***	4.1 ***	4.1 ***
**SES**						
**Education[b]**						
High (ref.)						
Medium		-54.6 ***	-43.8 **		-11.0	-8.7
Low		-59.6 **	-38.1		-19.9	-12.9
**Household income quintiles[c]**						
1st quintiles (ref.)						
2nd quintile		5.0	7.9		27.4	21.9
3th quintile		-6.9	2.0		60.3 **	48.5 *
4th quintile		-24.1	-12.9		85.2 **	66.5 *
5th quintile		-	-		79.2 *	60.2
**Mother smoked (pregnancy)**			-119.0 ***			-71.2 ***
**Constant**	**-5090** ***	**-4941** ***	**-4925** ***	**-5462** ***	**-5338** ***	**-5304** ***
N	3,918	3,918	3,918	4751	4751	4751
**R^2**	0.40	0.40	0.41	0.46	0.46	0.47

**p* < .05, ***p* < .01, ****p* < .001

only

Appendix 4 Logistic regression odds ratios of children's migration status on child's birthweight (DV3: low birthweight; large cohorts)

DV3: Low birthweight	ELFE (FR)			RAINE STUDY (AU)			BIB (UK)		
	**M1**	**M2**	**M3**	**M1**	**M2**	**M3**	**M1**	**M2**	**M3**
	**Child controls**	**SES**	**Smoke**	**Child controls**	**SES**	**Smoke**	**Child controls**	**SES**	**Smoke**
**Children's migration status**									
Natives (ref.)									
2nd generation	-0.38	-0.61	-0.35	-0.68 *	-0.60	-0.50	0.39	0.01	0.01
2.5 generation (mother)	-0.26	-0.35	-0.20	-0.06	0.01	0.06	0.23	0.00	0.01
2.5 generation (father)	-0.11	-0.18	-0.14	-0.17	-0.11	-0.05	0.85 ***	0.03 **	0.03 ***
**Child controls**									
Female	0.63 ***	0.63 ***	0.65 ***	0.21	0.22	0.20	-0.44 **	0.01 *	0.02 **
Plural birth[a]	1.59 ***	1.60 ***	1.66 ***	0.00	0.00	0.00	1.45 ***	0.27 ***	0.27 ***
Mother's parity (birth order)	-0.14 *	-0.20 **	-0.21 **	-0.32 **	-0.31 **	-0.32 **	-0.14 .	-0.02 ***	-0.01 ***
Gestational age	-0.15 ***	-0.15 ***	-0.15 ***	-0.12 ***	-0.12 ***	-0.12 ***	-0.15 ***	-0.01 ***	-0.01 ***
**Mother controls**									
Height	-0.03 **	-0.02 *	-0.02 *	-0.06 **	-0.06 **	-0.06 **	-0.06 ***	0.00 ***	0.00 ***
Pre-pregnancy weight	-0.02 ***	-0.02 ***	-0.02 ***	0.00	0.00	0.00	-0.02 **	0.00 *	0.00 *
**SES**									
**Education[b]**									
High (ref.)									
Medium		0.16	0.11		0.09	0.07		-0.01	-0.01
Low		0.50 *	0.38		0.36	0.25		0.00	0.00
**Household income quintiles[c]**									
1st quintiles (ref.)									
2nd quintile		-0.25	-0.20					-0.01	-0.01
3th quintile		-0.24	-0.10		0.02	-0.07		0.01	0.01
4th quintile		-0.31	-0.11		-0.31	-0.43		0.01	0.01
5th quintile		-0.53 *	-0.32		-0.03	-0.26		0.01	0.00
**Mother smoked (pregnancy)**			0.82 ***			0.72 **			0.01
**Constant**	**41.97** ***	**41.23** ***	**41.62** ***	**37.64** ***	**37.37** ***	**37.77** ***	**49.40** ***	**3.53** ***	**3.53** ***
N	12494	12494	12494	2283	2283	2283	4132	4132	4132
**ll**	-1294	-1283.8	-1265.3	-276.92	-269.25	-263.76			

**p* < .05, ***p* < .01, ****p* < .001*

[a] *The ABCD & RAINE STUDY cohorts only included single births in their sample.*

[b] *Level of education based on the highest on-going or completed education when the child was 0 year old (between >-1 year and <1 year). If more than one education level is reported within the defined time frame, we used highest recorded education level.*

Classification according to International Standard Classification of Education 97/2011 (ISCED-97/2011)

High: Short cycle tertiary, Bachelor, Masters, Doctoral or equivalent (ISCED-2011: 5-8, ISCED-97: 5-6)

Medium: Upper secondary, Post-secondary non-tertiary (ISCED-2011: 3-4, ISCED-97: 3-4)

Low: No education; early childhood; pre-primary; primary; lower secondary or second stage of basic education (ISCED-2011: 0-2, ISCED-97: 0-2)

[c] *Eusilc income quintiles for all cohorts except for RAINE (AU) and ABCD (NL) that measured household income in quartiles.*

M1b: Basic child et mother's controls at birth

M2b: M1b + socioeconomic variables

M3b: M2b + mother's smoking during pregnancy

Appendix 4 Continued

DV3: Low birthweight	ABCD (NL)			GEN R (NL)		
	M1 Child controls	M2 SES	M3 Smoke	M1 Child controls	M2 SES	M3 Smoke
**Children's migration status**						
Natives (ref.)						
2nd generation	0.67 *	0.42	0.61	0.37	0.37	0.44
2.5 generation (mother)	-0.35	-0.39	-0.30	-0.18	-0.14	-0.16
2.5 generation (father)	0.43	0.25	0.28	0.58 *	0.56 *	0.53 *
**Child controls**						
Female	0.63 **	0.63 **	0.68 **	0.39 *	0.38 *	0.38 *
Plural birth[a]	0.00	0.00	0.00	2.05 ***	2.03 ***	2.03 ***
Mother's parity (birth order)	-0.55 ***	-0.57 ***	-0.55 **	-0.37 **	-0.39 **	-0.37 *
Gestational age	-0.16 ***	-0.16 ***	-0.16 ***	-0.16 ***	-0.16 ***	-0.16 ***
**Mother controls**						
Height	-0.04 *	-0.03	-0.04	-0.05 ***	-0.04 **	-0.05 **
Pre-pregnancy weight	-0.01	-0.01	-0.01	-0.02 *	-0.02 *	-0.02 *
**SES**						
**Education**[b]						
High (ref.)						
Medium		0.32	0.13		-0.26	-0.26
Low		0.18	-0.12		-1.16 *	-1.19 *
**Household income quintiles**[c]						
1st quintiles (ref.)						
2nd quintile		0.12	0.10		-0.07	-0.05
3th quintile		0.68 *	0.60		-0.33	-0.27
4th quintile		0.48	0.41		-1.07 *	-0.96 *
5th quintile					-0.34	-0.23
**Mother smoked (pregnancy)**			1.28 ***			0.31
**Constant**	**45.15** ***	**44.16** ***	**45.66** ***	**48.74** ***	**48.80** ***	**48.87** ***
N	4,032	4,032	4,032	4877	4877	4877
**ll**	-325.45	-321.52	-313.42	-480.1	-473.67	-472.55

**p* < .05, ***p* < .01, ****p* < .001*

only

STROBE Statement—Checklist of items that should be included in reports of ***cohort studies***

		Item No	Recommendation
**Title and abstract**	p.1	1	(*a*) Indicate the study's design with a commonly used term in the title or the abstract
			(*b*) Provide in the abstract an informative and balanced summary of what was done and what was found
**Introduction**			
Background/rationale	p.2	2	Explain the scientific background and rationale for the investigation being reported
Objectives	p. 4	3	State specific objectives, including any prespecified hypotheses
**Methods**			
Study design	p.4	4	Present key elements of study design early in the paper
Setting	p.4-5	5	Describe the setting, locations, and relevant dates, including periods of recruitment, exposure, follow-up, and data collection
Participants	p.4-5	6	(*a*) Give the eligibility criteria, and the sources and methods of selection of participants. Describe methods of follow-up
			(*b*) For matched studies, give matching criteria and number of exposed and unexposed
Variables	p.5-6	7	Clearly define all outcomes, exposures, predictors, potential confounders, and effect modifiers. Give diagnostic criteria, if applicable
Data sources/ measurement	p. 5-6	8*	For each variable of interest, give sources of data and details of methods of assessment (measurement). Describe comparability of assessment methods if there is more than one group
Bias	p.7	9	Describe any efforts to address potential sources of bias
Study size	p.7	10	Explain how the study size was arrived at
Quantitative variables		11	Explain how quantitative variables were handled in the analyses. If applicable, describe which groupings were chosen and why
Statistical methods	p.5-6	12	(*a*) Describe all statistical methods, including those used to control for confounding
			(*b*) Describe any methods used to examine subgroups and interactions
			(*c*) Explain how missing data were addressed
			(*d*) If applicable, explain how loss to follow-up was addressed
			(*e*) Describe any sensitivity analyses
**Results**			
Participants	p.7	13*	(a) Report numbers of individuals at each stage of study—eg numbers potentially eligible, examined for eligibility, confirmed eligible, included in the study, completing follow-up, and analysed
			(b) Give reasons for non-participation at each stage
			(c) Consider use of a flow diagram
Descriptive data	p.8	14*	(a) Give characteristics of study participants (eg demographic, clinical, social) and information on exposures and potential confounders
			(b) Indicate number of participants with missing data for each variable of interest
			(c) Summarise follow-up time (eg, average and total amount)
Outcome data	p.8	15*	Report numbers of outcome events or summary measures over time
Main results	p.8-11	16	(*a*) Give unadjusted estimates and, if applicable, confounder-adjusted estimates and their precision (eg, 95% confidence interval). Make clear which confounders were adjusted for and why they were included
			(*b*) Report category boundaries when continuous variables were categorized
			(*c*) If relevant, consider translating estimates of relative risk into absolute risk for a meaningful time period

For peer review only

Other analyses	p.8, Appendix 3-4	17	Report other analyses done—eg analyses of subgroups and interactions, and sensitivity analyses
**Discussion**			
Key results	p.12	18	Summarise key results with reference to study objectives
Limitations	p.13-14	19	Discuss limitations of the study, taking into account sources of potential bias or imprecision. Discuss both direction and magnitude of any potential bias
Interpretation	p.13-14	20	Give a cautious overall interpretation of results considering objectives, limitations, multiplicity of analyses, results from similar studies, and other relevant evidence
Generalisability	p.13-14	21	Discuss the generalisability (external validity) of the study results
**Other information**			
Funding	p.16-17	22	Give the source of funding and the role of the funders for the present study and, if applicable, for the original study on which the present article is based

*Give information separately for exposed and unexposed groups.

Note: An Explanation and Elaboration article discusses each checklist item and gives methodological background and published examples of transparent reporting. The STROBE checklist is best used in conjunction with this article (freely available on the Web sites of PLoS Medicine at http://www.plosmedicine.org/, Annals of Internal Medicine at http://www.annals.org/, and Epidemiology at http://www.epidem.com/). Information on the STROBE Initiative is available at http://www.strobe-statement.org.

For peer review only
